# Development of Selective Phosphatidylinositol 5-Phosphate 4-Kinase γ Inhibitors with a Non-ATP-competitive, Allosteric Binding Mode

**DOI:** 10.1021/acs.jmedchem.1c01819

**Published:** 2022-02-11

**Authors:** Helen K. Boffey, Timothy P. C. Rooney, Henriette M. G. Willems, Simon Edwards, Christopher Green, Tina Howard, Derek Ogg, Tamara Romero, Duncan E. Scott, David Winpenny, James Duce, John Skidmore, Jonathan H. Clarke, Stephen P. Andrews

**Affiliations:** † The ALBORADA Drug Discovery Institute, University of Cambridge, Island Research Building, Cambridge Biomedical Campus, Hills Road, Cambridge CB2 0AH, U.K.; ‡ UK Dementia Research Institute, University of Cambridge, Island Research Building, Cambridge Biomedical Campus, Hills Road, Cambridge CB2 0AH, U.K.; § Peak Proteins, Alderley Park, Macclesfield SK10 4TG, Cheshire, U.K.

## Abstract

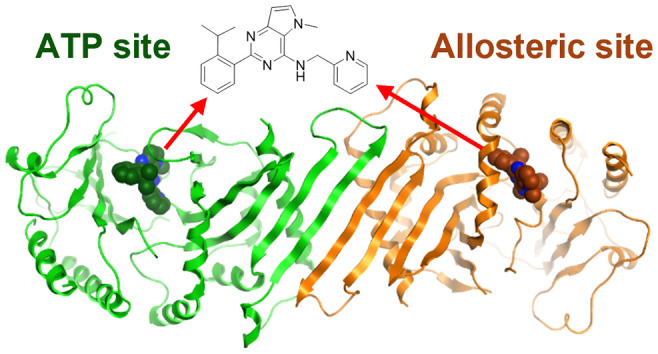

Phosphatidylinositol 5-phosphate 4-kinases (PI5P4Ks) are emerging as attractive therapeutic targets in diseases, such as cancer, immunological disorders, and neurodegeneration, owing to their central role in regulating cell signaling pathways that are either dysfunctional or can be modulated to promote cell survival. Different modes of binding may enhance inhibitor selectivity and reduce off-target effects in cells. Here, we describe efforts to improve the physicochemical properties of the selective PI5P4Kγ inhibitor, NIH-12848 (**1**). These improvements enabled the demonstration that this chemotype engages PI5P4Kγ in intact cells and that compounds from this series do not inhibit PI5P4Kα or PI5P4Kβ. Furthermore, the first X-ray structure of PI5P4Kγ bound to an inhibitor has been determined with this chemotype, confirming an allosteric binding mode. An exemplar from this chemical series adopted two distinct modes of inhibition, including through binding to a putative lipid interaction site which is 18 Å from the ATP pocket.

## Introduction

A large, heterogeneous group of kinase and phosphatase enzymes is responsible for the interconversion of different phosphoinositide lipids present in cells, and these are involved in nearly all the aspects of cell physiology.^1^ Lipid mediators have key functions in membrane trafficking, channel regulation, cell proliferation, and cell stress/death responses and hence are well-documented in diseases such as cancer, developmental disorders (including channelopathies and ciliopathy syndromes), bacterial and viral infections (including hepatitis C and coronavirus), and neurodegeneration.^2−5^

One class of these enzymes, the phosphatidylinositol 5-phosphate 4-kinases (PI5P4Ks), is functionally expressed in mammals to regulate cellular levels of their substrate, PI5P, or generate specific pools of the PI(4,5)P_2_ product.^1,6,7^ The α, β, and γ PI5P4K isoforms have been associated with a wide range of physiological roles including insulin signaling, receptor recycling, gene regulation, and cell stress responses.^8−11^ This has led to specific implications for PI5P4Ks in diseases, especially in cancer, where all the three isoforms have been found to be upregulated.^12−14^ Studies with knockout mice have also shown that PI5P4Kβ deletion leads to an insulin sensitivity phenotype^15^ and that the deletion of PI5P4Kγ results in immune hyperactivity indicative of autoimmune disease.^16^ Interestingly, this latter observation was mediated through the mechanistic target of rapamycin (mTOR) complex regulation, which has also been associated with PI5P4K activity.^17,18^

The role of the PI5P4Ks in response to cell starvation, a process that leads to the negative regulation of mTORC1 activity to initiate autophagy, has also been documented.^19−22^ The regulation of mTORC1 by PI5P4Kγ results in the basal activation of the complex, whereas reduced PI5P4Kγ activity in starvation conditions initiates autophagy.^19^ Al-Ramahi and co-workers were able to show physiological relevance to neurodegenerative diseases using pharmacological inhibition to reduce the levels of mutant huntingtin protein in fibroblasts from Huntington disease patients and aggregated protein in a neuronal model and that this effect was coincident with increased autophagic flux and specific to the PI5P4Kγ isoform.^23^ PI5P4Kγ is therefore a target for small molecule inhibition in the context of neurodegenerative diseases.

**Figure 1:**
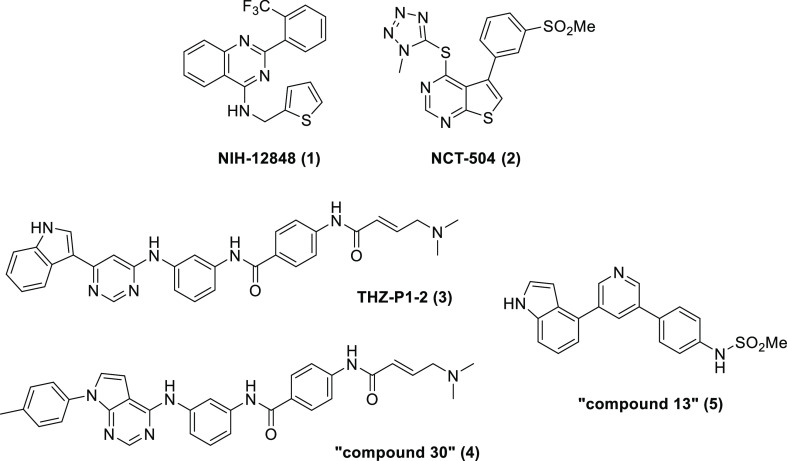
Structures of PI5P4Kγ inhibitors.

The role of PI5P4Kγ in disease makes it a potentially important therapeutic target; however, development has been hampered by the lack of potent and specific inhibitors. Furthermore, in contrast to the protein kinase family, which has a relatively well-conserved active site,^24^ the more diverse lipid kinase family has less structural similarity,^2,25,26^ hindering rational structure-based design. Despite the lack of structural similarity between lipid and protein kinases, a comprehensive study of the selectivity profiles of protein kinase inhibitors previously showed that some exhibit weak activity toward the lipid PI5P 4-kinases,^27^ while other publications have shown that some protein kinase inhibitors display activity against PI5P4Kγ, for example, tyrphostin^28^ and palbociclib^29^ (see Supporting Information Table S1 for more details from our own screening of selected PI5P4Kγ inhibitors). Most significantly, NIH-12848 (**1**)^30^ and NCT-504 (**2**)^23^ have been disclosed as selective PI5P4Kγ inhibitors (Figure 1). Compound **1** was reported to have an IC_50_ of 2–3 μM in a radiometric ^32^P-ATP/PI5P incorporation assay (see Supporting Information Table S2). Compound **2** was identified following a high-throughput screen and, when tested against a panel of 442 kinases, was found to have activity against only PI5P4Kγ (IC_50_ = 16 μM in a ^32^P-ATP/PI5P incorporation assay).

In 2020, pan PI5P4K inhibitors were reported, including covalent inhibitors THZ-P1-2 (**3**), “compound **30**” (**4**),^31,32^ and noncovalent “compound **13**” (**5**)^33^ (Figure 1 and Table S1). Compound **4** displayed an IC_50_ of 1.3 μM against PI5P4Kα in a bioluminescent assay and 9.9 μM against PI5P4Kβ in a fluorescence polarization assay, but inhibited PI5P4Kγ by only 22% when tested in a KINOMEscan assay at 1 μM. In the same assays, compound **3** showed similar activity against PI5P4Kα and β but with a more significant PI5P4Kγ inhibition of 91% and a PI5P4Kγ *K*_D_ of 4.8 nM.^34^ Compound **5** has demonstrated similar levels of activity in these PI5P4Kα and β assays (IC_50_ = 2.0 and 22 μM, respectively) and 100% inhibition of PI5P4Kγ in the KINOMEscan assay when tested at 1 μM and a PI5P4Kγ *K*_D_ of 3.4 nM.^34^

Herein, we describe efforts to improve the physicochemical properties of **1** to identify useful tool molecules. Compound **1** is an attractive starting point as it is inherently PI5P4K subtype selective, yet there is scope for development as it is a lipophilic molecule (*c* log *P* 6.5) with poor solubility (<5 μM). Furthermore, **1** has been proposed to interact with an alternative region of the PI5P4Kγ catalytic site, potentially acting as a competitor for the PI5P substrate rather than for ATP binding^30^ offering a unique opportunity to study lipid kinase structural biology.

## Results

The optimization campaign began with simple modifications of the parent molecule **1**, either through purchasing or synthesizing close analogues with subtle changes to the trifluoromethylphenyl group or thiophene (Table 1). To enable direct comparisons, Promega’s ADP-Glo reporter assay was developed using a mutant form of the kinase. PI5P4Kγ-WT (wild-type) has particularly low enzymatic activity, which is not trivial to measure. The conversion of the PI5P4Kγ catalytic site to the corresponding PI5P4Kα G loop sequence has been shown to increase kinase functional activity^25^ and has enabled the development of an inhibition assay with a useable window. The mutations [the insertion of three amino acids (QAR) at 139 plus an additional 11 amino acid mutations, S132L, E133P, S134N, E135D, G136S, D141G, G142A, E156T, N198G, E199G, and D200E] correspond to the PI5P4Kα residues and the resulting PI5P4Kγ construct containing these has been referred to as PI5P4Kγ+.^25^ To mitigate concerns about being misled by the use of the PI5P4Kγ+ construct and the possible introduction of PI5P4Kα activity into the molecules, our screening cascade included the measurement of ADP-Glo activity at PI5P4Kα-WT (and PI5P4Kβ-WT to fully understand emerging selectivity profiles). Moreover, cellular target engagement was periodically determined for exemplars; this determined compound binding to overexpressed PI5P4Kγ-WT in HEK293 cells through changes in target protein thermal stability, monitored using a DiscoverX’s InCell Pulse assay. Furthermore, we established a cell-free thermal shift assay with PI5P4Kγ-WT and determined that this chemical series showed a good correlation for measured Δ*T*_m_ with PI5P4Kγ-WT and the ADP-Glo pIC_50_ with PI5P4Kγ+ (see Supporting Information Figure S1 and Table S3). In general, for this chemical series, we observed a good correlation between PI5P4Kγ+ and PI5P4Kγ-WT binding and the undetectable levels of PI5P4Kα and PI5P4Kβ inhibition.

**Table 1:**
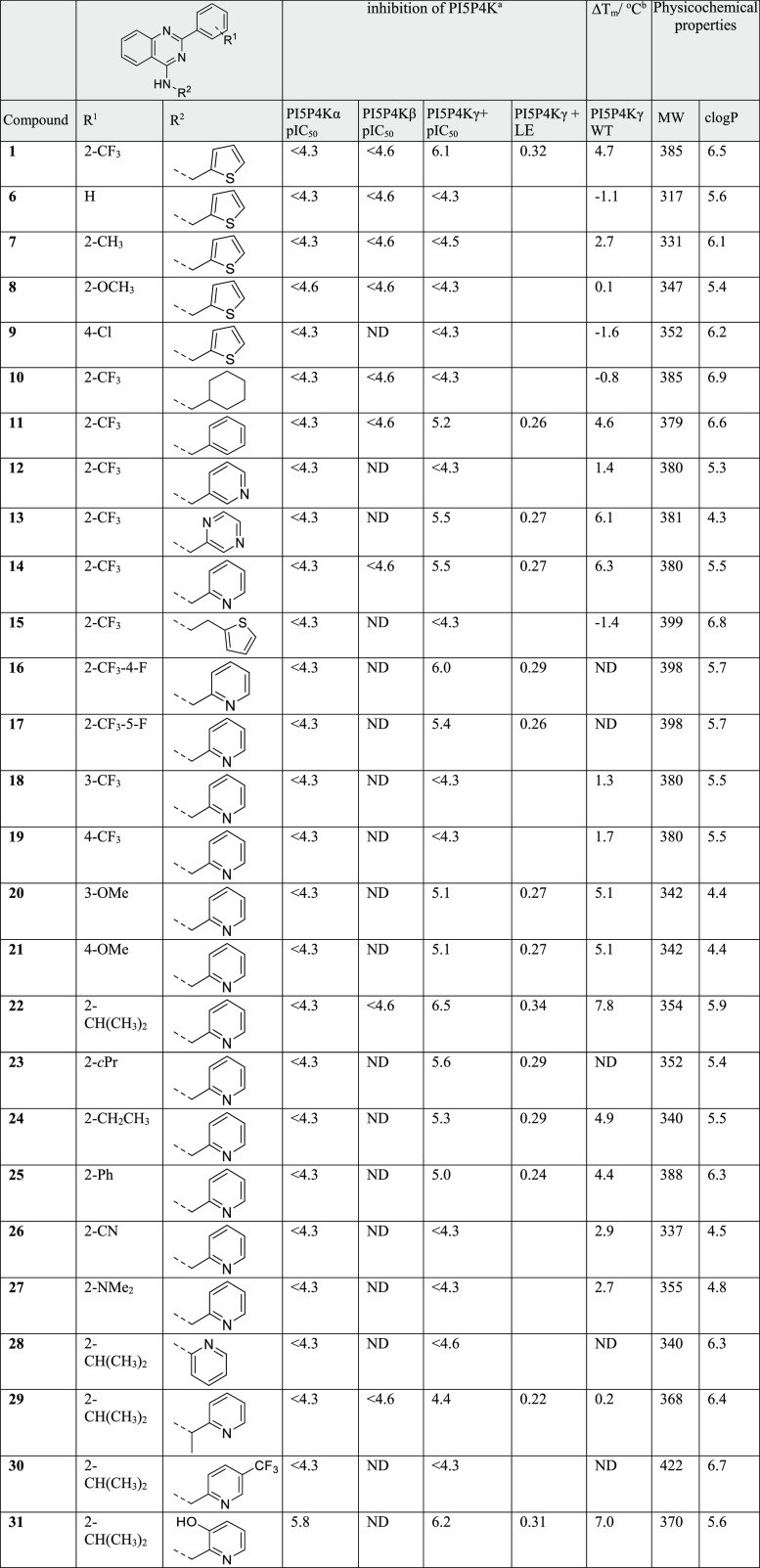
SAR and Physicochemical Properties for the Variation of R^1^ and R^2^

a Determined by ADP-Glo.

b Thermal shift determined with WT proteins, see Supporting Information for further details.

Structural information for each of the PI5P4K isoforms was publicly available at the start of this program in the form of X-ray crystal structures deposited in the Protein Data Bank, but there were no published structures with drug-like ligands bound. The only crystal structure of PI5P4Kγ publicly available at that time (pdb: 2GK9) does not have a ligand bound, but the crystal structures of PI5P4Kβ bound to nucleotides^35^ enabled us to pinpoint the ATP-binding pocket for PI5P4Kγ. However, **1** could not be docked into this pocket in a convincing binding pose, a finding that is consistent with Clarke et al.’s evidence from hydrogen–deuterium exchange-mass spectrometry (HDX-MS)^30^ that this ligand does not bind in the ATP-binding pocket. No other binding pocket for **1** could be identified in the 2GK9 structure, so we proceeded with our attempts to optimize **1** through classical medicinal chemistry approaches without structural guidance.

During initial structure–activity relationship (SAR) scoping with PI5P4Kγ+, a brief scan of a variety of small substituents with different electronic properties at position R^1^ did not reveal any advantages (e.g. **6**–**9**Table 1), and the replacement of the thiophene with other ring systems showed a preference for aromatic rings with a heteroatom at the ortho position (compare **10**–**14**, Table 1). Extending the R^2^ methylene linker to ethylene was not tolerated (**15**) nor was N-methylation (data not shown). On balance, from this initial set of thiophene replacements, pyridine **14** was preferred as it showed a reduced lipophilicity compared to **1** (*c* log *P* 5.5) and a modest improvement in solubility to 9 μM, which resulted in a significant improvement in measured Caco-2 permeability from 6.4 to 85 × 10^–6^ cm/s (Table 2). In the thermal shift assay with PI5P4Kγ-WT protein, **14** showed a Δ*T*_m_ of 6.3 °C versus 4.7 °C for **1** and Δ*T*_m_ < 1 °C against both PI5P4Kα and PI5P4Kβ (Table S4).

**Table 2:** In Vitro ADMET and Physicochemical Properties of Selected Examples

compound	TPSA	*c* log *P*	MW	HLM *t*_1/2_(min)^a^	mPPB (%)^b^	*P*_app_ A → B (10^–6^ cm/s)^c^	ER^c^	solubility (μM)	mchrom log *D*_7.4_^d^
**1**	38	6.5	385	21	>99	6.4	0.9	<5	6.3
**14**	51	5.5	380	31	>99	85	0.4	9	4.9
**22**	51	5.9	354	37	>99	91	0.3	<5	5.7
**35**	51	4.6	344	41	97	106	0.5	113	4.2
**38**	64	4.7	381	100	99	146	0.5	6	3.2
**40**	56	5.2	357	47	99	122	0.4	7	4.5

a Human liver microsomal hepatic stability.

b Mouse plasma protein binding.

c Caco-2 cell permeability, A → B = apical-to-basolateral, and ER = efflux ratio.

d Determined by a high-performance liquid chromatography (HPLC) method.

A further iteration of R^1^ modification was then conducted using the 2-pyridyl analogue **14** as a template. The addition of a fluoro substituent was beneficial for potency at position 4 but not 5 (**16** and **17**). The movement of the trifluoromethyl group from the 2-position to either the 3- or 4-position of the phenyl ring was not tolerated (**18** and **19**, respectively), whereas 3- or 4-methoxy was tolerated to some degree (**20** and **21**, respectively). Small lipophilic substituents gave the greatest PI5P4Kγ+ inhibition when used at position 2, with *i*-Pr > c-Pr > Et > Ph (**22**, **23**, **24**, and **25**, respectively). Indeed, **22** was one of the most ligand efficient (LE) analogues identified during this campaign (LE = 0.34) and showed submicromolar activity in the cellular target engagement assay with PI5P4Kγ-WT (Table 3). However, although significantly improved over **1**, compound **22** still has a high lipophilicity (*c* log *P* = 5.9), which appears to limit its aqueous solubility and microsomal stability (Table 2). Polar groups at position 2 helped to reduce *c* log *P* but were detrimental to potency in the primary assay, including electron-withdrawing groups (**26**) and electron-donating groups (**27**).

**Table 3:**
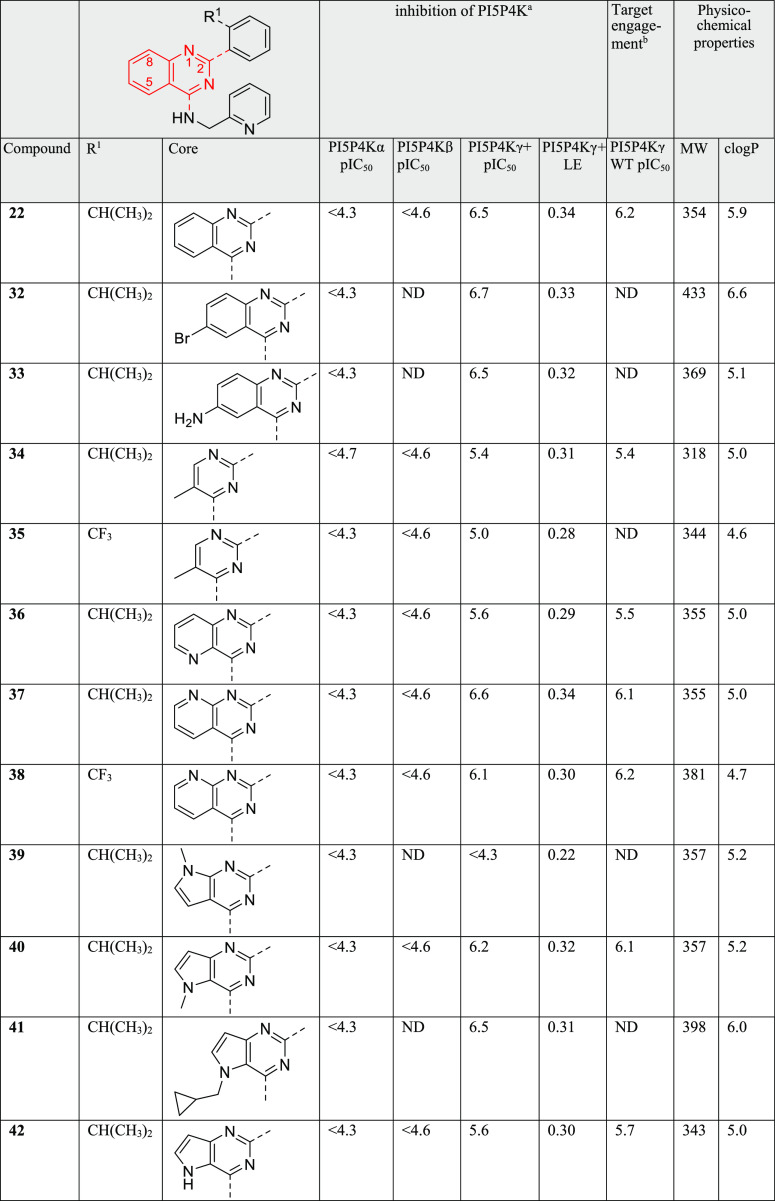
SAR and Physicochemical Properties for the Variation of the Core

a Determined by ADP-Glo.

b Determined by InCell Pulse in intact cells.

Having identified 2-isopropylphenyl as the preferred substituent at position 2 of the quinazoline for potency gains, we next looked at adding further substituents to the 2-pyridyl ring and modifying the quinazoline core. The deletion or branching of the methylene linker (**28** and **29,** respectively) was not tolerated nor was the addition of a 5-trifluoromethyl substituent (**30**). The addition of a 3-hydroxy group (**31**) was also well-tolerated. This modification introduced some inhibition of PI5P4Kα and some polarity that may be of use for further development, but these aspects were not pursued.

The quinazoline core presented a number of opportunities for investigation, including adding substituents, introducing heteroatoms to modulate log *P* and protein contacts, and modifying the ring geometries (Table 3). We generated a series of analogues of **22** to explore this aspect of the SAR. A bromo substituent at position 6 of the quinazoline (**32**) gave a small increase in potency at the cost of significantly increased molecular weight (MW) and *c* log *P*, whereas an amino group at the same position (**33**) maintained activity with a smaller MW penalty and a positive impact on *c* log *P*.

In order to maximize the efficiency of the core, we sought to delete lipophilic atoms or replace them with heteroatoms. Intrigued by the parallels with the work by Dexheimer *et al.*,^36^ and the structural similarity of ML323 (Figure S2), we performed a truncation of the quinazoline core to afford compound **34**. In our assays, ML323 was inactive at both PI5P4Kα and PI5P4Kγ+ (data not shown), whereas **34** showed a pIC_50_ of 5.4 against PI5P4Kγ+ and no detectable inhibition of the α or β isoforms. A similar profile was seen with analogue **35,** possessing a trifluoromethyl instead of the isopropyl (Table 3). The LE of compound **34** was an acceptable 0.31, but this truncation approach was not as productive as the introduction of heteroatoms to the core, at position 5 (**36**) and particularly at position 8 (**37** and **38**). Pyridopyrimidine **37** showed an improved potency, a reduced *c* log *P* and good selectivity versus PI5P4Kα and PI5P4Kβ. Furthermore, **37** showed submicromolar activity in the cellular target engagement assay with PI5P4Kγ-WT.

Regioisomeric pyrrolopyrimidines **39** and **40** were also evaluated. Although compound **39** was inactive, compound **40** showed good potency and LE in the primary assay, and submicromolar activity in the cellular target engagement assay. It also maintained selectivity versus PI5P4Kα and PI5P4Kβ, showed high levels of permeability, and moderate microsomal stability (Table 2). The aliphatic substituent could be extended, as exemplified by **41**, but the deletion of the N-methyl group (**42**) was not well-tolerated.

Binding constants (*K*_D_s) were determined for compound **40** using commercially available assays for PI5P4Kγ-WT (68 nM) and PI5P4Kβ (>30,000 nM) and compared versus compound **1** (Table S6). This assay was not available for PI5P4Kα but a commercial IC_50_ determination was possible via Thermo Fisher to corroborate our own data (Table S7; IC_50_ > 30,000 nM for compounds **1** and **40**). Compound **40** was also tested for selectivity against a panel of 140 protein kinases and 15 lipid kinases (Tables S8 and S9, respectively). Only one of these targets (PAK2) showed <50% residual activity when tested at 10 μM.

**Figure 2:**
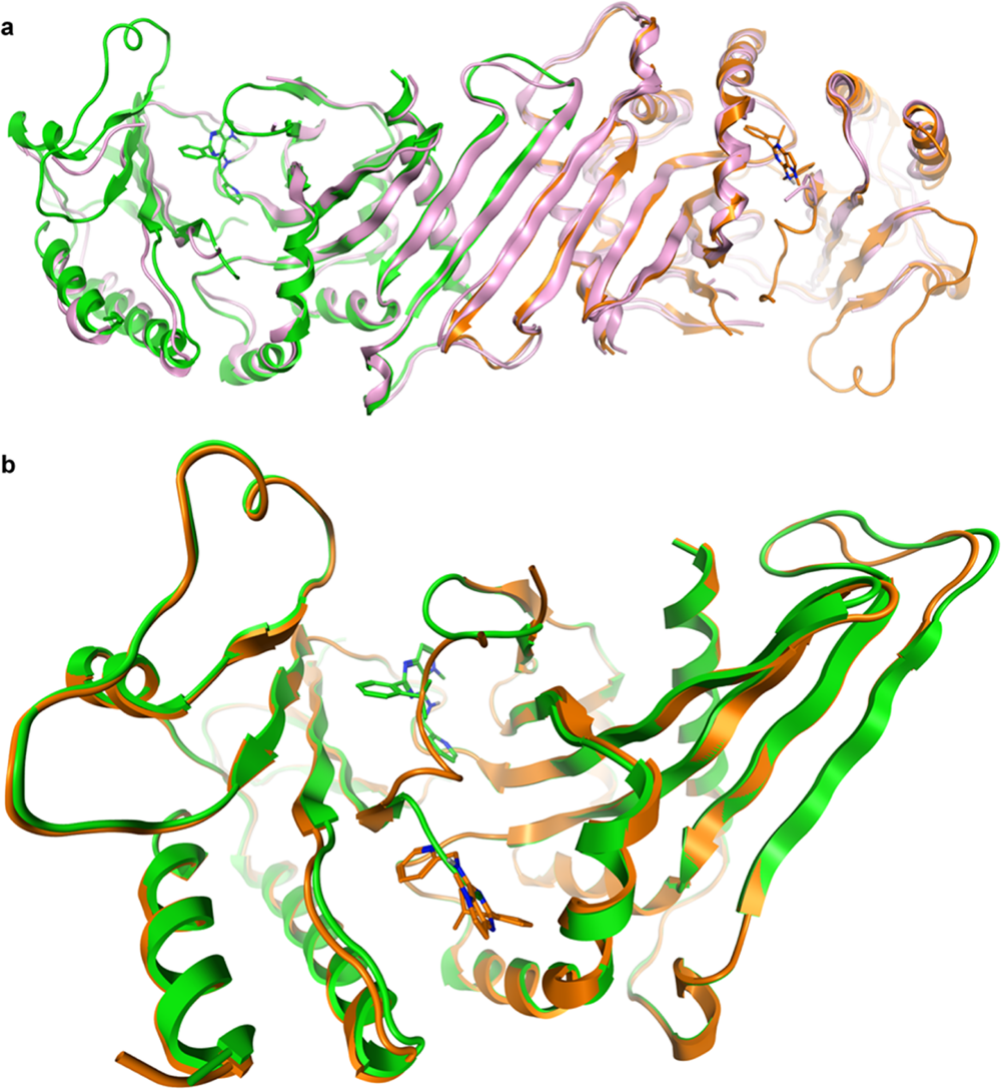
Crystal structure of PI5P4Kγ bound to **40** at 2.4 Å (pdb: 7QIE). (a) Dimer of chains A (orange) and B (green) with **40** bound superposed onto the dimer of chain B and C of the apo PI5P4Kγ structure 2GK9 (in pink) and (b) two binding sites for **40**: chain A (orange, **40** in allosteric binding pocket) and chain B (green, **40** in ATP site) superposed with **40** in the stick. The binding sites are mutually exclusive.

In order to solve the structures of co-complexes of PI5P4Kγ with examples from this chemical series, a number of protein constructs were expressed, purified, and subjected to crystallization trials. A truncated version of human PI5P4Kγ, comprising residues His32 to Ala421, in accordance with the truncation used to generate X-ray structures of the other PI5P4K isoforms, was cloned into a bacterial expression vector. To increase the chances of obtaining crystals, three other truncated constructs were prepared in parallel, each with different length deletions of the previously unstructured region in the PI5P4Kβ structure. The purified recombinant protein was obtained for each of the four different truncations, and all were successful in crystallization trials. Compound **40** was selected for crystallography owing to its balance of potency and solubility. A PI5P4Kγ protein construct, comprising residues His32 to Ala421, with residues 300–341 deleted, was successfully co-crystallized with **40** at a 2.4 Å resolution (Figure 2, pdb code 7QIE).

**Figure 3:**
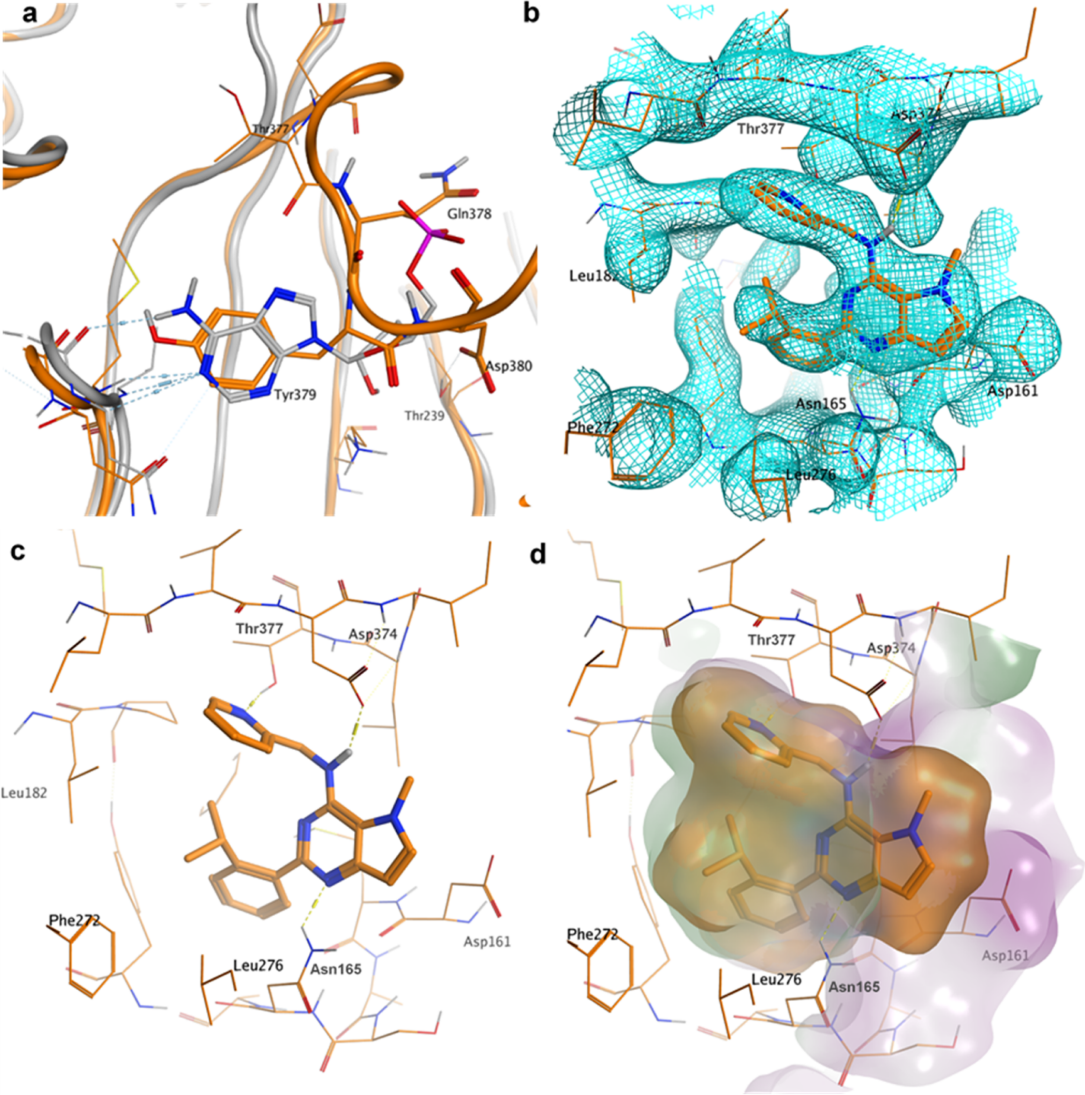
Focus on chain A of the crystal structure of PI5P4Kγ bound to **40** at 2.4 Å with **40** in the allosteric binding pocket (pdb: 7QIE; orange). (a) AMP-binding site of PI5P4Kβ (3X01, gray) superposed onto chain A (orange). Tyr379 of PI5P4Kγ overlays onto the pyrimidine ring of AMP; (b) electron density at 1 σ for the allosteric binding pocket in chain A of the **40**-PI5P4Kγ complex; (c) novel binding pocket in chain A of the **40**-PI5P4Kγ complex with key interactions highlighted; and (d) allosteric binding pocket in chain A of the **40**-PI5P4Kγ complex with ligand and receptor molecular surfaces.

There are two PI5P4Kγ homodimers in the asymmetric unit, as is seen in the 2GK9 apo structure of PI5P4Kγ. Overall, the structure shows a high degree of similarity with that of apo structure 2GK9, although it has increased the structural definition of some of the loop areas in the complex (Figure 2a). The most interesting aspect is the presence of two distinct binding pockets for **40** in PI5P4Kγ that cannot be occupied simultaneously by the ligand (Figure 2b). In chain B, **40** occupies the pocket occupied by AMP/GMP in the PI5P4Kβ crystal structures (pdb codes 3X01, 3X02). The other three monomer chains (A, C, and D) show the ligand located 18 Å away from the ATP site in a lipid-binding pocket.^37,38^ In this protein conformation, residues Gln378, Tyr379, and Asp380 of the activation loop occupy an inhibitory position in the ATP-binding site (Figure 3a). The side chain of Tyr379 superposes closely onto the pyrimidine ring of AMP in 3X01, with the hydroxyl of Tyr379 forming some of the same hydrogen bonds. The side chain of Asp380 interacts with the Thr239 hydroxyl group, mimicking some of the interactions that the ribose of AMP makes. Thus, the binding of **40** into the allosteric pocket appears to stabilize a protein conformation where the activation loop inhibits access to the ATP site.

The allosteric binding site in the PI5P4Kγ protein is formed by residues Asp161, Met162, Asn165, Leu166, Tyr169, Leu182, Phe185, Phe272, Leu273, Leu276, Asp374, and Thr377 with the isopropylphenyl moiety of **40** inserted deep into a mostly lipophilic pocket (Figure 3). The pyridine substituent of **40** forms a hydrogen bond with the Thr377 hydroxyl group, and the central pyrimidine ring forms a hydrogen bond with the side chain of Asn165 (Figure 3c). Finally, the NH linking the pyrrolopyrimidine core with the methylpyridine group interacts with the Asp374 carboxylate group. The ligand fits very snugly into the binding pocket, with only the methyl pyrrole part of the bicyclic core solvent accessible (Figure 3d). The isopropyl group of the ligand is in contact with the pyridine group, thus stabilizing the ligand conformation. In fact, *ab initio* quantum mechanical calculations suggest that the ligand conformation in the lipid-binding site is very close to the lowest energy solution conformation for **40** (RMSD = 0.25 Å).

**Figure 4:**
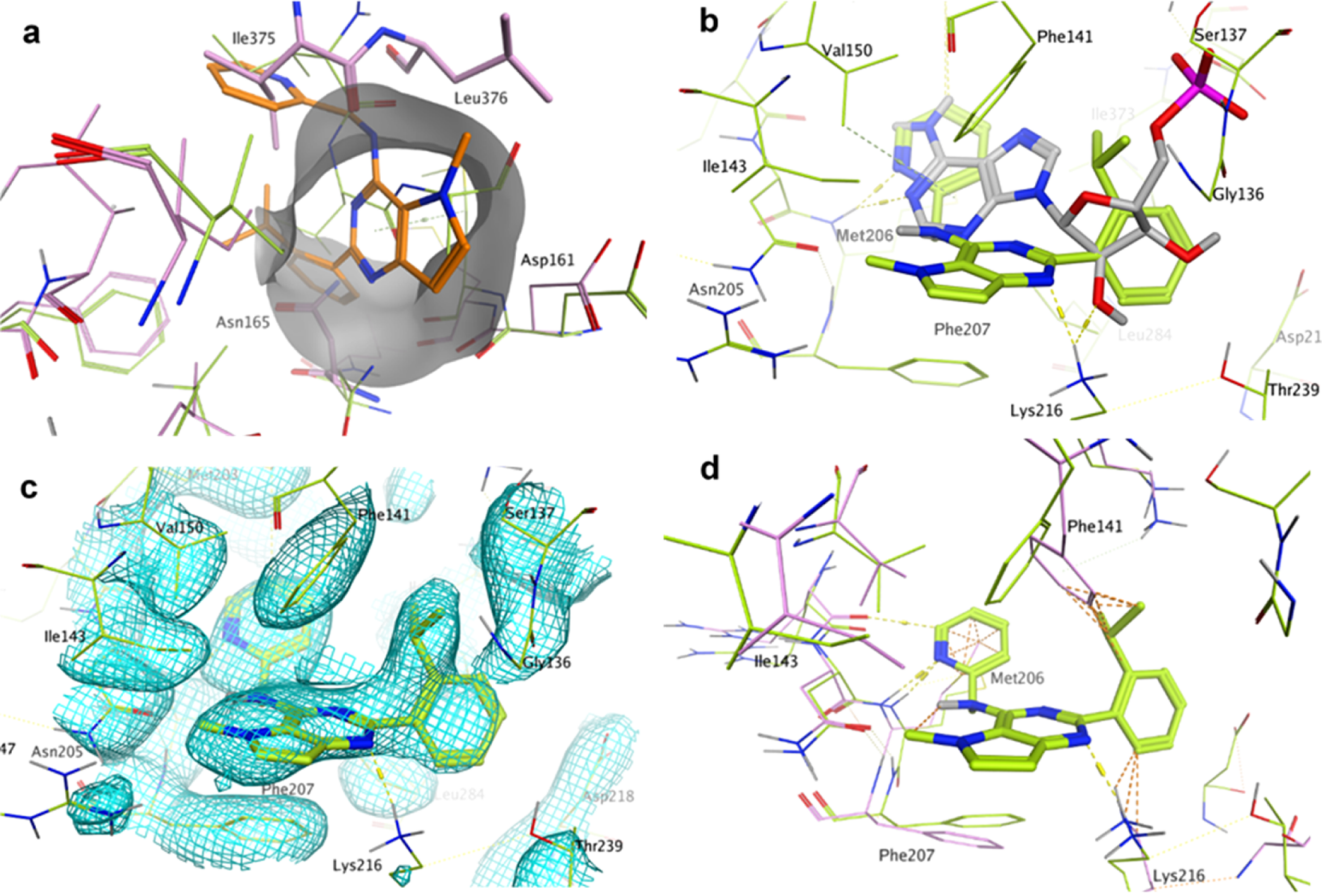
Focus on chain B of the crystal structure of PI5P4Kγ bound to **40** at 2.4 Å (pdb: 7QIE; green). This chain has **40** bound in the ATP pocket. (a) Allosteric binding pocket observed in chains A, C, and D of PI5P4Kγ bound to **40** is (partially) occluded in the apo structure 2GK9 (pink) and chain B of the complex with **40** (green) by residues Ile375, Leu376, and Asn165 (apo only). **40** (orange sticks) from chain A of the PI5P4Kγ-**40** complex is superposed onto apo PI5P4Kγ (pink) and chain B of the PI5P4Kγ-**40** complex (green). (b) **40** bound to the ATP pocket of chain B of the PI5P4Kγ complex in green with AMP as bound to PI5P4Kβ (pdb: 3X01) superposed in gray. The pyrimidine rings do not superpose. (c) Electron density at 1 σ for the ATP-binding pocket in chain B of the **40**-PI5P4Kγ complex. (d) **40** bound to the ATP pocket of chain B of the PI5P4Kγ complex in green with apo PI5P4Kγ (pdb: 2GK9) superposed in pink. Orange dotted lines indicate clashes between the ligand and the apo structure.

In chain B, where the ligand occupies the ATP site, the allosteric binding site is occupied by residues Ile375 and Leu376, which form the start of a loop that is mostly missing in PI5P4K crystal structures (Figure 4). Ile375 and Leu376 also occlude the substrate-binding pocket in the apo structure 2GK9, explaining why this pocket was not identified previously (Figure 4a). In the ATP pocket, the ligand interacts with the Met206 backbone NH through the pyridine nitrogen, not the amino pyrimidine moiety commonly associated with kinase hinge binding (Figure 4b). The pyridine ring also makes a hydrophobic contact with the Met206 side chain at the back of the ATP pocket (Figure 4d). The force-field-based estimates of free energy of binding (MOE, GBVI/WSA dG) suggest that the binding energy for **40** in the allosteric pocket is 4.4 kcals/mol more favorable than in the ATP pocket. There is evidence from the electron density that there is some occupancy for both ligand-binding modes in all the chains.

The **40**-PI5P4Kγ complex shows that one side of the ATP-binding pocket is formed by residues 134–141 when **40** is bound. In the apo structure 2GK9, residues 136 to 139 of this loop are missing, but they are clearly visible in chain B of the complex crystal structure, although some side chain density is poor (Figure 4c). The side chain of Phe141 of the loop contacts the isopropyl group and the pyrimidine core of **40**, and Gly136 also contacts the isopropyl group. It is likely that these interactions stabilize the loop. Chains A and C of the complex only show partial electron density for the loop. Some side chain adjustment to accommodate **40** can be observed in the ATP-binding pocket compared with apo-PI5P4Kγ. Phe141, Met206, and Lys216 have moved from their apo positions to create a pocket large enough for **40** to bind to (Figure 4d). This explains why our attempts to dock **1** and **40** to the ATP site of the apo structure were unsuccessful.

## Discussion

The optimization of **1** delivered compounds with improved molecular properties and enabled a crystal structure of a complex of PI5P4Kγ with **40** to be obtained. A retrospective analysis of the measured potencies of examples in the series fits well with the observed binding mode of **40** in the allosteric binding pocket, and on balance, the data supports this as the binding site relevant to the observed inhibition. The isopropylphenyl group is shown to fit very tightly into this binding pocket, and Table 1 shows that substituents on the phenyl ring are not tolerated in the meta and para positions (**9**, **18**, and **19**), except for F-atoms (**16** and **17**). It is not clear why compounds **20** and **21** show weak activity, but from the obtained crystal structure, binding to the ATP pocket cannot be ruled out. The ortho isopropyl group is important both for filling the lipophilic pocket formed by Leu182, Pro183, Phe185, Phe272, and Leu273 and for stabilizing the binding conformation through internal hydrophobic contact. A trifluoromethyl group at the same position is also able to fill the pocket and stabilize the conformation, but all other groups tested were, in retrospect, too small (**6**, **7**, and **8**) or too large (**25**) to fill the binding pocket or likely forced the ligand into a nonbinding conformation (**26** and **27**). The SAR around the R^2^ substituent in Table 1 shows that a 2-pyridine (**14**) is slightly favored over a phenyl substituent (**11**), and the crystal structure suggests that this is due to the interaction with Thr377. The changes shown in Table 3 are modifications to parts of the core that are mostly outside the binding pocket, so it is not surprising that these changes are well-tolerated. Only **39** is inactive, and this is likely due to the *N*-methyl group clashing with the N165 side chain.

**Figure 5:**
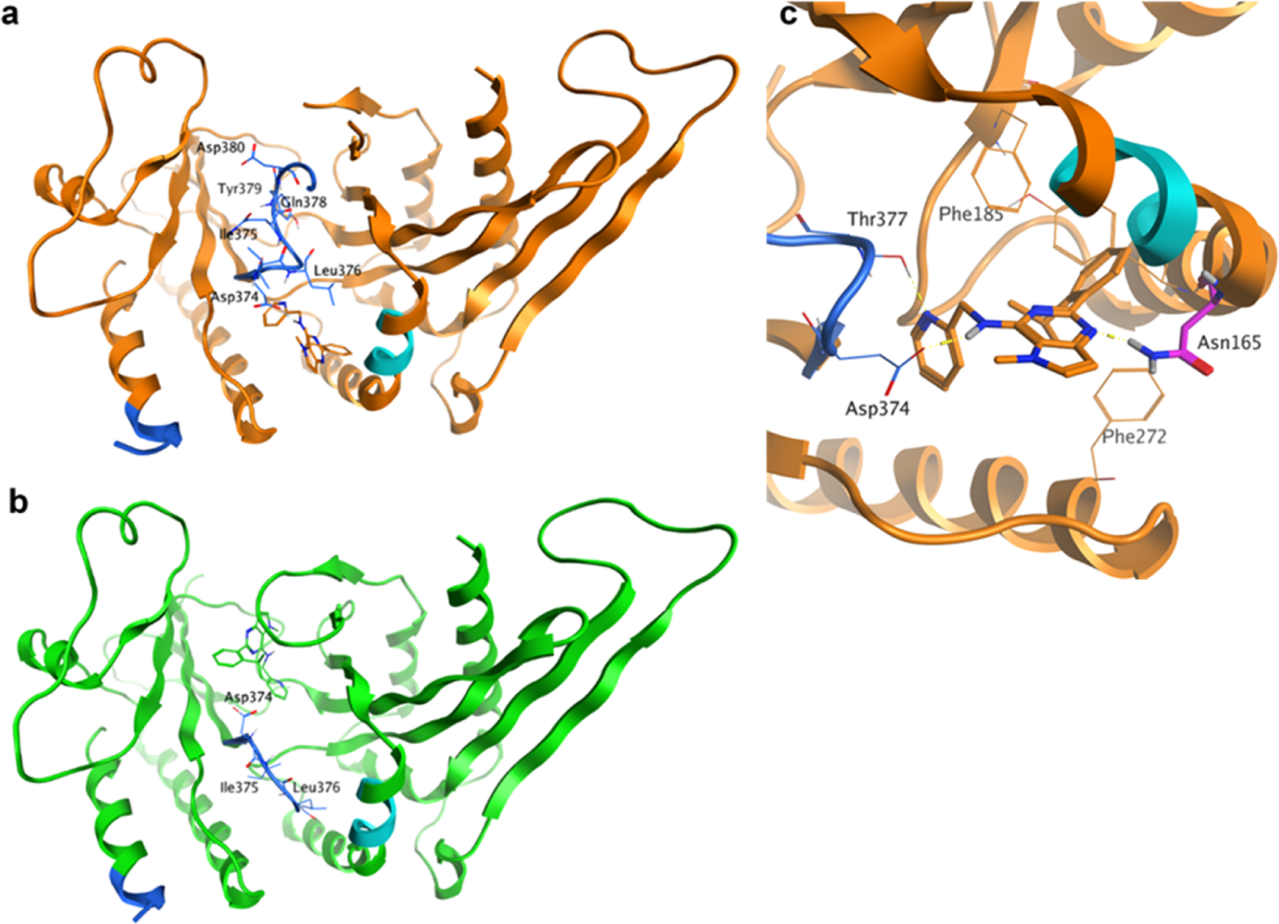
(a) Chain A in orange (allosteric binding site occupied) and (b) chain B in green (ATP site occupied) for 7QIE. HDX-MS sites identified are shown in cyan (site 1: residues 158–162) and blue (site 2: residues 373–407). Lys383A-Val403A and Thr377B-His404B, which connect the two regions of site 2, are not visible in the crystal structure. The N-terminal part of site 2 adopts different conformations in chains A and B. (c) Close-up of chain A with **40** bound highlighting the location of Asn165. The N165I mutation was found to remove the sensitivity of PI5P4Kγ to **40**.

The binding mode of **40** found in chains A, C, and D of the complex fits well with the findings from mutational studies and HDX-MS experiments by Clarke *et al.* that suggested the putative PI5P-binding site of PI5P4Kγ, not the ATP-binding site, to be the likely region of interaction^30^ of **1**. The HDX-MS method measures the rate of exchange of solvent-accessible protons from protein amides with deuterium. Changes in exchange accessibility generated by the binding of an enzyme with an inhibitor are detected by peptide MS. The regions with reduced HDX accessibility may indicate ligand-binding sites or structural changes in the protein. The main sites showing an HDX-MS effect in Clarke et al.’s study are shown in Figure 5 projected onto the PI5P4Kγ-**40** complex, where site 1 comprises residues 158–162 and site 2 comprises residues 373–407. Both sites are in close proximity to **40** in the allosteric binding mode. Residues 378–380 in particular change their environment on binding with **40**, as they move from a flexible loop conformation without electron density in the apo structure and chain B to a buried location in the alternative protein conformation of chains A, C, and D (Figure 5a,b). Clarke et al. also found that a N165I mutation removed the sensitivity of PI5P4Kγ to **1**.^30^ The NH_2_ of Asn165 makes an H-bond interaction with the pyrimidine N of **40**, therefore mutating this to an isoleucine would reduce the binding energy significantly (Figure 5c). Asn165 is unique to the PI5P4Kγ isoform; the PI5P4Kα and PI5P4Kβ isoforms have an isoleucine in this position. This explains the selectivity of **40** and analogues for the PI5P4Kγ isoform.

Given that both of the binding modes of **40** observed in the X-ray structure have an occupied ATP-binding site, it might be expected that each of these would be associated with an ATP-competitive inhibition mechanism. In the chain B structure, the ATP site is directly occupied by **40**, which would unambiguously lead to an ATP-competitive mode of inhibition. On the other hand, in chains A, C, and D, the ATP-binding site is occupied by the flexible loop. We hypothesize that the X-ray structure captures one possible conformation of this loop and that it is possible for this loop to adopt other conformations that would open up the ATP-binding site and allow **40** to remain bound in conjunction with the ATP.

Previous studies have shown that basal ATP turnover in the absence of PI5P is not inhibited by **1**, suggesting inhibitors from this chemical series can bind to the protein simultaneously with a nucleotide.^30^ In an attempt to observe this phenomenon crystallographically, we set out to determine a ternary complex with PI5P4Kγ, **40**, and the ATP derivative AMP-PNP. This resulted in a 1.95 Å crystal structure (pdb code 7QPN; Figure S3) that is highly similar to the one shown in Figures 2 and 3, but with AMP-PNP bound in a shallow pocket on the outside surface of the protein, not in the ATP pocket, as is also seen in PI5P4Kβ structure 3X04. Once again, **40** was observed to bind mainly to the allosteric pocket in this structure, with some density for **40** in the ATP pocket (occupancy of 0.35). However, we did not observe simultaneous binding of **40** to the allosteric pocket and AMP-PNP to the ATP pocket. Overall, the combined data set is most consistent with the A/C/D chain-binding mode, with support from the HDX data and with the SAR developed around this series.

In conclusion, we have optimized a series of selective small molecule PI5P4Kγ inhibitors to improve both potency and physicochemical properties and enable structural biology studies. We have presented binding constants, thermal shift data, and biochemical data, which show that, in general, this chemotype does not inhibit PI5P4Kα or β. In addition, we have shown that exemplars such as **40** are able to engage the PI5P4Kγ target in cells and that, in X-ray costructures with PI5P4Kγ, **40** can adopt two distinct modes of inhibition, with binding to a distal lipid-binding site that is 18 Å from the ATP pocket preferred. The nature of this novel mode of inhibition remains to be further elucidated and may involve PI5P competition or conformational changes in the protein that manifest through changes in the interactions with distinct protein complexes. Further studies to understand the cell biology of these inhibitors and to identify novel chemotypes with further improved potencies and physicochemical properties are underway and will be the topic of future publications.

## Experimental Section

### Chemistry

Compound **9** was purchased from Ambinter (Amb16536894) and was determined by ultra-performance liquid chromatography (UPLC) to have a purity of >95%. All the other compounds were synthesized as described below, and all the tested compounds had a purity of >95% by UPLC analysis. Compounds were synthesized according to Schemes S1–S3 as described in the Supporting Information section.

#### X-ray Crystallography and Structure Determination

Crystallography was performed by Peak Proteins Ltd. Truncated human PI5P4Kγ was expressed in *Escherichia coli* BL21(DE3) Gold using a pET28b vector. Expression was induced using 0.1 mM IPTG and the cells were cultured at 18 °C for 16 h before harvesting by centrifugation. The protein is composed of residues His32 to Ala421 with the region between and inclusive of residues 300–341 deleted. The purification of TEV-cleaved protein was by both affinity and size-exclusion (Superdex 75) chromatography. The structure of the ligand complex was generated by co-crystallization of human PI5P4Kγ in the presence of **40**. The purified protein [15.5 mg/mL in 20 mM *N*-(2-hydroxyethyl)piperazine-*N*′-ethanesulfonic acid (HEPES) pH 7.5, 150 mM NaCl, and 0.5 mM tris(2-carboxyethyl)phosphine) was incubated with 10 mM **40** (from 400 mM stock in dimethyl sulfoxide (DMSO)] overnight at 4 °C. Crystals were grown from 22% w/v Peg3350, 0.3 M ammonium tartrate, and 100 mM PCPT (sodium propionate, sodium cacodylate trihydrate, and bis–tris propane) pH 7.5 at 20 °C. Where AMP-PNP was used, the protein was first incubated with 4 mM AMP-PNP (in buffer) for 2 h and then overnight with 10 mM **40** before setting crystal trays. For X-ray data collection, they were flash-frozen and X-ray diffraction data was collected at 100 K. Diamond Light Source Synchrotron Facility, Oxford, UK, Beamlines (I03 and I24 for 7QIE and 7QPN, respectively). Data were processed using XDS and Aimless software. The phase information necessary to determine and analyze the structure was obtained by molecular replacement (PHASER, CCP4) using the previously solved structure of human PI5P4Kγ (PDB code: 2GK9) as the search model. Subsequent model building and refinement were performed according to standard protocols with software packages CCP4 and COOT. TLS refinement (REFMAC5, CCP4) has been carried out, which resulted in lower *R*-factors and higher quality of the electron density map. The ligand parameterization and the generation of the corresponding library files were carried out with ACEDRG (CCP4). The Ramachandran plots of the final models show 91.3 and 92.3% of all the residues in the most favored regions and 7.0 and 5.0% in the additionally allowed regions, for 7QIE and 7QPN, respectively. Statistics of the final structure and the refinement process are listed in Table S5.

#### Computational Modeling

The Maestro QM tautomer and conformer predictor (release 2019-3, Schrodinger, https://www.schrodinger.com/) was used to predict the 5 lowest energy conformers of **40**. The predictor generates conformers using macromodel, and then performs density functional theory geometry optimizations on the structures, using the B3LYP-D3/LACVP** level of theory. The structures were then ranked using optimization energies at the M06-2X/cc-pVTZ(-f) theory level in solution (water/PBF) calculated using the geometries from the previous step. The root mean square deviation (RMSD) of the lowest energy conformer with the ligand from chain A of the minimized crystal structure (Maestro Protein Preparation, default settings) was then calculated using the maximum common structure superposition algorithm in Maestro.

The free energy of binding was calculated using the Dock panel in MOE (release 2019.0101, Chemical Computing Group, www.chemcomp.com), using the crystal structure placement, with induced fit refinement guided by the GBVI/WSA dG scoring function. E_refine for the two binding modes of **40** was compared.

Marvin was used for *c* log *P* calculations using the consensus method, (Marvin 20.15, ChemAxon https://www.chemaxon.com).

#### Biochemical Assays

Recombinant mutant PI5P4Kγ+ was prepared essentially as described previously.^25^ The protein from *PIP4K2C* (UniGene 6280511), genetically modified to have a specific activity close to that of the active PI5P4Kα isoform^25^ and cloned into the expression vector pGEX6P (Cytiva), was expressed and purified from *E. coli* BL21(DE3). Cultures were induced with 0.4 mM IPTG and probe sonicated in the presence of protease inhibitors. The GST fusion protein of PI5P4Kγ+ was harvested by binding to glutathione sepharose beads (Cytiva) and cleaved *in situ* with 50U of PreScission protease (Cytiva) for 4 h at 4 °C. The cleaved protein was further purified by size-exclusion chromatography (AKTA Pure, Cytiva). The protein purity was confirmed by sodium dodecyl sulphate–polyacrylamide gel electrophoresis, and the concentration was determined by colorimetric assay (Bio-Rad).

PI5P4Kγ+ activity in the presence of inhibitor compounds was determined by the ADP-Glo assay (Promega). The assay was performed in a 384-well plate (Greiner 784201) with serial dilutions of the test compound in 18 μL of the reaction mix (20 μM di-C8 PI5P, 10 μM ATP, 33 mM HEPES pH 7.4, 0.1% 3-[(3-cholamidopropyl)dimethylammonio]-1-propanesulfonate (CHAPS), 20 mM MgCl_2_, and 16.7 μM (ethylene glycol-bis(β-aminoethyl ether)-N,N,N′,N′-tetraacetic acid) (EGTA)). The plate was incubated for 60 min at room temperature after the addition of purified PI5P4Kγ+ (150 ng/well) and prior to the transfer of 4 μL of the reaction mix into a second plate (Greiner 784904) containing a 4 μL of ADP-Glo reagent for a further 40 min of incubation. After incubation with 8 μL of the kinase detection reagent for 30 min, plate luminescence was read (Pherastar FSX, BMG Labtech).

The binding of compounds to PI5P4Kγ in intact cells was assessed using an InCell Pulse thermal stabilisation assay (DiscoverX). Hek293 cells stably expressing ePL-tagged PI5P4Kγ were incubated with 25 nL of the test compound in 100% DMSO in a black skirted polymerase chain reaction (PCR) plate for 60 min at 38 °C. After incubation for 3 min at 46 °C, followed by cooling for 3 min at room temperature, 12 μL of the EA-3 reagent (prepared as per the manufacture’s guidelines) was added to each well. The plate was then incubated for 60 min in the dark prior to luminescence reading using a Pherastar FSX plate reader (BMG Labtech).

#### Data Analysis

Statistical analysis was performed using nonparametric testing in Prism 8 (GraphPad). Activity pIC_50_ values and *in vivo* binding pEC_50_ values were estimated using a 4-parameter fit (Dotmatics).

### Thermal Shift Assay

The thermal shift assay was performed with an Applied Biosystem StepOne Real-Time PCR System in 96-well plates sealed with optically clear lids. The fluorescent dye Sypro Orange was used to report on protein unfolding. The final concentration of the PI5P4K protein was 4 μM, the ligand was at 63 μM, in a final volume of 20 μL. A 5000× stock solution of Sypro Orange was used, at a final concentration of 5×. The buffer was 50 mM HEPES pH 7.4, 5 mM MgCl_2_, and 100 mM NaCl. The plates were heated from 25 to 90 °C at a rate of 0.5 °C/min. The thermal melting point was calculated for each well by monitoring the minimum point of the negative derivative of the fluorescence unfolding curves. Thermal shifts were calculated by comparison to control wells with no compound (5% DMSO). At least two chemical replicates of each ligand were performed, with control compounds used to check for consistency between plates.

## Supporting Information Available

Supplementary tables and figures and further experimental details including ADMET, chemical synthesis with schemes, selected nuclear magnetic resonance and HPLC spectra, and molecular formula strings (PDF)
SMILES data for Table 1 (CSV)
SMILES data for Table 2 (CSV)
SMILES data for Table 3 (CSV)
SMILES data for Table S1 (CSV)
SMILES data for Table S2 (CSV)
SMILES data for Table S3 (CSV)
SMILES data for Table S4 (CSV)

## Notes

Authors will release the atomic coordinates upon article publication. 7QIE: 2.4 Å structure of 40 bound to PI5P4Kγ and 7QPN: 1.95 Å structure of AMP-PNP and 40 bound to PI5P4Kγ.

## Abbreviations

ATP: adenosine triphosphate
LE: ligand efficiency
mTOR: mechanistic target of rapamycin
PI5P4K: phosphatidylinositol 5-phosphate 4-kinase
SAR: structure–activity relationships
WT: wild-type
