# Supplementary material for: Development of Selective Phosphatidylinositol 5-Phosphate 4-Kinase γ Inhibitors with a Non-ATP-competitive, Allosteric Binding Mode
Source: J Med Chem. 2022 Feb 11;65(4):3359–70. doi: 10.1021/acs.jmedchem.1c01819 (PMC9097471; doi:10.1021/acs.jmedchem.1c01819)
Supplement: Supplementary file 2 — jm1c01819_si_002.pdf [file jm1c01819_si_002.pdf]

# Supporting Information

# Development of Selective Phosphatidylinositol 5-Phosphate 4-Kinase γ (PI5P4Kγ) Inhibitors with a Non-ATP-competitive, Allosteric Binding Mode

Helen K. Boffey,[1‡] Timothy P. C. Rooney,[1‡] Henriette M. G. Willems,[1‡] Simon Edwards,[1‡] Christopher Green,[2] Tina Howard,[3] Derek Ogg,[3] Tamara Romero,[1] Duncan E. Scott,[1] David Winpenny,[1] James Duce,[1] John Skidmore,[1] Jonathan H. Clarke[1] and Stephen P Andrews[1]*

**Addresses**

[1]Jonathan Clarke, Helen Boffey, Simon Edwards, Tamara Romero, Timothy Rooney, Duncan Scott, Henriette Willems, David Winpenny, James Duce, John Skidmore and Stephen P Andrews: The ALBORADA Drug Discovery Institute, University of Cambridge, Island Research Building, Cambridge Biomedical Campus, Hills Road, Cambridge, CB2 0AH, United Kingdom

[2]Christopher Green: UK Dementia Research Institute, University of Cambridge, Island Research Building, Cambridge Biomedical Campus, Hills Road, Cambridge, CB2 0AH, United Kingdom

[3]Tina Howard, Derek Ogg: Peak Proteins, Alderley Park, Macclesfield, Cheshire, SK10 4TG, United Kingdom

**Corresponding Author**

*E-mail: spa26@cam.ac.uk

‡These authors contributed equally.

## Table of Contents


Supplementary Tables

Supplementary Figures

Experimental Section

- ADMET method details
- Chemistry General Experimental
- Schemes S1-S3
- Synthesis of compounds

NMR Spectra

HPLC Spectra

References

**Table S1.** Comparison of PI5P4Kγ+ activity data generated in the ADP-Glo assays reported here, versus publicly available PI5P4Kγ data from kinase panels for six known kinase inhibitors.

| **Chemical Identifiers** | | | **New Data[a]** | | | **public data for PI5P4Kγ (sourced from: DrugTargetCommons, ChEMBL, PUBMED)** | |
|---|---|---|---|---|---|---|---|
| **Preferred Name** | **SMILES** | **ChEMBL ID** | **PI5P4Kα pIC$_{50}$** | **PI5P4Kβ pIC$_{50}$** | **PI5P4Kγ+ pIC$_{50}$** | **pK$_D$** | **reference** |
| ML197 | Cc1nc(cs1)CNc1ncnc2ccc(cc12)-c1ccc2c(c1)OCO2 | CHEMBL1435542 | <4.3 | ND | 5.4 | 6.6 | Rosenthal AS, Tanega C, Shen M, Mott BT, Bougie JM, Nguyen DT, Misteli T, Auld DS, Maloney DJ, Thomas CJ, Potent and selective small molecule inhibitors of specific isoforms of Cdc2-like kinases (Clk) and dual specificity tyrosine-phosphorylation-regulated kinases (Dyrk), Bioorg. Med. Chem. Lett., 2011, 21, 10, 3152, 3158, 10.1016/j.bmcl.2011.02.114 |
| ML106 | CN(Cc1csc(n1)C)c1ncnc2ccc(cc12)-c1ccc2c(c1)OCO2 | CHEMBL1474834 | <4.3 | ND | 5.0 | 6.4 | Rosenthal AS, Tanega C, Shen M, Mott BT, Bougie JM, Nguyen DT, Misteli T, Auld DS, Maloney DJ, Thomas CJ, Potent and selective small molecule inhibitors of specific isoforms of Cdc2-like kinases (Clk) and dual specificity tyrosine-phosphorylation-regulated kinases (Dyrk), Bioorg. Med. Chem. Lett., 2011, 21, 10, 3152, 3158, 10.1016/j.bmcl.2011.02.114 |
| Foretinib | COc1cc2c(ccnc2cc1OCCCN1CCOCC1)Oc1ccc(cc1F)NC(=O)C1(CC1)C(=O)Nc1ccc(cc1)F | CHEMBL1230609 | <4.3 | ND | 6.2 | 6.8 | Davis MI, Hunt JP, Herrgard S, Ciceri P, Wodicka LM, Pallares G, Hocker M, Treiber DK, Zarrinkar PP, Comprehensive analysis of kinase inhibitor selectivity, Nat. Biotechnol., 2011, 29, 11, 1046, 1051, 10.1038/nbt.1990 |
| BI-2536 | CC[C@H]1N(C2CCCC2)c2nc(ncc2N(C)C1=O)Nc1ccc(cc1OC)C(=O)NC1CCN(C)CC1 | CHEMBL513909 | *4.6 | ND | 6.0 | 6.3 | Davis MI, Hunt JP, Herrgard S, Ciceri P, Wodicka LM, Pallares G, Hocker M, Treiber DK, Zarrinkar PP, Comprehensive analysis of kinase inhibitor selectivity, Nat. Biotechnol., 2011, 29, 11, 1046, 1051, 10.1038/nbt.1990 |
| Pazopanib | CN(c1ccc2c(n(nc2c1)C)C)c1ccnc(n1)Nc1ccc(c(c1)S(=O)(=O)N)C | CHEMBL477772 | *4.8 | ND | 6.0 | 6.6 | Karaman MW, Herrgard S, Treiber DK, Gallant P, Atteridge CE, Campbell BT, Chan KW, Ciceri P, Davis MI, Edeen PT, Faraoni R, Floyd M, Hunt JP, Lockhart DJ, Milanov ZV, Morrison MJ, Pallares G, Patel HK, Pritchard S, Wodicka LM, Zarrinkar PP, A quantitative analysis of kinase inhibitor selectivity., Nat. Biotechnol., 2008, 26, 1, 127, 132, 10.1038/nbt1358 |
| Palbociclib | CC(=O)C1=C(C)c2cnc(nc2N(C2CCCC2)C1=O)Nc1ccc(cn1)N1CCNCC1 | CHEMBL189963 | <4.3 | <4.6 | <4.3 | 5.8 | Klaeger S, Heinzlmeir S and Wilhelm M et al., The target landscape of clinical kinase drugs, Science, 2017, 358, ean4368, 10.1126/science.aan4368 |

[a]pIC$_{50}$s determined in the ADP-Glo assays described herein

**Table S2.** Reported PI5P4K activity of compounds **1-5**, and comparison with data generated in the assays described in this article (where available).

| **Compound** | **Previously published kinase inhibition data** | | | | **New data** | | | |
|---|---|---|---|---|---|---|---|---|
| | **PI5P4Kα activity (assay)** | **PI5P4Kβ activity (assay)** | **PI5P4Kγ activity (assay)** | **ref** | **PI5P4Kα $IC_{50}$ (µM)[a]** | **PI5P4Kβ $IC_{50}$ (µM)[a]** | **PI5P4Kγ+ $IC_{50}$ (µM)[a]** | **PI5P4Kγ-WT target engagement $IC_{50}$ (µM)[b]** |
| 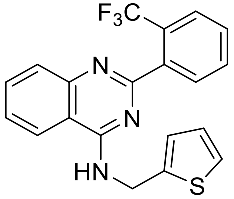 NIH-12848 (**1**) | >100 µM ($^{32}$P-ATP/PI5P incorporation) | >100 µM ($^{32}$P-ATP/PI5P incorporation)[c] | 2-3 µM ($^{32}$P-ATP/PI5P incorporation) | 1 | >50 | >25 | 0.79 | 2.5 |
| 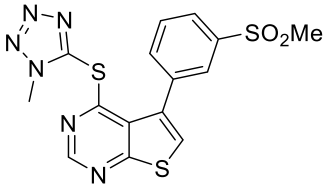 NCT-504 (**2**) | <35% inh @ 10 µM (KINONMEscan) | <65% inh @ 10 µM (KINONMEscan) | 16 µM ($^{32}$P-ATP/PI5P incorporation) | 2 | 2.0 | >25 | >50 | >50 |
| 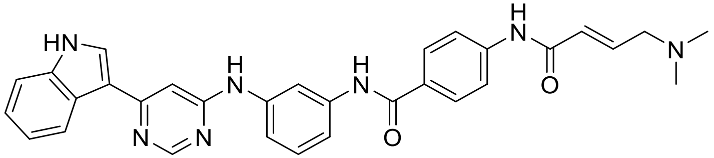 THZ-P1-2 (**3**) | 0.95 µM (bioluminescence assay) | 5.9 µM (FP assay) | 91% inh. @ 1 µM (KINONMEscan) | 3 | 0.13 | 1.0 | 0.13 | 1.3 |
| 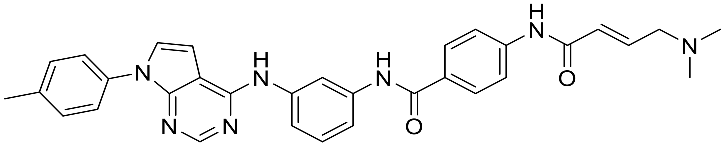 "compound 30" (**4**) | 1.3 µM (bioluminescence assay) | 9.9 µM (FP assay) | 22% inh. @ 1 µM (KINONMEscan) | 4 | ND | ND | ND | ND |
| 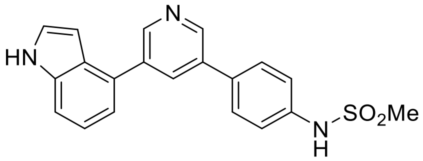 "compound 13" (**5**) | 2 µM (bioluminescence assay) | 22 µM (FP assay) | 100% inh. @ 1 µM (KINONMEscan) 0.0034 µM (Ambit $K_D$) | 3 | ND | ND | ND | ND |

[a]determined in the ADP-Glo assays described herein; [b]determined in the INCell Pulse assay described herein; [c]showed 'small but significant stimulation'.

**Table S3**: ADP-Glo $pIC_{50}$ for compound against PI5P4Kγ+. Thermal shift ($\Delta T_m$) values for compounds against PI5P4Kγ-WT with compound concentration at 63 µM

| | PI5P4Kγ+ $pIC_{50}$ | PI5P4Kγ-WT $\Delta T_m$ / °C |
|---|---|---|
| **1** | 6.1 ± 0.3 | 4.7 ± 0.8 |
| **6** | <4.3 ± 0.0 | -1.1 ± 0.3 |
| **7** | <4.5 ± 0.4 | 2.7 ± 0.0 |
| **8** | <4.3 ± 0.0 | 0.1 ± 0.0 |
| **9** | <4.3 ± 0.0 | -1.6 ± 0.4 |
| **10** | <4.3 ± 0.0 | -0.8 ± 0.0 |
| **11** | 5.2 ± 1.0 | 4.6 ± 0.0 |
| **12** | <4.3 ± 0.0 | 1.4 ± 0.4 |
| **13** | 5.5 ± 0.2 | 6.1 ± 0.0 |
| **14** | 5.5 ± 0.5 | 6.3 ± 0.6 |
| **15** | <4.3 ± 0.0 | -1.9 ± 0.0 |
| **18** | <4.3 ± 0.0 | 1.3 ± 0.2 |
| **19** | <4.3 ± 0.0 | 1.7 ± 0.7 |
| **20** | 5.1 ± 0.2 | 5.1 ± 0.0 |
| **21** | 5.1 ± 0.1 | 5.1 ± 0.0 |
| **22** | 6.5 ± 0.3 | 7.8 ± 0.7 |
| **24** | 5.3 ± 0.4 | 4.9 ± 0.7 |
| **25** | 5 ± 0.2 | 4.4 ± 0.7 |
| **26** | <4.3 ± 0.0 | 2.9 ± 0.4 |
| **27** | <4.3 ± 0.0 | 2.7 ± 0.4 |
| **29** | <4.4 ± 0.3 | 0.2 ± 0.4 |
| **31** | 6.2 ± 0.3 | 7.0 ± 0.0 |
| **34** | 5.4 ± 0.2 | 4.4 ± 0.0 |
| **35** | 5 ± 0.3 | 3.9 ± 0.0 |
| **36** | 5.6 ± 0.6 | 4.4 ± 0.0 |
| **38** | 6.1 ± 0.2 | 5.5 ± 0.0 |
| **40** | 6.2 ± 0.3 | 6.9 ± 0.0 |
| **41** | 5.6 ± 0.3 | 4.9 ± 0.0 |
| **42** | 6.5 ± 0.3 | 6.8 ± 0.4 |

**Table S4:** Thermal shift ($\Delta T_m$) profile of compounds **1**, **14** and **22** against α, β, γ-WT and γ+ isoforms of PI5P4K. Compound concentration was 63 µM.

| | PI5P4Kα $\Delta T_m$ / °C | PI5P4Kβ $\Delta T_m$ / °C | PI5P4Kγ-WT $\Delta T_m$ / °C | PI5P4Kγ+ $\Delta T_m$ / °C |
|---|---|---|---|---|
| **1** | -0.2 ± 0.4 | 0.6 ± 0.3 | 4.7 ± 0.8 | 7.4 ± 0.6 |
| **14** | -0.2 ± 0.4 | 0.4 ± 0.2 | 6.3 ± 0.6 | n.d. |
| **22** | n.d. | n.d. | 7.8 ± 0.8 | 12.1 ± 0.5 |

**Table S5:** Data collection and refinement statistics for X-ray crystal structures of PI5P4Kγ bound to **40**.

| PDB ID | **7QIE** | **7QPN** |
|---|---|---|
| Protein/Ligand | PI5P4Kγ/**40** | PI5P4Kγ/**40**/AMP-PNP |
| Wavelength [Å] | 0.976250 | 0.9999 |
| Space group | P 21 | P 21 |
| a; b; c; [Å] | 49.41; 114.79; 146.9 | 47.92; 65.62; 117.12 |
| α; β; γ; [◦] | 90.0; 95.1; 90.0 | 90.0; 93.16; 90.0 |
| Resolution [Å] | 146.17-2.39 (2.46-2.39)[a] | 116.94-1.95 (2.14-1.95) |
| Unique reflections | 62636 (18607)[2] | 41286 |
| Multiplicity | 3.4 (3.3)[2] | 5.5 (5.1)[2] |
| Completeness [%] | 97.8 (99.4) | 91.7 (56.5) |
| Rsym [%] | 10 (271)[2] | 11 (174)[2] |
| Rmeas [%] | 12 (220)[2] | 13 (199)[2] |
| Mean(I)/sd | 4.7 (0.4)[2] | 8.9 (1.1)[2] |
| CC(1/2) | 0.998 (0.359) | 0.996 (0.557) |
| Number of reflections (free) | 34407 (1799) | 37666 (1877) |
| $R_{cryst}$ [%] | 21.4 | 19.2 |
| $R_{free}$[%] | 26.6 | 26.2 |
| Protein | 9762 | 5132 |
| Water | 198 | 210 |
| Ligand | 108 | 116 |
| Deviation from ideal geometry: | | |
| Bond lengths [Å] | 0.01 | 0.01 |
| Bond angles [◦] | 1.60 | 1.60 |

[a]values in parenthesis refer to the highest resolution bin.

**Table S6:** $K_D$ values for **1** and **40**.[a]

| **Compound** | **PI5P4Kβ $K_D$ (nM)** | **PI5P4Kγ $K_D$ (nM)** |
|---|---|---|
| **1** | >30,000 | 4000 |
| **40** | >30,000 | 68 |

[a] Data were generated at Eurofins Discovery using DiscoverX KINOMEscan™ technology. Streptavidin-coated magnetic beads were treated with biotinylated small molecule ligands for 30 minutes at room temperature to generate affinity resins for kinase assays. The liganded beads were blocked with excess biotin and washed with blocking buffer (SeaBlock (Pierce), 1% BSA, 0.05% Tween 20, 1 mM DTT) to remove unbound ligand and to reduce non-specific binding. Binding reactions were assembled by combining kinases, liganded affinity beads, and test compounds in 1x binding buffer (20% SeaBlock, 0.17x PBS, 0.05% Tween 20, 6 mM DTT). Test compounds were prepared as 111X stocks in 100% DMSO. Kds were determined using an 11-point 3-fold compound dilution series with three DMSO control points. All compounds for Kd measurements are distributed by acoustic transfer (non-contact dispensing) in 100% DMSO. The compounds were then diluted directly into the assays such that the final concentration of DMSO was 0.9%. All reactions performed in polypropylene 384-well plate. Each was a final volume of 0.02 ml. The assay plates were incubated at room temperature with shaking for 1 hour and the affinity beads were washed with wash buffer (1x PBS, 0.05% Tween 20). The beads were then re-suspended in elution buffer (1x PBS, 0.05% Tween 20, 0.5 µM nonbiotinylated affinity ligand) and incubated at room temperature with shaking for 30 minutes. The kinase concentration in the eluates was measured by qPCR.

**Table S7.** PI5P4Kα adapta assay results for compounds **1** and **40**.[a]

| **Compound** | **PI5P4Kα adapta $IC_{50}$ (nM)** |
|---|---|
| **1** | >30,000 |
| **40** | >30,000 |

[a] The experiments were run by Thermo Fisher. This assay is a TR-FRET based assay using an Eu-anti-ADP antibody, the conditions are: the 2X PIP4Kα/PI(5)P mixture is prepared in 50 mM HEPES pH 7.5, 0.1% CHAPS, 1 mM EGTA, 4 mM MgCl2. The final 10 µL Kinase Reaction consists of 1.5 - 6 ng PIP4Kα and 50 µM PI(5)P in 32.5 mM HEPES pH 7.5, 0.05% CHAPS, 0.5 mM EGTA, 2 mM MgCl2. After the 1 hour Kinase Reaction incubation, 5 µL of Detection Mix is added. The Detection mix consists of EDTA (30mM), Eu-anti-ADP antibody (6 nM) and ADP tracer in TR-FRET buffer. The detection mix contains the EC60 concentration of tracer for 5-150 mM ATP.

**Table S8.** Kinase selectivity screening for **40** at 10 μM against a general kinase panel of 140 targets in radiometric filter binding assay using 33P-g-ATPat the MRC PPU International Centre for Kinase Profiling, University of Dundee.

| **Kinase** | **% activity remaining** | **s.d.** |
|---|---|---|
| MKK1 | **92** | 7 |
| MKK2 | **115** | 1 |
| MKK6 | **90** | 2 |
| ERK1 | **111** | 11 |
| ERK2 | **98** | 7 |
| ERK5 | **138** | 13 |
| JNK1 | **86** | 12 |
| JNK2 | **110** | 14 |
| JNK3 | **97** | 4 |
| p38a MAPK | **100** | 6 |
| p38b MAPK | **94** | 11 |
| p38g MAPK | **105** | 7 |
| p38d MAPK | **117** | 3 |
| ERK8 | **109** | 1 |
| RSK1 | **99** | 2 |
| RSK2 | **106** | 8 |
| PDK1 | **103** | 4 |
| PKBa | **103** | 4 |
| PKBb | **75** | 1 |
| SGK1 | **98** | 7 |
| S6K1 | **95** | 11 |
| PKA | **109** | 9 |
| ROCK 2 | **97** | 14 |
| PRK2 | **83** | 14 |
| PKCa | **118** | 9 |
| PKCγ | **77** | 4 |
| PKCz | **111** | 1 |
| PKD1 | **109** | 10 |
| STK33 | **99** | 1 |
| MSK1 | **95** | 15 |
| MNK1 | **104** | 8 |
| MNK2 | **117** | 7 |

| | | |
|---|---|---|
| MAPKAP-K2 | **123** | 7 |
| MAPKAP-K3 | **102** | 18 |
| PRAK | **98** | 1 |
| CAMKKb | **99** | 11 |
| CAMK1 | **97** | 7 |
| SmMLCK | **108** | 12 |
| PHK | **107** | 11 |
| DAPK1 | **105** | 2 |
| CHK1 | **96** | 5 |
| CHK2 | **110** | 0 |
| GSK3b | **93** | 14 |
| CDK2-Cyclin A | **103** | 3 |
| CDK9-Cyclin T1 | **108** | 8 |
| PLK1 | **97** | 1 |
| Aurora A | **94** | 10 |
| Aurora B | **113** | 14 |
| TLK1 | **115** | 9 |
| LKB1 | **94** | 6 |
| AMPK (hum) | **88** | 1 |
| MARK1 | **88** | 6 |
| MARK2 | **101** | 1 |
| MARK3 | **101** | 6 |
| MARK4 | **105** | 2 |
| BRSK1 | **115** | 10 |
| BRSK2 | **83** | 3 |
| MELK | **95** | 8 |
| NUAK1 | **88** | 4 |
| SIK2 | **100** | 5 |
| SIK3 | **111** | 11 |
| TSSK1 | **96** | 9 |
| CK1γ2 | **97** | 1 |
| CK1δ | **100** | 3 |
| CK2 | **90** | 15 |
| TTBK1 | **117** | 3 |
| TTBK2 | **114** | 8 |
| DYRK1A | **101** | 12 |
| DYRK2 | **106** | 1 |

| | | |
|---|---|---|
| DYRK3 | **128** | 13 |
| NEK2a | **103** | 4 |
| NEK6 | **120** | 2 |
| IKKb | **100** | 13 |
| IKKe | **98** | 11 |
| TBK1 | **93** | 0 |
| PIM1 | **102** | 3 |
| PIM2 | **110** | 1 |
| PIM3 | **87** | 3 |
| SRPK1 | **93** | 5 |
| EF2K | **98** | 6 |
| EIF2AK3 | **103** | 9 |
| HIPK1 | **104** | 11 |
| HIPK2 | **114** | 13 |
| HIPK3 | **92** | 10 |
| CLK2 | **94** | 6 |
| PAK2 | **45** | 8 |
| PAK4 | **95** | 1 |
| PAK5 | **120** | 9 |
| PAK6 | **95** | 2 |
| MST2 | **115** | 7 |
| MST3 | **110** | 13 |
| MST4 | **110** | 17 |
| GCK | **93** | 6 |
| MAP4K3 | **91** | 6 |
| MAP4K5 | **112** | 1 |
| MINK1 | **95** | 10 |
| MEKK1 | **68** | 5 |
| MLK1 | **89** | 10 |
| MLK3 | **90** | 2 |
| TESK1 | **114** | 1 |
| TAO1 | **115** | 0 |
| ASK1 | **116** | 0 |
| TAK1 | **110** | 4 |
| IRAK1 | **114** | 3 |
| IRAK4 | **114** | 5 |
| RIPK2 | **92** | 10 |

| | | |
|---|---|---|
| OSR1 | **120** | 14 |
| TTK | **94** | 7 |
| MPSK1 | **106** | 14 |
| WNK1 | **112** | 6 |
| ULK1 | **111** | 3 |
| ULK2 | **91** | 0 |
| TGFBR1 | **91** | 4 |
| Src | **135** | 13 |
| Lck | **102** | 7 |
| CSK | **94** | 4 |
| YES1 | **115** | 6 |
| ABL | **108** | 10 |
| BTK | **111** | 2 |
| JAK3 | **97** | 3 |
| SYK | **91** | 2 |
| ZAP70 | **95** | 5 |
| TIE2 | **89** | 3 |
| BRK | **112** | 2 |
| EPH-A2 | **104** | 12 |
| EPH-A4 | **96** | 8 |
| EPH-B1 | **95** | 9 |
| EPH-B2 | **78** | 1 |
| EPH-B3 | **105** | 13 |
| EPH-B4 | **120** | 6 |
| FGF-R1 | **92** | 3 |
| HER4 | **112** | 2 |
| IGF-1R | **107** | 7 |
| IR | **119** | 13 |
| IRR | **99** | 12 |
| TrkA | **100** | 1 |
| DDR2 | **90** | 8 |
| VEG-FR | **125** | 14 |
| PDGFRA | **100** | 1 |
| PINK | **125** | 10 |

**Table S9.** Lipid kinase selectivity screening for **40** at 10 μM against a kinase panel of 15 protein kinase targets using ADP-GloTM assay at the MRC PPU International Centre for Kinase Profiling, University of Dundee.

| **Kinase** | **% activity remaining** | **s.d.** |
|---|---|---|
| PI3Kalpha | **91.4** | 2.4 |
| PI3K beta | **111.2** | 1.2 |
| PI3K gamma | **102.9** | 2.3 |
| Choline Kinase alpha | **101.6** | 3.2 |
| Choline Kinase beta | **97.3** | 12.0 |
| PIP5K2a | **100.3** | 0.7 |
| PI3Ka E524K +P85 | **102.0** | 10.7 |
| PI3KA E545K +P85 | **91.7** | 0.9 |
| PI4K2a | **93.5** | 6.5 |
| SPHK1 | **91.8** | 2.4 |
| SPHK2 | **83.8** | 6.8 |
| PIK4Ca | **81.1** | 3.0 |
| DGK beta | **102.0** | 3.4 |
| DGK gamma | **99.4** | 4.6 |
| DGK zeta | **106.2** | 0.4 |

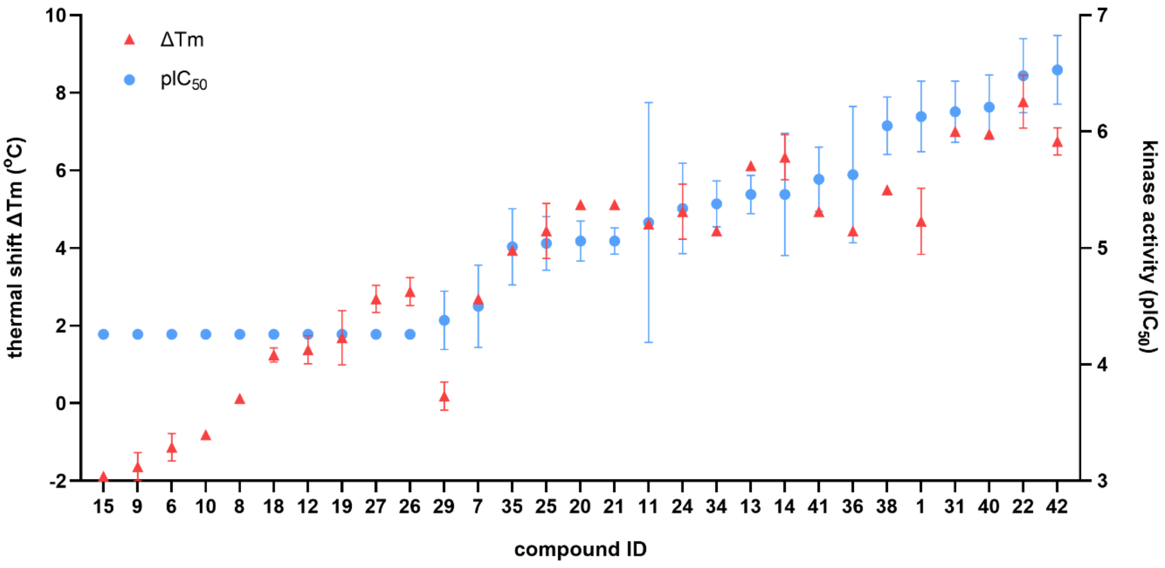


**Figure S1**: A comparison of compound $pIC_{50}$ for PI5P4Kγ+ (red triangle, determined by ADP-Glo) to ΔTm for PI5P4Kγ-WT (blue circle, determined by thermal shift assay). Data are shown as mean value ± standard error.

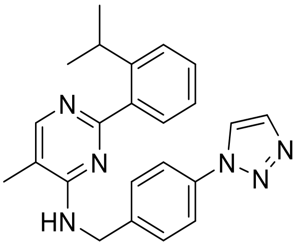


**Figure S2**: Structure of ML323.[5]

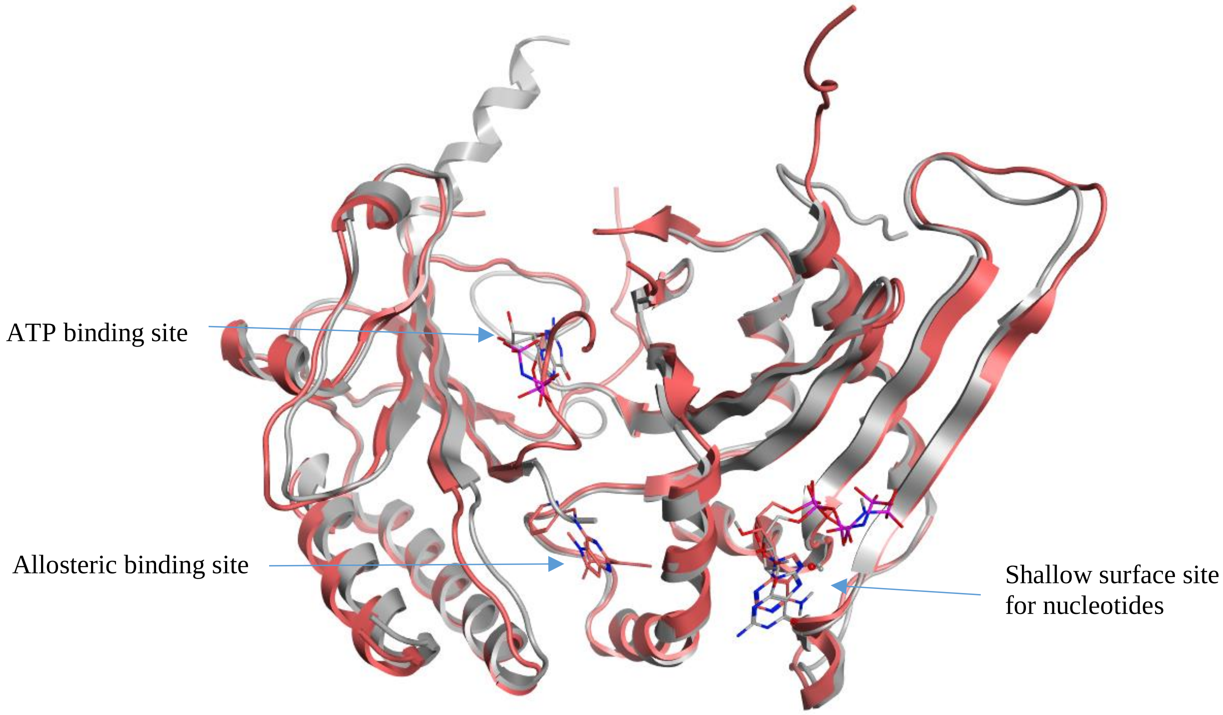


**Figure S3**. PI5P4Kγ/**40**/AMP-PNP (pdb 7QPN) in rose superposed onto PI5P4Kβ structure 3X04 chain A in grey. One GMP-PNP molecule binds in the ATP site of 3X04 binds and another one in a similar position to the AMP-PNP ligand of PI5P4Kγ/**40**/AMP-PNP on the surface of the protein (bottom right).

# Supporting Experimental:

**ADMET experimental**

**Microsomal stability** analysis was performed by Charles River Laboratories Inc. (ADME-SOP-84). Briefly, test compounds in DMSO were incubated at a concentration of 1 µM (0.25% DMSO final) with mouse hepatic microsomes (0.5 mg protein/mL) in the presence of NADPH (1 mM) at 37 °C. Aliquots were taken at time intervals and analysed by mass spectrometry for compound remaining, allowing the determination of the half-life for the compound.

**Plasma protein binding** analysis was performed by Charles River Laboratories Inc. (ADME-SOP-90). Briefly, compounds in DMSO (10 µM, 0.5% DMSO final) were added to mouse plasma and dialysed against buffer for 4 hours at 37 °C. The compound concentration in each of the plasma and buffer compartments was determined by mass spectrometry and used to calculate the percentage compound bound.

**Permeability:** bi-directional Caco-2 cell permeability was performed by Charles River Laboratories Inc. (ADME-SOP-49). Briefly, compounds were administered at 10 µM (1% DMSO final) to the apical or basolateral side of a polarised Caco-2 cell monolayer, then incubated at 37 °C for 60 minutes before appearance on the opposite side of the monolayer was determined by mass spectrometry. The efflux ratio (ER) is calculated from the ratio of B-A to A-B permeabilities.

**Kinetic solubility** analysis was performed by Charles River Laboratories Inc. (ADME-SOP-01). Compound in DMSO at 10 mM was diluted to 200 µM in both DMSO and buffer (0.1 M PBS, pH 7.4, 2% DMSO final), and an aliquot of the 200 µM DMSO solution was diluted to 10 µM, and all dilutions were equilibrated for 2 hours. Compound concentration in the PBS filtrate was determined by LC-UV and comparing to the DMSO dilutions as calibration standards.

**mchrom_LogD:** The chromatographic LogD value was determined from the chromatographic hydrophobicity index (CHI) value using the equation $mchromLogD_{7.4} = 0.0857\ CHI_{7.4} - 2$( ref 6)[6] The CHI value of an individual compound was measured on a Waters Aquity UPLC system, XSelect HSS C18 5 µm 4.6x150 mm HPLC column, 5-100% gradient of MeCN in 50mM NH4OAc in $H_2O$ adjusted to pH 7.4. Retention times of standards with known CHI were used to establish the linear regression expression for use on the test compounds[7].

**Chemistry General Experimental:**

Compound **9** was purchased from Ambinter (Amb16536894) and was determined by UPLC to have purity >95%. All other compounds were synthesised as described below, and all tested compounds have purity >95% by UPLC

analysis. Reagents and solvents were of commercially available reagent grade quality and used without further purification. Reactions requiring anhydrous conditions were carried out in oven dried glassware under an atmosphere of $N_2$. Reactions were monitored by thin-layer chromatography on silica gel 60 $F_{254}$ aluminium or glass supported sheets, or by liquid chromatography-mass spectrometry (LCMS). Flash column chromatography was carried out on a Biotage Isolera One system using normal phase ($SiO_2$) cartridges. Compounds were loaded in solution or adsorbed onto Celite® 545, and eluted using a linear gradient of the specified solvents. Purification by C18 reverse phase HPLC was carried using an Agilent 1260 Infinity machine and a Waters XBridge BEH C18 OBD column (130 Å, 5 µm, 30 mm × 100 mm) with a linear gradient of $H_2O$ (with 0.1% $NH_3$) and MeCN (with 0.1% $NH_3$). LCMS analysis was performed on a Waters Aquity HClass UPLC system with an Aquity QDa for mass detection. High-resolution mass spectra (HRMS) were measured on a Waters Vion IMS QTof spectrometer. NMR spectra were recorded on a Bruker Advance III ($^1H$ = 300 MHz, $^{19}F$ = 282 MHz, $^{13}C$ = 75 MHz) spectrometer using the requisite solvent as a reference for internal deuterium lock. The chemical shift data for each signal are given as δ chemical shift (multiplicity, *J* values in Hz, integration) in units of parts per million (ppm) relative to tetramethylsilane (TMS) where δH (TMS) = 0.00 ppm. The multiplicity of each signal is indicated by: s (singlet), d (doublet), t (triplet), q (quartet), quin (quintet), hept (heptet) or m (multiplet). Signals from exchangeable protons were not always detected. UPLC analysis of final compounds was performed on a Waters Aquity HClass UPLC system and is reported as method name, retention time, UV % purity. The method parameters are as follows;

| Method | Column | Additive | Flow rate | Gradient (time, %MeCN in $H_2O$) |
|---|---|---|---|---|
| A | BEH C18 (130 Å, 1.7 µm, 2.1 mm × 50 mm) | 10 mM $NH_3$ | 0.6 mL/min | 0 min, 5%; 0.8 min, 5%; 3.3 min, 95%; 4.3 min, 95%; 4.5 min, 5%; 5.5 min, 5%. |
| B | HSS C18 (100 Å, 1.8 µm, 2.1 mm × 50 mm) | 0.1% $HCO_2H$ | 0.6 mL/min | 0 min, 5%; 0.8 min, 5%; 3.3 min, 95%; 4.3 min, 95%; 4.5 min, 5%; 5.5 min, 5%. |
| C | BEH C18 (130 Å, 1.7 µm, 2.1 mm × 50 mm) | 10 mM $NH_3$ | 0.6 mL/min | 0 min, 5%; 0.8 min, 5%; 8.3 min, 95%; 9.3 min, 95%; 9.5 min, 5%; 10.5 min, 5%. |
| D | HSS C18 (100 Å, 1.8 µm, 2.1 mm × 50 mm) | 0.1% $HCO_2H$ | 0.6 mL/min | 0 min, 5%; 0.8 min, 5%; 8.3 min, 95%; 9.3 min, 95%; 9.5 min, 5%; 10.5 min, 5%. |

**Abbreviations**: DME: 1,2-dimethoxyethane, DMF: *N*,*N*-dimethylformamide, DMSO: dimethyl sulfoxide, HPLC: high performance liquid chromatography, HRMS: high-resolution mass spectra, LCMS: liquid chromatography-mass spectrometry, rt: room temperature, SEM: 2-(trimethylsilyl)ethoxymethyl, TFA: Trifluoroacetic acid, TMS: tetramethylsilane, UPLC: ultra-performance liquid chromatography.

**General procedure 1:**

A solution of the requisite aryl chloride (1.0 eq), the requisite amine (1.1 eq) and triethylamine (3.0 eq) in $CH_2Cl_2$ (0.5 M) was stirred at rt for the stated period of time. Then the reaction was diluted with $CH_2Cl_2$, washed with brine (2 ×), then dried ($MgSO_4$) and concentrated *in vacuo*.

**General procedure 2:**

A microwave flask was charged with the requisite aryl chloride (1.0 eq) dissolved in DME (2 mL). Then the requisite boronic acid (1.5 eq) and $Na_2CO_3$ (2.0 eq) were added, followed by $H_2O$ (0.5 mL), and the mixture was degassed with $N_2$. Tetrakis(triphenylphosphine)palladium (0.1 eq) was added, the reaction further degassed, then sealed and heated under microwave irradiation for the stated time and temperature. Purification was achieved *via* the stated method.

**General procedure 3:**

A microwave flask was charged with the requisite aryl chloride (1.0 eq) dissolved in DME (2 mL). Then the requisite boronic acid (1.5 eq) and $Na_2CO_3$ (2.0 eq) were added, followed by $H_2O$ (0.5 mL), and the mixture was degassed with $N_2$. Tetrakis(triphenylphosphine)palladium (0.05 eq) was added, the reaction further degassed, then sealed and heated thermally for 4 hours 30 minutes at 90 °C. Purification was achieved *via* the stated method.

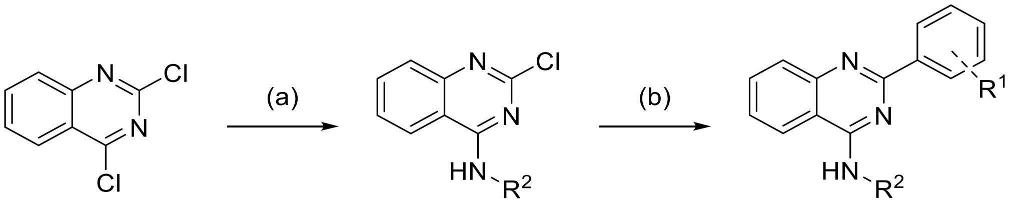


**43** **44-54** **1**, **6-8**, **10-31**

| $R^1$ | $R^2$ | Step (a) product | Step (b) product |
| --- | --- | --- | --- |
| 2-$CF_3$ | 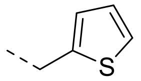 | **44** | **1** |
| H | 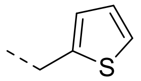 | **44** | **6** |
| 2-$CH_3$ | 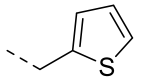 | **44** | **7** |
| 2-$OCH_3$ | 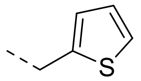 | **44** | **8** |
| 2-$CF_3$ | 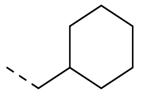 | **45** | **10** |
| 2-$CF_3$ | 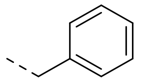 | **46** | **11** |
| 2-$CF_3$ | 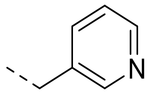 | **47** | **12** |
| 2-$CF_3$ | 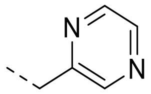 | **48** | **13** |
| 2-$CF_3$ | 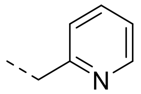 | **49** | **14** |
| 2-$CF_3$ | 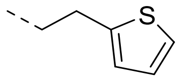 | **50** | **15** |
| 2-$CF_3$-4-F | 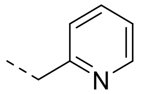 | **49** | **16** |
| 2-$CF_3$-5-F | 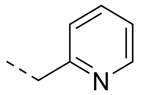 | **49** | **17** |
| 3-$CF_3$ | 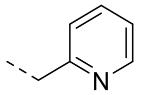 | **49** | **18** |
| 4-$CF_3$ | 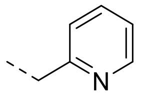 | **49** | **19** |

| $R^1$ | $R^2$ | Step (a) product | Step (b) product |
| --- | --- | --- | --- |
| 3-OMe | 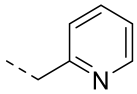 | **49** | **20** |
| 4-OMe | 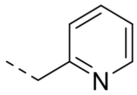 | **49** | **21** |
| 2-$CH(CH_3)_2$ | 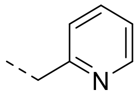 | **49** | **22** |
| 2-cyclopropyl | 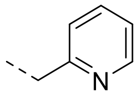 | **49** | **23** |
| 2-$CH_2CH_3$ | 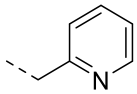 | **49** | **24** |
| 2-Ph | 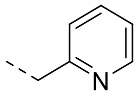 | **49** | **25** |
| 2-CN | 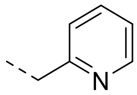 | **49** | **26** |
| 2-$NMe_2$ | 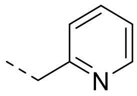 | **49** | **27** |
| 2-$CH(CH_3)_2$ | 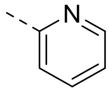 | **51** | **28** |
| 2-$CH(CH_3)_2$ | 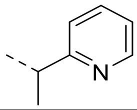 | **52** | **29** |
| 2-$CH(CH_3)_2$ | 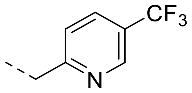 | **53** | **30** |
| 2-$CH(CH_3)_2$ | 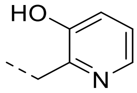 | **54** | **31** |

**Supporting Scheme S1**: *Reagents and conditions*: (a) $R^2$-$NH_2$, $NEt_3$, $CH_2Cl_2$, rt. (b) $R^1$-$B(OH)_2$, $Pd(PPh_3)_4$, $Na_2CO_3$, DME, $H_2O$, 120 °C.

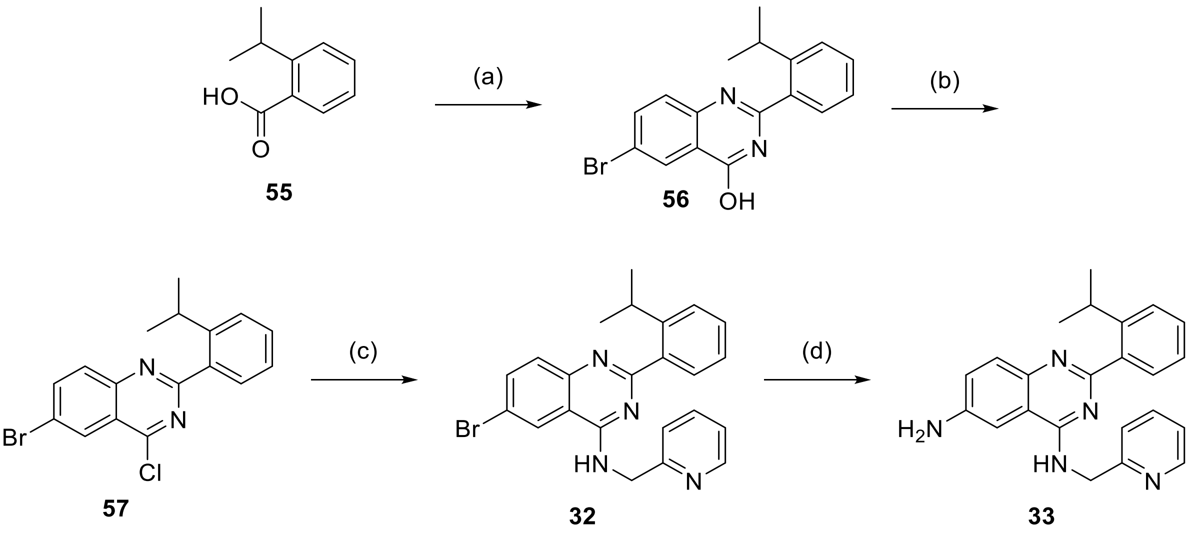


**Supporting Scheme S2**: *Reagents and conditions*: (a) i) $SOCl_2$, DMF, $CH_2Cl_2$, 0 °C to 50 °C. ii) 2-amino-5-bromobenzamide, $NEt_3$, $CH_2Cl_2$, 0 °C to rt. iii) NaOH, $H_2O$, 100 °C. b) $POCl_3$, toluene, 90 °C. c) pyridin-2-ylmethanamine, $NEt_3$, $CH_2Cl_2$, rt. d) $NaN_3$, CuI, sodium ascorbate, *trans*-*N*,*N*'-dimethylcyclohexane-1,2-diamine, EtOH, $H_2O$, 100 °C.

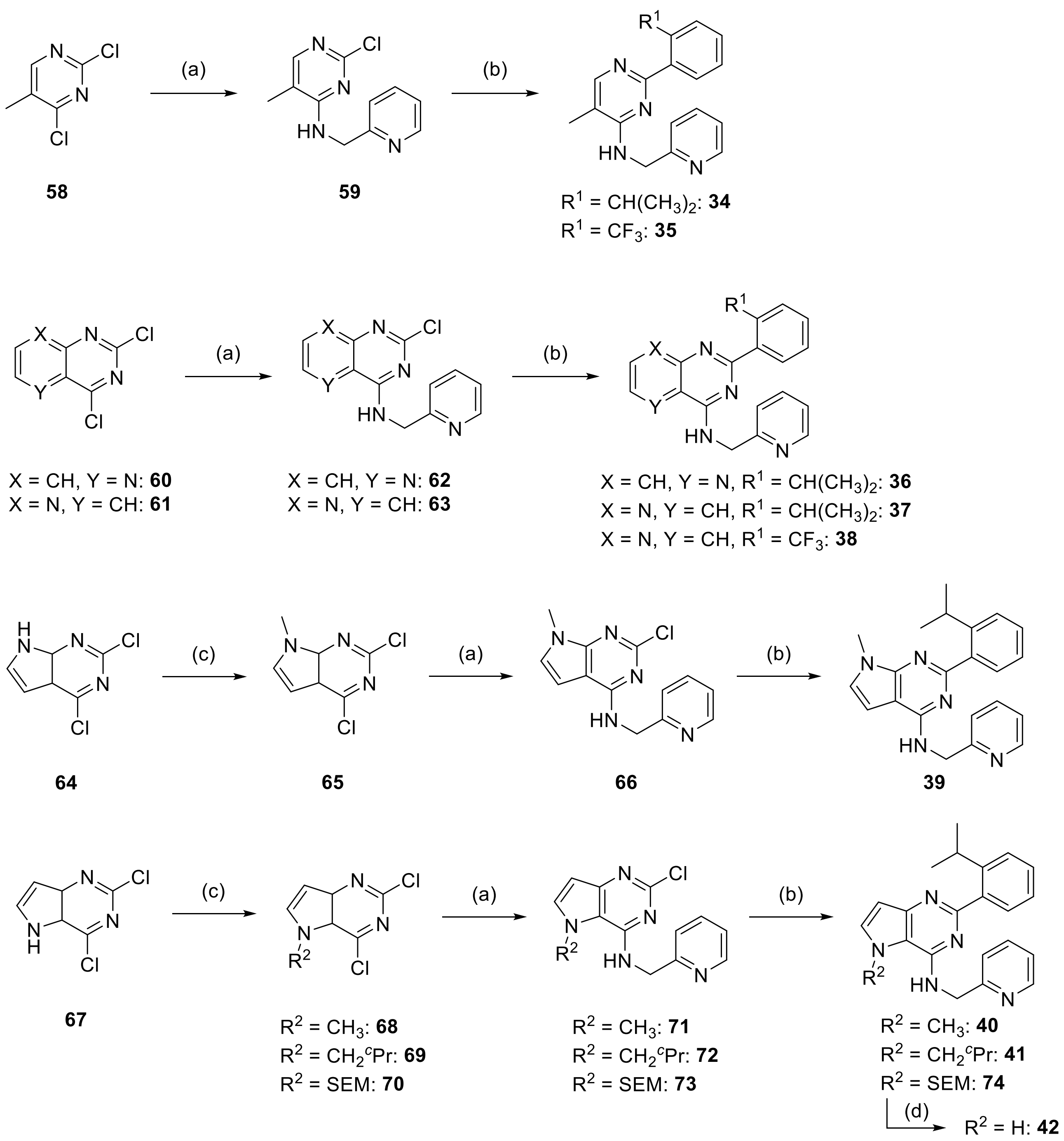


**Supporting Scheme S3**: *Reagents and conditions*: (a) pyridin-2-ylmethanamine, $NEt_3$, $CH_2Cl_2$, rt. (b) (2-propan-2-ylphenyl)boronic acid or (2-trifluoromethyl)phenylboronic acid, $Pd(PPh_3)_4$, $Na_2CO_3$, DME, $H_2O$, 120 °C. (c) $CH_3I$ or ${}^{c}PrCH_2Br$ or SEM-Cl, NaH, DMF, rt. (d) i) TFA, $CH_2Cl_2$, rt. ii) $NH_3$, MeOH, rt.

*2-Chloro-N-(thiophen-2-ylmethyl)quinazolin-4-amine (**44**).*

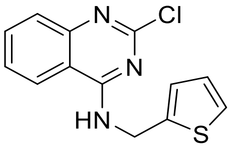


2,4-Dichloroquinazoline **43** (1.00 g, 5.02 mmol) and thiophen-2-ylmethanamine (425 µL, 683 mg, 6.03 mmol) were reacted according to general procedure 1 for 24 hours to give 2-chloro-*N*-(thiophen-2-ylmethyl)quinazolin-4-amine **44** (1.21 g, 4.39 mmol, 87%) as a cream coloured solid which was used without further purification. $^{1}$H NMR (300 MHz, Chloroform-*d*) δ 7.85 – 7.73 (m, 2H), 7.68 (d, *J* = 8.1 Hz, 1H), 7.48 (ddd, *J* = 8.3, 6.6, 1.6 Hz, 1H), 7.31 (dd, *J* = 5.1, 1.2 Hz, 1H), 7.18 – 7.14 (m, 1H), 7.03 (dd, *J* = 5.1, 3.5 Hz, 1H), 6.11 (s, 1H), 5.06 (dd, *J* = 5.3, 0.8 Hz, 2H). Data in accordance with published data.[5]

*N-(Thiophen-2-ylmethyl)-2-(2-(trifluoromethyl)phenyl)quinazolin-4-amine (**1**).*

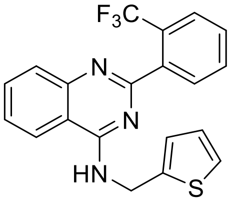


2-Chloro-*N*-(thiophen-2-ylmethyl)quinazolin-4-amine **44** (100 mg, 0.36 mmol) and (2-(trifluoromethyl)phenyl)boronic acid (207 mg, 1.09 mmol) were reacted according to general procedure 2 for 30 min at 120 °C. After concentrating *in vacuo*, purification *via* silica gel chromatography (gradient elution 5 to 40% EtOAc in petroleum ether), followed by preparatory HPLC (gradient elution 25 to 95% MeCN in $H_2O$ with 0.1% $NH_3$) yielded *N*-(thiophen-2-ylmethyl)-2-(2-(trifluoromethyl)phenyl)quinazolin-4-amine **1** (40 mg, 0.10 mmol, 29%) as a white solid. MS (ESI+) *m/z* calcd. for $C_{20}H_{15}N_3F_3S$ $[M+H]^+$ 386.1; found 385.8. HRMS (ESI+) *m/z* calcd. for $C_{20}H_{15}N_3F_3S^+$ 386.0933 $[M+H]^+$; found 386.0934. UPLC (method C) $t_R$ = 5.76 min, >98%. $^{1}$H NMR (300 MHz, DMSO-$d_6$) δ 9.03 (t, *J* = 5.9 Hz, 1H), 8.36 – 8.26 (m, 1H), 7.90 – 7.62 (m, 6H), 7.57 (ddd, *J* = 8.3, 6.8, 1.4 Hz, 1H), 7.36 (dd, *J* = 5.1, 1.3 Hz, 1H), 7.06 (dd, *J* = 3.4, 1.2 Hz, 1H), 6.95 (dd, *J* = 5.1, 3.4 Hz, 1H), 4.97 (d, *J* = 5.6 Hz, 2H). $^{13}$C NMR (75 MHz, DMSO-$d_6$) δ 161.9, 159.4, 150.0, 142.6, 140.3, 133.5 (*C*H), 132.5 (*C*H), 131.7 (*C*H), 129.4 (*C*H), 128.3 (*C*H), 127.5 (q, J = 30.6 Hz), 127.0 (*C*H), 126.9 (q, J = 5.2 Hz, *C*H), 126.6 (*C*H), 126.3 (*C*H), 125.6 (*C*H), 123.1 (*C*H), 122.9, 113.7, 39.1 (*C*$H_2$). $^{19}$F NMR (282 MHz, DMSO-$d_6$) δ -55.89.

*2-Phenyl-N-(thiophen-2-ylmethyl)quinazolin-4-amine (**6**).*

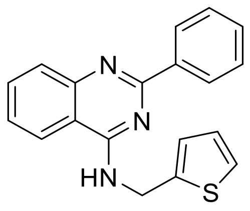

2-Chloro-*N*-(thiophen-2-ylmethyl)quinazolin-4-amine **44** (100 mg, 0.36 mmol) and phenylboronic acid (88 mg, 0.72 mmol) were reacted according to general procedure 2 for 30 min at 120 °C. After concentrating *in vacuo*, purification *via* silica gel chromatography (gradient elution 5 to 40% EtOAc in petroleum ether) yielded 2-phenyl-*N*-(thiophen-2-ylmethyl)quinazolin-4-amine **6** (65 mg, 0.21 mmol, 56%) as a white solid. MS (ESI+) *m*/*z* calcd for $C_{19}H_{16}N_3S$ $[M + H]^+$ 318.1; found 318.1. UPLC (method C) $t_R$ = 5.89 min, >98%. $^1$H NMR (300 MHz, Chloroform-*d*) δ 8.72 – 8.59 (m, 2H), 8.02 – 7.93 (m, 1H), 7.82 – 7.66 (m, 2H), 7.59 – 7.38 (m, 4H), 7.28 – 7.25 (m, 1H), 7.17 (dd, *J* = 3.5, 1.1 Hz, 1H), 7.02 (dd, *J* = 5.1, 3.5 Hz, 1H), 6.09 – 5.94 (m, 1H), 5.21 (d, *J* = 5.5 Hz, 2H).

*N-(Thiophen-2-ylmethyl)-2-(o-tolyl)quinazolin-4-amine (**7**).*

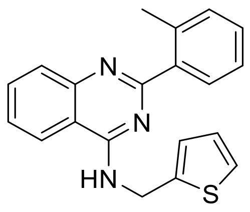

2-Chloro-*N*-(thiophen-2-ylmethyl)quinazolin-4-amine **44** (100 mg, 0.36 mmol) and 2-methylphenylboronic acid (99 mg, 0.72 mmol) were reacted according to general procedure 2 for 30 min at 120 °C. After concentrating *in vacuo*, purification *via* silica gel chromatography (gradient elution 5 to 40% EtOAc in petroleum ether) yielded *N*-(thiophen-2-ylmethyl)-2-(o-tolyl)quinazolin-4-amine **7** (12 mg, 0.036 mmol, 10%) as a white oily solid. MS (ESI+) *m*/*z* calcd for $C_{20}H_{18}N_3S$ $[M + H]^+$ 332.1; found 332.1. UPLC (method C) $t_R$ = 5.73 min, >98%. $^1$H NMR (300 MHz, Chloroform-*d*) δ 8.00 – 7.90 (m, 2H), 7.82 – 7.69 (m, 2H), 7.48 (ddd, *J* = 8.2, 6.9, 1.3 Hz, 1H), 7.39 – 7.25 (m, 4H), 7.12 (dt, *J* = 3.0, 1.1 Hz, 1H), 7.02 (dd, *J* = 5.1, 3.5 Hz, 1H), 6.02 – 5.89 (m, 1H), 5.14 (dd, *J* = 5.3, 0.9 Hz, 2H), 2.64 (s, 3H).

*2-(2-Methoxyphenyl)-N-(thiophen-2-ylmethyl)quinazolin-4-amine (**8**).*

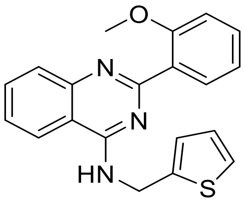

2-Chloro-*N*-(thiophen-2-ylmethyl)quinazolin-4-amine **44** (100 mg, 0.36 mmol) and 2-methoxyphenylboronic acid (110 mg, 0.72 mmol) were reacted according to general procedure 2 for 30 min at 120 °C. After concentrating *in vacuo*, purification *via* silica gel chromatography (gradient elution 5 to 80% EtOAc in petroleum ether) yielded 2-(2-methoxyphenyl)-*N*-(thiophen-2-ylmethyl)quinazolin-4-amine **8** (103 mg, 0.296 mmol, 82%) as a yellow solid. MS (ESI+) *m*/*z* calcd for $C_{20}H_{18}N_3OS$ $[M + H]^+$ 348.1; found 348.1. UPLC (method C) $t_R$ = 5.02 min, >98%. $^1H$ NMR (300 MHz, Chloroform-*d*) δ 8.01 – 7.94 (m, 1H), 7.85 (dd, *J* = 7.5, 1.8 Hz, 1H), 7.80 – 7.69 (m, 2H), 7.51 – 7.36 (m, 2H), 7.27 (dd, *J* = 5.1, 1.2 Hz, 1H), 7.18 – 6.96 (m, 4H), 6.01 (s, 1H), 5.11 (d, *J* = 4.0 Hz, 2H), 3.90 (s, 3H).

*2-Chloro-N-(cyclohexylmethyl)quinazolin-4-amine (**45**).*

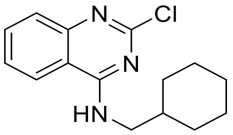

2,4-Dichloroquinazoline **43** (1.00 g, 5.03 mmol) and cyclohexanemethanamine (783 µL, 681 mg, 6.03 mmol) were reacted according to general procedure 1 for 18 hours. Purification *via* silica gel chromatography (gradient elution 0 to 50% EtOAc in petroleum ether) yielded 2-chloro-*N*-(cyclohexylmethyl)quinazolin-4-amine **45** (1.33 g, 4.82 mmol, 96%). MS (ESI+) *m*/*z* calcd for $C_{15}H_{19}ClN_3$ $[M + H]^+$ 276.1; found 276.0. UPLC (method A) $t_R$ = 3.27 min, >95%.

*N-(Cyclohexylmethyl)-2-(2-(trifluoromethyl)phenyl)quinazolin-4-amine (**10**).*

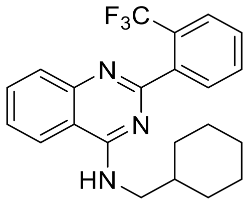

2-Chloro-*N*-(cyclohexylmethyl)quinazolin-4-amine **45** (100 mg, 0.36 mmol) and (2-(trifluoromethyl)phenyl)boronic acid (104 mg, 0.55 mmol) were reacted according to general procedure 2 for 40 min at 125 °C. After concentrating *in vacuo*, purification *via* silica gel chromatography (gradient elution 0 to 50% EtOAc in petroleum ether) yielded *N*-(cyclohexylmethyl)-2-(2-(trifluoromethyl)phenyl)quinazolin-4-amine **10** (87 mg, 0.23 mmol, 63%) as a white solid. MS (ESI+) *m*/*z* calcd for $C_{22}H_{23}F_3N_3$ $[M + H]^+$ 386.2; found 386.0. UPLC (method C) $t_R$ = 6.64 min, 98%. $^1$H NMR (300 MHz, Chloroform-*d*) δ 7.94 (dt, *J* = 8.2, 1.0 Hz, 1H), 7.88 – 7.71 (m, 4H), 7.64 (td, *J* = 7.5, 1.4 Hz, 1H), 7.57 – 7.49 (m, 2H), 5.84 (s, 1H), 3.58 (dd, *J* = 6.8, 5.7 Hz, 2H), 1.90 – 1.55 (m, 7H), 1.38 – 1.13 (m, 4H).

*N-Benzyl-2-chloroquinazolin-4-amine (**46**).*

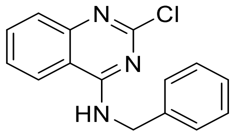

2,4-Dichloroquinazoline **43** (1.00 g, 5.03 mmol) and benzylamine (602 µL, 591 mg, 5.52 mmol) were reacted according to general procedure 1 for 3 hours. Purification *via* silica gel chromatography (gradient elution 0 to 50% EtOAc in petroleum ether) yielded *N*-benzyl-2-chloroquinazolin-4-amine **46** (1.32 g, 4.89 mmol, 97%). MS (ESI+) *m*/*z* calcd for $C_{15}H_{13}ClN_3$ $[M + H]^+$ 270.1; found 269.9. UPLC (method A) $t_R$ = 2.94 min, 90%.

*N-Benzyl-2-(2-(trifluoromethyl)phenyl)quinazolin-4-amine (**11**).*

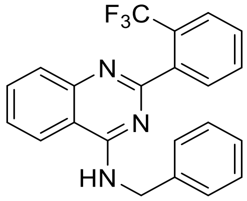

*N*-Benzyl-2-chloroquinazolin-4-amine **46** (100 mg, 0.37 mmol) and (2-(trifluoromethyl)phenyl)boronic acid (106 mg, 0.56 mmol) were reacted according to general procedure 2 for 35 min at 125 °C. After concentrating *in vacuo*, purification *via* silica gel chromatography (gradient elution 0 to 50% EtOAc in petroleum ether) yielded

*N*-benzyl-2-(2-(trifluoromethyl)phenyl)quinazolin-4-amine **11** (127 mg, 0.336 mmol, 91%) as a white solid. MS (ESI+) *m*/*z* calcd for $C_{22}H_{17}F_3N_3$ [M + H]$^+$ 380.1; found 379.9. UPLC (method C) $t_R$ = 5.89 min, >98%. $^1$H NMR (300 MHz, Chloroform-*d*) δ $^1$H NMR (300 MHz, Chloroform-*d*) δ 8.01 – 7.92 (m, 1H), 7.92 – 7.61 (m, 5H), 7.61 – 7.30 (m, 7H), 5.95 (s, 1H), 4.94 (d, *J* = 5.3 Hz, 2H).

*2-Chloro-N-(pyridin-3-ylmethyl)quinazolin-4-amine (**47**).*

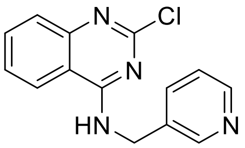


2,4-Dichloroquinazoline **43** (1.50 g, 7.54 mmol) and 3-aminomethylpyridine (844 µL, 897 mg, 8.29 mmol) were reacted according to general procedure 1 for 18 hours. Purification *via* silica gel chromatography (gradient elution 0 to 100% EtOAc in petroleum ether) yielded 2-chloro-*N*-(pyridin-3-ylmethyl)quinazolin-4-amine **47** (1.83 g, 6.74 mmol, 89%). MS (ESI+) *m*/*z* calcd for $C_{14}H_{12}ClN_4$ [M + H]$^+$ 271.1; found 271.1. UPLC (method A) $t_R$ = 2.45 min, 80%.

*N-(Pyridin-3-ylmethyl)-2-(2-(trifluoromethyl)phenyl)quinazolin-4-amine (**12**).*

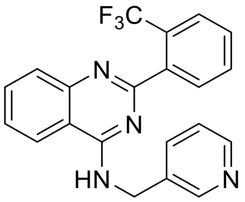


2-Chloro-*N*-(pyridin-3-ylmethyl)quinazolin-4-amine **47** (100 mg, 0.37 mmol) and (2-(trifluoromethyl)phenyl)boronic acid (106 mg, 0.56 mmol) were reacted according to general procedure 2 for 40 min at 125 °C. After concentrating *in vacuo*, purification *via* silica gel chromatography (gradient elution 0 to 100% EtOAc in petroleum ether) yielded *N*-(pyridin-3-ylmethyl)-2-(2-(trifluoromethyl)phenyl)quinazolin-4-amine **12** (111 mg, 0.292 mmol, 79%) as a white solid. MS (ESI+) *m*/*z* calcd for $C_{21}H_{16}F_3N_4$ [M + H]$^+$ 381.1; found 381.1. UPLC (method C) $t_R$ = 4.72 min, >98%. $^1$H NMR (300 MHz, DMSO-$d_6$) δ 8.99 (t, *J* = 5.9 Hz, 1H), 8.62 – 8.51 (m, 1H), 8.44 (dd, *J* = 4.8, 1.7 Hz, 1H), 8.38 – 8.24 (m, 1H), 7.86 – 7.71 (m, 5H), 7.70 – 7.50 (m, 3H), 7.32 (ddd, *J* = 7.9, 4.8, 0.9 Hz, 1H), 4.83 (d, *J* = 5.7 Hz, 2H).

*2-Chloro-N-(pyrazin-2-ylmethyl)quinazolin-4-amine (**48**).*

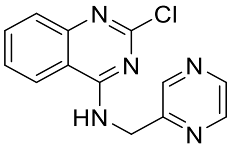

2,4-Dichloroquinazoline **43** (1.50 g, 7.54 mmol) and 2-aminomethylpyrazine (794 µL, 904 mg, 8.29 mmol) were reacted according to general procedure 1 for 2 hours. Purification *via* silica gel chromatography (gradient elution 0 to 100% EtOAc in petroleum ether) yielded 2-chloro-*N*-(pyrazin-2-ylmethyl)quinazolin-4-amine **48** (1.26 g, 4.64 mmol, 62%). MS (ESI+) *m*/*z* calcd for $C_{13}H_{11}ClN_5$ $[M + H]^+$ 272.1; found 272.0. UPLC (method A) $t_R$ = 2.33 min, 72%.

*N-(Pyrazin-2-ylmethyl)-2-(2-(trifluoromethyl)phenyl)quinazolin-4-amine (**13**).*

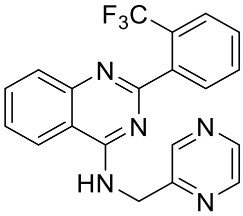

2-Chloro-*N*-(pyrazin-2-ylmethyl)quinazolin-4-amine **48** (100 mg, 0.37 mmol) and (2-(trifluoromethyl)phenyl)boronic acid (104 mg, 0.55 mmol) were reacted according to general procedure 2 for 40 min at 125 °C. After concentrating *in vacuo*, purification *via* silica gel chromatography (gradient elution 0 to 100% EtOAc in petroleum ether) yielded *N*-(pyrazin-2-ylmethyl)-2-(2-(trifluoromethyl)phenyl)quinazolin-4-amine **13** (71 mg, 0.19 mmol, 51%) as a white solid. MS (ESI+) *m*/*z* calcd for $C_{20}H_{15}F_3N_5$ $[M + H]^+$ 382.1; found 382.1. UPLC (method C) $t_R$ = 4.45 min, >98%. $^1$H NMR (300 MHz, Methanol-d4) δ 8.61 (d, J = 1.5 Hz, 1H), 8.56 (dd, J = 2.6, 1.5 Hz, 1H), 8.51 – 8.43 (m, 1H), 8.26 (ddd, J = 8.3, 1.4, 0.7 Hz, 1H), 7.87 (ddd, J = 8.1, 6.6, 1.4 Hz, 1H), 7.81 (ddd, J = 8.4, 1.6, 0.7 Hz, 1H), 7.79 – 7.74 (m, 1H), 7.73 – 7.66 (m, 1H), 7.63 (ddt, J = 7.5, 6.8, 1.5 Hz, 3H), 5.02 (s, 2H).

*2-Chloro-N-(pyridin-2-ylmethyl)quinazolin-4-amine (**49**).*

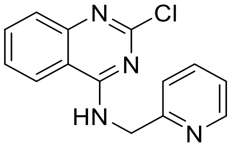

2,4-Dichloroquinazoline **43** (1.00 g, 5.03 mmol) and 2-aminomethylpyridine (569 µL, 597 mg, 5.53 mmol) were reacted according to general procedure 1 for 18 hours. Purification *via* silica gel chromatography (gradient elution 0 to 100% EtOAc in petroleum ether) yielded 2-chloro-*N*-(pyridin-2-ylmethyl)quinazolin-4-amine **49** (852 mg, 3.15 mmol, 63%). MS (ESI+) *m/z* calcd for $C_{14}H_{12}ClN_4$ $[M + H]^+$ 271.1; found 270.9. UPLC (method A) $t_R$ = 2.54 min, 86%.

*N-(Pyridin-2-ylmethyl)-2-(2-(trifluoromethyl)phenyl)quinazolin-4-amine (**14**).*

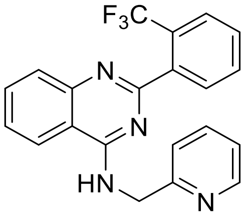


2-Chloro-*N*-(pyridin-2-ylmethyl)quinazolin-4-amine **49** (100 mg, 0.37 mmol) and (2-(trifluoromethyl)phenyl)boronic acid (106 mg, 0.56 mmol) were reacted according to general procedure 2 for 35 min at 125 °C. After concentrating *in vacuo*, purification *via* silica gel chromatography (gradient elution 0 to 100% EtOAc in petroleum ether) yielded *N*-(pyridin-2-ylmethyl)-2-(2-(trifluoromethyl)phenyl)quinazolin-4-amine **14** (102 mg, 0.268 mmol, 72%) as a colourless solid. MS (ESI+) *m/z* calcd. for $C_{21}H_{16}N_4F_3$ $[M+H]^+$ 381.1; found 380.9. HRMS (ES+) *m/z* calcd. for $C_{21}H_{16}N_4F_3^+$ $[M+H]^+$ 381.1322; found 381.1324. UPLC (method A) $t_R$ = 4.94 min, >98%. $^1$H NMR (300 MHz, Chloroform-*d*) δ 8.65 (ddd, *J* = 5.0, 1.8, 1.0 Hz, 1H), 8.04 – 7.92 (m, 2H), 7.91 – 7.61 (m, 6H), 7.60 – 7.52 (m, 2H), 7.36 (dt, *J* = 7.9, 1.0 Hz, 1H), 7.29 – 7.24 (m, 1H), 4.99 (d, *J* = 4.3 Hz, 2H). $^{13}$C NMR (75 MHz, DMSO-d$_6$) δ 162.0, 159.9, 159.1, 150.1, 149.3, 140.3 (q, *J* = 2.2 Hz), 137.0, 133.5, 132.4, 131.7, 129.3, 128.3, 127.5 (q, *J* = 30.6 Hz), 126.8 (q, *J* = 5.2 Hz), 126.5, 124.6 (q, *J* = 273.6 Hz), 123.2, 122.5, 121.4, 113.8, 46.0. $^{19}$F NMR (282 MHz, DMSO-d$_6$) δ -56.10.

*2-Chloro-N-(2-(thiophen-2-yl)ethyl)quinazolin-4-amine (**50**).*

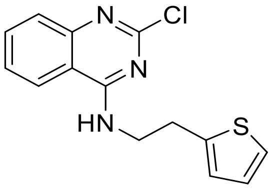


2,4-Dichloroquinazoline **43** (1.00 g, 5.03 mmol) and thiophen-2-ethylamine (705 µL, 766 mg, 6.03 mmol) were reacted according to general procedure 1 for 3 hours to give 2-chloro-*N*-(2-(thiophen-2-yl)ethyl)quinazolin-4-

amine **50** (1.45 g, 5.00 mmol, 99%) which was used without further purification. MS (ESI+) *m*/*z* calcd for $C_{14}H_{13}ClN_3S$ [M + H]$^+$ 290.1; found 289.9. UPLC (method A) $t_R$ = 3.07 min, 93%.

*N-(2-(Thiophen-2-yl)ethyl)-2-(2-(trifluoromethyl)phenyl)quinazolin-4-amine (**15**).*

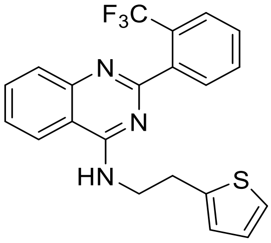

2-Chloro-*N*-(2-(thiophen-2-yl)ethyl)quinazolin-4-amine **50** (100 mg, 0.35 mmol) and (2-(trifluoromethyl)phenyl)boronic acid (99 mg, 0.52 mmol) were reacted according to general procedure 2 for 45 min at 125 °C. After concentrating *in vacuo*, purification *via* silica gel chromatography (gradient elution 0 to 50% EtOAc in petroleum ether) yielded *N*-(2-(thiophen-2-yl)ethyl)-2-(2-(trifluoromethyl)phenyl)quinazolin-4-amine **15** (121 mg, 0.303 mmol, 87%) as a white solid. MS (ESI+) *m*/*z* calcd for $C_{21}H_{17}F_3N_3S$ [M + H]$^+$ 400.1; found 399.9. UPLC (method C) $t_R$ = 6.02 min, >98%. $^1$H NMR (300 MHz, Chloroform-*d*) δ 7.99 – 7.72 (m, 4H), 7.72 – 7.60 (m, 2H), 7.63 – 7.42 (m, 2H), 7.22 (dd, *J* = 5.1, 1.2 Hz, 1H), 7.00 (dd, *J* = 5.1, 3.4 Hz, 1H), 6.91 (dt, *J* = 3.4, 1.0Hz, 1H), 5.93 (s, 1H), 4.01 (q, *J* = 6.3 Hz, 2H), 3.27 (td, *J* = 6.5, 0.8 Hz, 2H).

*2-(4-Fluoro-2-(trifluoromethyl)phenyl)-N-(pyridin-2-ylmethyl)quinazolin-4-amine (**16**).*

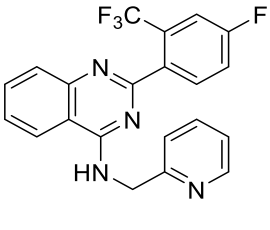

2-Chloro-*N*-(pyridin-2-ylmethyl)quinazolin-4-amine **49** (50 mg, 0.180 mmol) and 4-fluoro-2-(trifluoromethyl)phenyl]boronic acid (57.6 mg, 0.280 mmol) were reacted according to general procedure 2 for 45 mins at 120 °C. Upon cooling to room temperature the reaction mixture was loaded onto an SCX-II column, washed with MeOH, then eluted with 0.5 M $NH_3$ in MeOH and concentrated *in vacuo*. Purification *via* preparatory HPLC (gradient elution 30 to 70% MeCN in $H_2O$ with 0.1% $NH_3$) yielded 2-(4-fluoro-2-(trifluoromethyl)phenyl)-*N*-(pyridin-2-ylmethyl)quinazolin-4-amine **16** (46.5 mg, 0.117 mmol, 63%) as a white solid. MS (ESI+) *m/z* calcd for $C_{21}H_{15}F_4N_4$ [M + H]$^+$ 399.1; found 399.2. UPLC (method C) $t_R$ = 5.25 min, >98%. $^1$H NMR (300 MHz,

Chloroform-d) δ 8.66 (dt, *J* = 4.7, 1.4 Hz, 1H), 8.00 (dd, *J* = 8.3, 1.3 Hz, 1H), 7.92 (td, *J* = 5.5, 2.7 Hz, 2H), 7.80 (ddd, *J* = 8.3, 7.0, 1.4 Hz, 1H), 7.73 (td, *J* = 7.7, 1.8 Hz, 1H), 7.66 (s, 1H), 7.63 – 7.47 (m, 2H), 7.41 – 7.32 (m, 2H), 7.32 – 7.24 (m, 1H), 4.97 (d, *J* = 4.3 Hz, 2H).

*2-(5-Fluoro-2-(trifluoromethyl)phenyl)-N-(pyridin-2-ylmethyl)quinazolin-4-amine (**17**).*

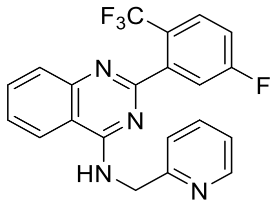


2-Chloro-*N*-(pyridin-2-ylmethyl)quinazolin-4-amine **49** (100 mg, 0.370 mmol) and 5-fluoro-2-(trifluoromethyl)phenyl]boronic acid (115 mg, 0.550 mmol) were reacted according to general procedure 2 for 45 mins at 120 °C. Upon cooling to room temperature the reaction mixture was loaded onto an SCX-II column, washed with MeOH, then eluted with 0.5 M $NH_3$ in MeOH and concentrated *in vacuo*. Purification *via* preparatory HPLC (gradient elution 30 to 70% MeCN in $H_2O$ with 0.1% $NH_3$) yielded 2-(5-fluoro-2-(trifluoromethyl)phenyl)-*N*-(pyridin-2-ylmethyl)quinazolin-4-amine **17** (82.3 mg, 0.413 mmol, 56%) as a white solid. MS (ESI+) *m/z* calcd for $C_{21}H_{15}F_4N_4$ $[M + H]^+$ 399.1; found 399.2. UPLC (method C) $t_R$ = 5.28 min, >98%. $^1$H NMR (300 MHz, Chloroform-d) δ 8.65 (d, *J* = 5.0 Hz, 1H), 7.96 (dd, *J* = 21.7, 8.3 Hz, 2H), 7.88 – 7.67 (m, 4H), 7.67 – 7.48 (m, 2H), 7.36 (d, *J* = 7.9 Hz, 1H), 7.26 (dt, *J* = 17.8, 5.4 Hz, 2H), 4.97 (d, *J* = 4.4 Hz, 2H).

*N-(Pyridin-2-ylmethyl)-2-(3-(trifluoromethyl)phenyl)quinazolin-4-amine (**18**).*

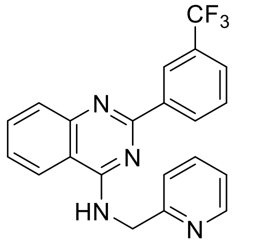


2-Chloro-*N*-(pyridin-2-ylmethyl)quinazolin-4-amine **49** (50 mg, 0.185 mmol) and [3-(trifluoromethyl)phenyl]boronic acid (53.0 mg, 0.22 mmol) were reacted according to general procedure 3,

resulting in partial conversion to desired product. A further 1 equivalent of [3-(trifluoromethyl)phenyl]boronic acid (35.5 mg, 0.15 mmol) and 0.05 equivalents of tetrakis(triphenylphosphine)palladium (10 mg, 0.01 mmol) were added to the reaction mixture with degassing and further heating for 18 hours at 90 °C, followed by 3 hours at 110 °C. Upon cooling to room temperature the reaction mixture purified directly *via* silica gel chromatography (gradient elution 10 to 80% EtOAc in petroleum ether) followed by preparatory HPLC (gradient elution 30 to 80% MeCN in $H_2O$ with 0.1% $NH_3$) yielding *N*-(pyridin-2-ylmethyl)-2-(3-(trifluoromethyl)phenyl)quinazolin-4-amine **18** (13.0 mg, 0.034 mmol, 19%) as a white solid. MS (ESI+) *m/z* calcd for $C_{21}H_{16}F_3N_4$ $[M + H]^+$ 381.1; found 381.2. UPLC (method C) $t_R$ = 5.97 min, >98%. $^1H$ NMR (300 MHz, Chloroform-*d*) δ 8.92 – 8.76 (m, 2H), 8.67 (ddd, *J* = 4.9, 1.8, 0.9 Hz, 1H), 8.01 – 7.91 (m, 2H), 7.79 (ddd, *J* = 8.9, 7.2, 1.5 Hz, 2H), 7.75 – 7.71 (m, 1H), 7.70 – 7.58 (m, 1H), 7.58 – 7.45 (m, 3H), 7.34 – 7.23 (m, 1H, obsc. by solvent peak), 5.09 (d, *J* = 4.5 Hz, 2H).

*N-(Pyridin-2-ylmethyl)-2-(4-(trifluoromethyl)phenyl)quinazolin-4-amine (**19**).*

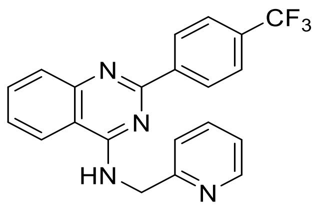

2-Chloro-*N*-(pyridin-2-ylmethyl)quinazolin-4-amine **49** (50.0 mg, 0.185 mmol) and [4-(trifluoromethyl)phenyl]boronic acid (53.0 mg, 0.22 mmol) were reacted according to general procedure 3, resulting in partial conversion to desired product. A further 1 equivalent of [4-(trifluoromethyl)phenyl]boronic acid (35.5 mg, 0.15 mmol) and 0.05 equivalents of tetrakis(triphenylphosphine)palladium (10 mg, 0.01 mmol) were added to the reaction mixture with degassing and further heating for 18 hours at 90 °C, followed by 3 hours at 110 °C. Upon cooling to room temperature the reaction mixture was purified directly *via* silica gel chromatography (gradient elution 10 to 80% EtOAc in petroleum ether) followed by preparatory HPLC (gradient elution 30 to 80% MeCN in $H_2O$ with 0.1% $NH_3$) yielding *N*-(pyridin-2-ylmethyl)-2-(4-(trifluoromethyl)phenyl)quinazolin-4-amine **19** (18.0 mg, 0.047 mmol, 26%) as a white solid. MS (ESI+) *m/z* calcd for $C_{21}H_{16}F_3N_4$ $[M + H]^+$ 381.1; found 381.2. UPLC (method C) $t_R$ = 6.04 min, >98%. $^1H$ NMR (300 MHz, Chloroform-*d*) δ 8.72 (dp, *J* = 7.6, 0.9 Hz, 2H), 8.68 (ddd, *J* = 4.9, 1.8, 1.0 Hz, 1H), 7.97 (dq, *J* = 7.9, 0.6 Hz, 2H), 7.86 – 7.69 (m, 4H), 7.56-7.50 (m, 1H), 7.49 (brs, 1H), 7.48-7.44 (m, 1H), 7.35 – 7.24 (m, 1H, obsc. by solvent peak), 5.09 (d, *J* = 4.5 Hz, 2H).

*2-(3-Methoxyphenyl)-N-(pyridin-2-ylmethyl)quinazolin-4-amine (**20**).*

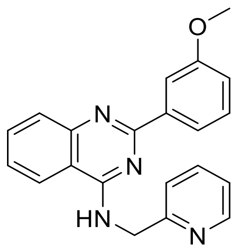

2-Chloro-*N*-(pyridin-2-ylmethyl)quinazolin-4-amine **49** (50 mg, 0.185 mmol) and (3-methoxyphenyl)boronic acid (41.0 mg, 0.22 mmol) were reacted according to general procedure 3, resulting in partial conversion to desired product. A further 1 equivalent of (3-methoxyphenyl)boronic acid (27.3 mg, 0.15 mmol) and 0.05 equivalents of tetrakis(triphenylphosphine)palladium (10 mg, 0.01 mmol) were added to the reaction mixture with degassing and further heating for 18 hours at 90 °C, followed by 3 hours at 110 °C. Upon cooling to room temperature the reaction mixture purified directly *via* silica gel chromatography (gradient elution 10 to 80% EtOAc in petroleum ether) followed by preparatory HPLC (gradient elution 30 to 80% MeCN in $H_2O$ with 0.1% $NH_3$) yielded 2-(3-methoxyphenyl)-*N*-(pyridin-2-ylmethyl)quinazolin-4-amine **20** (22.0 mg, 0.064 mmol, 36%) as a white solid. MS (ESI+) *m/z* calcd for $C_{21}H_{19}N_4O$ $[M + H]^+$ 343.2; found 343.2. UPLC (method C) $t_R$ = 4.98 min, >98%. $^1H$ NMR (300 MHz, Chloroform-*d*) δ 8.67 (ddd, *J* = 5.0, 1.8, 0.9 Hz, 1H), 8.23 (dt, *J* = 7.7, 1.2 Hz, 1H), 8.19 (dd, *J* = 2.7, 1.4 Hz, 1H), 8.01 – 7.89 (m, 2H), 7.83 – 7.68 (m, 2H), 7.53 – 7.41 (m, 3H), 7.41-7.34 (m, 1H), 7.31 – 7.24 (m, 1H, obsc. by solvent peak), 7.05 (ddd, *J* = 8.2, 2.7, 1.0 Hz, 1H), 5.10 (d, *J* = 4.5 Hz, 2H), 3.97 (s, 3H).

*2-(4-Methoxyphenyl)-N-(pyridin-2-ylmethyl)quinazolin-4-amine (**21**).*

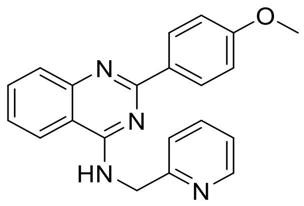

2-Chloro-*N*-(pyridin-2-ylmethyl)quinazolin-4-amine **49** (50 mg, 0.185 mmol) and (4-methoxyphenyl)boronic acid (41.0 mg, 0.22 mmol) were reacted according to general procedure 3. Upon cooling to room temperature the reaction mixture was purified directly *via* silica gel chromatography (gradient elution 10 to 80% EtOAc in petroleum ether) yielding 2-(4-methoxyphenyl)-*N*-(pyridin-2-ylmethyl)quinazolin-4-amine **21** (20.0 mg, 0.058

mmol, 32%) as a white solid. MS (ESI+) *m/z* calcd for $C_{21}H_{19}N_4O$ [M + H]$^+$ 343.2; found 343.1. UPLC (method C) $t_R$ = 4.98 min, 98%. $^1$H NMR (300 MHz, Chloroform-*d*) δ 8.66 (ddd, *J* = 4.9, 1.8, 0.9 Hz, 1H), 8.22 (ddd, *J* = 7.7, 1.5, 1.0 Hz, 1H), 8.18 (dd, *J* = 2.6, 1.5 Hz, 1H), 8.01 – 7.89 (m, 2H), 7.80 – 7.70 (m, 2H), 7.53 – 7.41 (m, 3H), 7.40 (s, 1H), 7.33 – 7.22 (m, 1H, obsc. by solvent peak), 7.05 (ddd, *J* = 8.2, 2.7, 1.0 Hz, 1H), 5.10 (d, *J* = 4.5 Hz, 2H), 3.96 (s, 3H).

*2-(2-Isopropylphenyl)-N-(pyridin-2-ylmethyl)quinazolin-4-amine (**22**).*

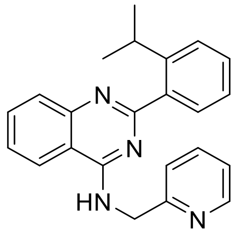


2-Chloro-*N*-(pyridin-2-ylmethyl)quinazolin-4-amine **49** (50 mg, 0.185 mmol) and (2-propan-2-ylphenyl)boronic acid (45.0 mg, 0.22 mmol) were reacted according to general procedure 3, resulting in partial conversion to desired product. A further 1 equivalent of (2-propan-2-ylphenyl)boronic acid (30 mg, 0.15 mmol) and 0.05 equivalents of tetrakis(triphenylphosphine)palladium (10 mg, 0.01 mmol) were added to the reaction mixture with degassing and further heating for 18 hours at 90 °C. Upon cooling to room temperature the reaction mixture was purified directly *via* silica gel chromatography (gradient elution 10 to 80% EtOAc in petroleum ether) yielding 2-(2-isopropylphenyl)-*N*-(pyridin-2-ylmethyl)quinazolin-4-amine **22** (31.0 mg, 0.087 mmol, 49%) as a white solid. MS (ESI+) *m*/*z* calcd. for $C_{23}H_{23}N_4$ [M + H]$^+$ 355.2; found 355.1. HRMS (ES+) *m/z* calcd. for $C_{23}H_{23}N_4^+$ [M + H]$^+$ 355.1917; found 355.1923. UPLC (method C) $t_R$ = 5.31 min, 97%. $^1$H NMR (300 MHz, DMSO-$d_6$) δ 8.99 (t, *J* = 6.0 Hz, 1H), 8.51 (ddd, *J* = 4.9, 1.9, 1.0 Hz, 1H), 8.43 – 8.34 (m, 1H), 7.86 – 7.68 (m, 3H), 7.61 – 7.54 (m, 1H), 7.47 (dt, *J* = 7.6, 1.1 Hz, 1H), 7.39 – 7.14 (m, 5H), 4.90 (d, *J* = 5.8 Hz, 2H), 3.46 (p, *J* = 6.9 Hz, 1H), 0.96 (d, *J* = 6.9 Hz, 6H). $^{13}$C NMR (75 MHz, DMSO-*d*$_6$) δ 164.0, 159.8, 159.3, 150.3, 149.4, 147.2, 139.7, 137.1, 133.3, 130.3, 129.0, 128.3, 126.1, 125.8, 125.5, 123.1, 122.4, 121.0, 113.6, 46.0, 29.0, 24.2.

*2-(2-Cyclopropylphenyl)-N-(pyridin-2-ylmethyl)quinazolin-4-amine (**23**).*

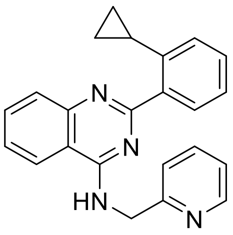

2-Chloro-*N*-(pyridin-2-ylmethyl)quinazolin-4-amine **49** (50 mg, 0.180 mmol) and (2-cyclopropylphenyl)boronic acid (44.7 mg, 0.276 mmol) were reacted according to general procedure 2 for 45 mins at 120 °C. Upon cooling to room temperature the reaction mixture was loaded onto an SCX-II column, washed with MeOH, then eluted with 0.5 M $NH_3$ in MeOH and concentrated *in vacuo*. Purification *via* preparatory HPLC (gradient elution 40 to 80% MeCN in $H_2O$ with 0.1% $NH_3$) yielded 2-(2-cyclopropylphenyl)-*N*-(pyridin-2-ylmethyl)quinazolin-4-amine **23** (29.9 mg, 0.085 mmol, 46%) as a white solid. MS (ESI+) *m/z* calcd for $C_{23}H_{21}N_4$ $[M + H]^+$ 353.2; found 353.2. UPLC (method C) $t_R$ = 5.00 min, 99%. $^1$H NMR (300 MHz, Chloroform-d) δ 8.65 (dt, *J* = 4.9, 1.3 Hz, 1H), 7.97 (td, *J* = 8.5, 1.3 Hz, 2H), 7.84 – 7.65 (m, 3H), 7.58 – 7.45 (m, 2H), 7.41 – 7.21 (m, 4H), 7.04 (dd, *J* = 7.6, 1.5 Hz, 1H), 5.02 (d, *J* = 4.4 Hz, 2H), 2.62 (tt, *J* = 8.5, 5.4 Hz, 1H), 0.85 – 0.73 (m, 2H), 0.69 (ddd, *J* = 7.0, 5.4, 3.4 Hz, 2H).

*2-(2-Ethylphenyl)-N-(pyridin-2-ylmethyl)quinazolin-4-amine (**24**).*

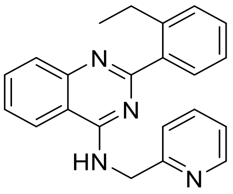

2-Chloro-*N*-(pyridin-2-ylmethyl)quinazolin-4-amine **49** (50 mg, 0.185 mmol) and (2-ethylphenyl)boronic acid (55.4 mg, 0.369 mmol) were reacted according to general procedure 2 for 2 hours at 120 °C. Upon cooling to room temperature the reaction mixture was loaded onto an SCX-II column, washed with MeOH, then eluted with 0.5 M $NH_3$ in MeOH and concentrated *in vacuo*. Purification *via* preparatory HPLC (gradient elution 5 to 95% MeCN in $H_2O$ with 0.1% $NH_3$) yielded 2-(2-ethylphenyl)-*N*-(pyridin-2-ylmethyl)quinazolin-4-amine **24** (50.3 mg, 0.148 mmol, 80%) as a white solid. MS (ESI+) *m/z* calcd for $C_{22}H_{21}N_4$ $[M + H]^+$ 341.2; found 341.1. UPLC (method C) $t_R$ = 5.15 min, >98%. $^1$H NMR (300 MHz, Chloroform-d) δ 8.66 (ddd, *J* = 4.9, 1.8, 1.0 Hz, 1H), 8.01

– 7.97 (m, 1H), 7.95 (ddd, *J* = 8.3, 1.3, 0.6 Hz, 1H), 7.83 – 7.75 (m, 2H), 7.72 (td, *J* = 7.7, 1.8 Hz, 1H), 7.53 (ddd, *J* = 8.2, 7.0, 1.3 Hz, 1H), 7.48 (s, 1H), 7.42 – 7.23 (m, 5H), 5.01 (d, *J* = 4.4 Hz, 2H), 3.02 (q, *J* = 7.5 Hz, 2H), 1.22 (t, *J* = 7.5 Hz, 3H).

*2-([1,1'-Biphenyl]-2-yl)-N-(pyridin-2-ylmethyl)quinazolin-4-amine (**25**).*

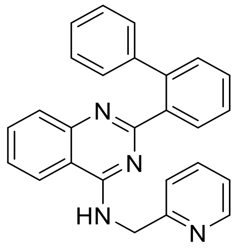


2-Chloro-*N*-(pyridin-2-ylmethyl)quinazolin-4-amine **49** (50 mg, 0.185 mmol) and biphenylboronic acid (73.2 mg, 0.369 mmol) were reacted according to general procedure 2 for 45 min at 120 °C. Upon cooling to room temperature the reaction mixture was loaded onto an SCX-II column, washed with MeOH, then eluted with 0.5 M $NH_3$ in MeOH and concentrated *in vacuo*. Purification *via* preparatory HPLC (gradient elution 5 to 95% MeCN in $H_2O$ with 0.1% $NH_3$) yielded 2-(2-phenylphenyl)-*N*-(pyridin-2-ylmethyl)quinazolin-4-amine **25** (55.0 mg, 0.142 mmol, 77%) as a white solid. MS (ESI+) *m/z* calcd for $C_{26}H_{21}N_4$ $[M + H]^+$ 389.2; found 389.1. UPLC (method C) $t_R$ = 5.34 min, >98%. $^1H$ NMR (300 MHz, Chloroform-*d*) δ 8.59 (dt, *J* = 4.9, 1.3 Hz, 1H), 8.18 – 8.06 (m, 1H), 7.88 (ddd, *J* = 20.8, 8.4, 1.2 Hz, 2H), 7.71 (dtd, *J* = 22.5, 7.3, 1.6 Hz, 2H), 7.59 – 7.41 (m, 4H), 7.38 – 7.30 (m, 2H), 7.30 – 7.08 (m, 6H), 4.14 (d, *J* = 4.2 Hz, 2H).

*2-(4-((Pyridin-2-ylmethyl)amino)quinazolin-2-yl)benzonitrile (**26**).*

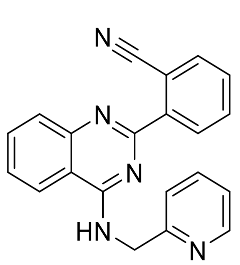


2-Chloro-*N*-(pyridin-2-ylmethyl)quinazolin-4-amine **49** (50 mg, 0.185 mmol) and 2-cyanophenylboronic acid (35.0 mg, 0.22 mmol) were reacted according to general procedure 3. Upon cooling to room temperature the

reaction mixture was purified directly *via* silica gel chromatography (gradient elution 10 to 80% EtOAc in petroleum ether) yielding 2-(4-((pyridin-2-ylmethyl)amino)quinazolin-2-yl)benzonitrile **26** (9.0 mg, 0.027 mmol, 15%) as a white solid. MS (ESI+) *m/z* calcd for $C_{21}H_{16}N_5$ $[M + H]^+$ 338.1; found 338.1. UPLC (method C) $t_R$ = 4.78 min, >98%. $^1$H NMR (300 MHz, Chloroform-*d*) δ 8.65 (ddd, *J* = 4.9, 1.8, 1.0 Hz, 1H), 8.60 (ddd, *J* = 8.0, 1.3, 0.5 Hz, 1H), 8.05 – 7.93 (m, 2H), 7.88 (ddd, *J* = 7.7, 1.4, 0.5 Hz, 1H), 7.84 – 7.79 (m, 1H), 7.77 – 7.66 (m, 3H), 7.60 – 7.52 (m, 2H), 7.52 – 7.46 (m, 1H), 7.33 – 7.21 (m, 1H, obsc. by solvent peak), 5.23 (d, *J* = 4.4 Hz, 2H).

*2-(2-(Dimethylamino)phenyl)-N-(pyridin-2-ylmethyl)quinazolin-4-amine (**27**).*

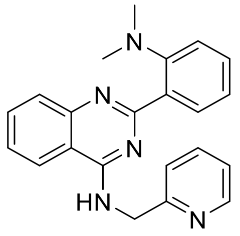

2-Chloro-*N*-(pyridin-2-ylmethyl)quinazolin-4-amine **49** (100 mg, 0.36 mmol) and N,N-dimethyl-2-(4,4,5,5-tetramethyl-1,3,2-dioxaborolan-2-yl)aniline (134 mg, 0.54 mmol) were reacted according to general procedure 2 overnight at 120 °C. Upon cooling to room temperature the reaction mixture was loaded onto an SCX-II column, washed with MeOH, then eluted with 0.5 M $NH_3$ in MeOH and concentrated *in vacuo*. Purification *via* preparatory HPLC (gradient elution 5 to 95% MeCN in $H_2O$ with 0.1% $NH_3$) yielded 2-(2-(dimethylamino)phenyl)-*N*-(pyridin-2-ylmethyl)quinazolin-4-amine **27** (3.0 mg, 0.008 mmol, 2.3%) as a white solid. MS (ESI+) *m/z* calcd for $C_{22}H_{22}N_5$ $[M + H]^+$ 356.2; found 356.1. UPLC (method D) $t_R$ = 3.18 min, >98%. $^1$H NMR (300 MHz, Chloroform-d) δ 8.69 – 8.61 (m, 1H), 8.01 – 7.91 (m, 2H), 7.80 – 7.74 (m, 1H), 7.74 – 7.65 (m, 2H), 7.51 (ddd, J = 8.2, 7.0, 1.3 Hz, 1H), 7.44 – 7.37 (m, 2H), 7.33 (ddd, J = 8.3, 7.2, 1.8 Hz, 1H), 7.26 (d, J = 6.5 Hz, 1H), 7.04 (dd, J = 8.3, 1.1 Hz, 1H), 6.98 (td, J = 7.4, 1.1 Hz, 1H), 5.01 (s, 2H), 2.73 (s, 6H).

*2-Chloro-N-(pyridin-2-yl)quinazolin-4-amine (**51**).*

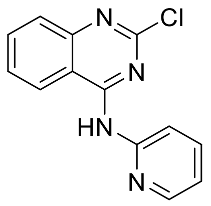


2,4-Dichloroquinazoline **43** (0.50 g, 2.51 mmol) and 2-aminopyridine (0.24 g, 2.51 mmol) were reacted according to general procedure 1 in $CHCl_3$ (10 mL) at 80ºC for 18 h. Purification *via* silica gel chromatography (gradient elution 5 to 100% EtOAc in petroleum ether) gave 2-chloro-*N*-(pyridin-2-yl)quinazolin-4-amine **51** (0.64 g, 2.49 mmol, 99%) as a white solid. MS (ESI+) *m/z* calcd for $C_{13}H_{10}ClN_4$ $[M + H]^+$ 257.1; found 257.2. UPLC (method A) $t_R$ = 2.95 min, 96%.

*2-(2-Isopropylphenyl)-N-(pyridin-2-yl)quinazolin-4-amine (**28**).*

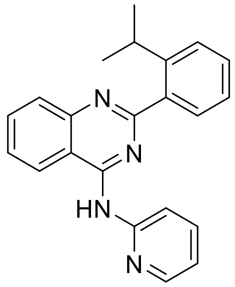


2-Chloro-*N*-(pyridin-2-yl)quinazolin-4-amine **51** (100 mg, 0.39 mmol) and (2-isopropylphenyl)boronic acid (96 mg, 0.58 mmol) were reacted according to general procedure 2 for 3.5 h at 130 °C. Upon cooling to room temperature the reaction mixture was loaded onto an SCX-II column, washed with MeOH, then eluted with 0.5 M $NH_3$ in MeOH and concentrated *in vacuo*. Purification *via* preparatory HPLC (gradient elution 5 to 95% MeCN in $H_2O$ with 0.1% $NH_3$) yielded 2-(2-isopropylphenyl)-*N*-(pyridin-2-yl)quinazolin-4-amine **28** (24 mg, 0.07 mmol, 19%) as a white solid. MS (ESI+) *m/z* calcd for $C_{22}H_{21}N_4$ $[M + H]^+$ 341.2; found 341.2. UPLC (method C) $t_R$ = 6.18 min, >98%. $^1H$ NMR (300 MHz, Chloroform-d) δ 8.88 – 8.78 (m, 0.7H), 8.65 (d, *J* = 7.9 Hz, 0.3H), 8.41 – 8.32 (m, 0.7H), 8.22 (d, *J* = 5.3 Hz, 0.3H), 8.05 (m, 1.3H), 7.88 (ddd, *J* = 8.2, 6.9, 1.3 Hz, 0.7H), 7.79 – 7.56 (m, 3H), 7.56 – 7.45 (m, 2H), 7.45 – 7.28 (m, 2H), 7.11 – 6.92 (m, 1H), 3.72 (p, *J* = 6.9 Hz, 0.7H), 3.53 – 3.39 (m, 0.3H), 1.29 (m, 6H), rotamers observed.

*2-Chloro-N-(1-(pyridin-2-yl)ethyl)quinazolin-4-amine (**52**).*

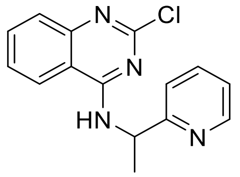


2,4-Dichloroquinazoline **43** (1.00 g, 5.02 mmol) and 1-(pyridin-2-yl)ethan-1-amine (0.68 g, 5.53 mmol) were reacted according to general procedure 1 for 18 h to give 2-chloro-*N*-(1-(pyridin-2-yl)ethyl)quinazolin-4-amine **52** (0.89 g, 3.12 mmol, 62%) as a cream coloured solid which was used without further purification. MS (ESI+) *m*/*z* calcd for $C_{15}H_{14}ClN_4$ $[M + H]^+$ 285.1; found 285.0. UPLC (method A) $t_R$ = 2.57 min, 68%.

*2-(2-Isopropylphenyl)-N-(1-(pyridin-2-yl)ethyl)quinazolin-4-amine (**29**).*

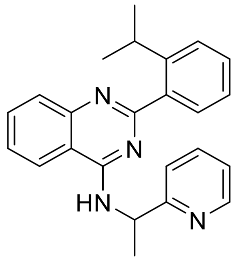


2-Chloro-*N*-(1-(pyridin-2-yl)ethyl)quinazolin-4-amine **52** (100 mg, 0.36 mmol) and (2-isopropylphenyl)boronic acid (89 mg, 0.54 mmol) were reacted according to general procedure 2 for 40 mins at 120 °C. Upon cooling to room temperature the reaction mixture was loaded onto an SCX-II column, washed with MeOH, then eluted with 0.5 M $NH_3$ in MeOH and concentrated *in vacuo*. Purification *via* preparatory HPLC (gradient elution 5 to 95% MeCN in $H_2O$ with 0.1% $NH_3$) yielded 2-(2-isopropylphenyl)-*N*-(1-(pyridin-2-yl)ethyl)quinazolin-4-amine **29** (50 mg, 0.14 mmol, 38%) as a white solid. MS (ESI+) *m*/*z* calcd for $C_{24}H_{25}N_4$ $[M + H]^+$ 369.2; found 369.2. UPLC (method D) $t_R$ = 4.05 min, >98%. 1H NMR (300 MHz, Chloroform-d) δ 8.66 (ddd, J = 4.9, 1.8, 0.9 Hz, 1H), 8.02 – 7.87 (m, 2H), 7.81 – 7.72 (m, 1H), 7.72 – 7.65 (m, 2H), 7.62 (d, J = 7.0 Hz, 1H), 7.52 (ddd, J = 8.2, 7.0, 1.3 Hz, 1H), 7.49 – 7.45 (m, 1H), 7.42 (td, J = 8.0, 7.4, 1.5 Hz, 1H), 7.34 – 7.29 (m, 2H), 7.26 (ddd, J = 7.5, 4.9, 1.1 Hz, 1H), 5.69 (p, J = 6.7 Hz, 1H), 3.76 – 3.60 (m, 1H), 1.66 (d, J = 6.7 Hz, 3H), 1.32 (d, J = 6.8 Hz, 3H), 1.26 (d, J = 6.9 Hz, 3H).

*2-Chloro-N-((5-(trifluoromethyl)pyridin-2-yl)methyl)quinazolin-4-amine (**53**).*

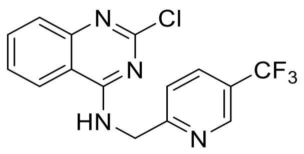

2,4-Dichloroquinazoline **43** (0.20 g, 1.00 mmol) and [5-(trifluoromethyl)pyridin-2-yl]methanamine hydrochloride (0.22 g, 1.06 mmol) were reacted according to general procedure 1 with 4 eq of triethylamine for 60 h to give 2-chloro-*N*-[[5-(trifluoromethyl)-2-pyridyl]methyl]quinazolin-4-amine **53** (0.19 g, 0.57 mmol, 57%) as a cream coloured solid which was used without further purification. MS (ESI+) *m*/*z* calcd for $C_{15}H_{11}ClF_3N_4$ [M + H]$^+$ 339.1; found 339.0. UPLC (method A) $t_R$ = 2.92 min, 91%.

*2-(2-Isopropylphenyl)-N-((5-(trifluoromethyl)pyridin-2-yl)methyl)quinazolin-4-amine (**30**).*

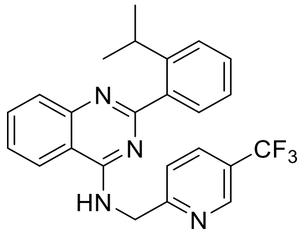

2-Chloro-*N*-((5-(trifluoromethyl)pyridin-2-yl)methyl)quinazolin-4-amine **53** (80 mg, 0.24 mmol) and (2-isopropylphenyl)boronic acid (58 mg, 0.35 mmol) were reacted according to general procedure 2 for 40 mins at 125 °C. Upon cooling to room temperature the reaction mixture was loaded onto an SCX-II column, washed with MeOH, then eluted with 0.5 M $NH_3$ in MeOH and concentrated *in vacuo*. Purification *via* preparatory HPLC (gradient elution 5 to 95% MeCN in $H_2O$ with 0.1% $NH_3$) yielded 2-(2-isopropylphenyl)-*N*-((5-(trifluoromethyl)pyridin-2-yl)methyl)quinazolin-4-amine **30** (31 mg, 0.07 mmol, 31%) as a white solid. MS (ESI+) *m*/*z* calcd for $C_{24}H_{22}F_3N_4$ [M + H]$^+$ 423.2; found 423.3. UPLC (method C) $t_R$ = 6.14 min, >98%. $^1$H NMR (300 MHz, Chloroform-d) δ 8.93 (dt, *J* = 2.0, 0.9 Hz, 1H), 8.02 – 7.89 (m, 3H), 7.81 (ddd, *J* = 8.2, 7.0, 1.4 Hz, 1H), 7.66 (ddd, *J* = 7.6, 1.4, 0.6 Hz, 1H), 7.56 (ddd, *J* = 8.3, 7.0, 1.2 Hz, 1H), 7.51 – 7.36 (m, 3H), 7.33-7.25 (m, 2H), 5.09 (d, *J* = 4.6 Hz, 2H), 3.58 (hept, *J* = 6.8 Hz, 1H), 1.25 (d, *J* = 6.9 Hz, 6H).

*2-(((2-Chloroquinazolin-4-yl)amino)methyl)pyridin-3-ol (**54**).*

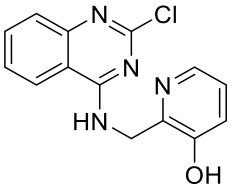


2,4-Dichloroquinazoline **43** (200 mg, 1.00 mmol) and 2-(aminomethyl)pyridin-3-ol dihydrochloride (208 mg, 1.06 mmol) were reacted according to general procedure 1 with 5 eq of triethylamine for 60 h to give 2-(((2-chloroquinazolin-4-yl)amino)methyl)pyridin-3-ol **54** (216 mg, 0.75 mmol, 75%) as a white solid which was used without further purification. MS (ESI+) *m/z* calcd for $C_{14}H_{12}ClN_4O$ $[M + H]^+$ 287.1; found 287.0. UPLC (method A) $t_R$ = 1.79 min, 32%.

*2-(((2-(2-Isopropylphenyl)quinazolin-4-yl)amino)methyl)pyridin-3-ol (**31**).*

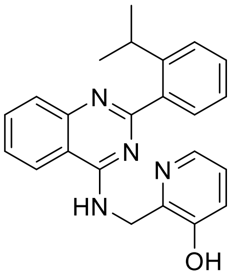


2-(((2-Chloroquinazolin-4-yl)amino)methyl)pyridin-3-ol **54** (80 mg, 0.28 mmol) and (2-isopropylphenyl)boronic acid (69 mg, 0.42 mmol) were reacted according to general procedure 2 for 40 mins at 125 °C. Upon cooling to room temperature the reaction mixture was loaded onto an SCX-II column, washed with MeOH, then eluted with 0.5 M $NH_3$ in MeOH and concentrated *in vacuo*. Purification *via* preparatory HPLC (gradient elution 5 to 95% MeCN in $H_2O$ with 0.1% $NH_3$) yielded 2-(((2-(2-isopropylphenyl)quinazolin-4-yl)amino)methyl)pyridin-3-ol **31** (8 mg, 0.02 mmol, 8%) as a white solid. MS (ESI+) *m/z* calcd for $C_{23}H_{23}N_4O$ [M + H]+ 371.2; found 371.3. UPLC (method C) $t_R$ = 4.03 min, >95%. $^1$H NMR (300 MHz, Chloroform-d) δ 11.18 (s, 1H), 8.08 (dd, *J* = 3.9, 2.1 Hz, 1H), 7.93 (dt, *J* = 8.2, 1.1 Hz, 1H), 7.82 (tdd, *J* = 8.3, 7.1, 1.1 Hz, 2H), 7.59 (dt, *J* = 7.4, 1.0 Hz, 1H), 7.55 – 7.41 (m, 4H), 7.35 (ddd, *J* = 7.6, 5.5, 3.1 Hz, 1H), 7.22 – 7.09 (m, 2H), 4.95 (d, *J* = 6.4 Hz, 2H), 3.45 – 3.29 (m, 1H), 1.28 (d, *J* = 6.8 Hz, 6H).

*6-Bromo-2-(2-isopropylphenyl)quinazolin-4-ol (**56**).*

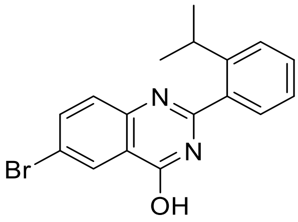

2-(Propan-2-yl)benzoic acid **55** (200 mg, 1.22mmol) was taken up in anhydrous DCM (5 mL) with 3 drops of anhydrous DMF, and cooled on an ice bath. Thionyl dichloride (0.34 mL, 4.65 mmol) was added dropwise and the mixture heated to 50 °C for 4 hours. The solvent was removed *in vacuo* and co-evaporated with toluene. The prepared acid chloride was taken up in DCM (1 mL) and added dropwise to a suspension of 2-amino-5-bromobenzamide (250 mg, 1.16mmol) and triethylamine (0.32 mL, 2.33 mmol) in DCM (5 mL) at 0 °C. After addition, the reaction was warmed to room temperature and left to stir overnight. DMF (2 mL) was added to achieve full dissolution and the reaction stirred at room temperature for a further 4 hours. EtOAc was added to the reaction mixture and washed with sat. aq. $NaHCO_3$ (x1), $H_2O$ (x1), 1M aq. HCl (x1) and brine (x1), then dried ($MgSO_4$), and solvent removed to give a brown residue. Purification *via* silica gel chromatography (gradient elution 0 to 40% EtOAc in petroleum ether) yielded the intermediate *N*-(4-bromo-2-carbamoylphenyl)-2-isopropylbenzamide. MS (ESI+) *m/z* calcd for $C_{17}H_{17}BrN_2O_2$ $[M + H]^+$ 361.1; found 361.2. This material was taken up in 5% NaOH (217 mg, 5.42 mmol) in $H_2O$ (4.7 mL) and heated to reflux for 3 hours. The reaction was cooled and acidified to ~pH 5 with acetic acid. The resulting white solid was filtered and washed with $H_2O$ and dried under high vacuum to afford 6-bromo-2-(2-isopropylphenyl)quinazolin-4-ol **56** (350 mg, 1.02 mmol, 88%) as an off-white solid. MS (ESI+) *m/z* calcd for $C_{17}H_{15}BrN_2O$ $[M + H]^+$ 343.0; found 343.1. UPLC (method A) $t_R$ = 2.82 min, 97%. $^1$H NMR (300 MHz, Chloroform-*d*) δ 8.21 (s, 1H), 7.80 (d, *J* = 9.0 Hz, 1H), 7.64 (d, *J* = 8.8 Hz, 1H), 7.40 (d, *J* = 4.2 Hz, 2H), 7.36-7.27 (m, 2H, obsc. by solvent peak), 7.20-7.11 (m, 1H), 3.27 (p, *J* = 6.8 Hz, 1H), 1.18 (d, *J* = 6.8 Hz, 6H).

*6-Bromo-4-chloro-2-(2-isopropylphenyl)quinazoline (**57**).*

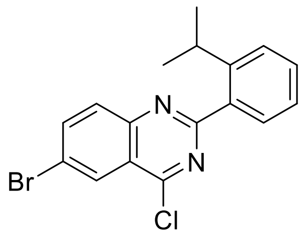

6-Bromo-2-(2-isopropylphenyl)quinazolin-4-ol **56** (380 mg, 1.11 mmol) was taken up in toluene (5 mL) and phosphorus oxychloride (3.5 mL, 37.3 mmol) was added. The mixture was heated for 18 hours at 90 °C. Reaction cooled and added slowly to cooled sat. aq. $NaHCO_3$, stirred for 30 mins on ice and extracted with EtOAc (2 ×), dried ($MgSO_4$) and solvent removed to give 6-bromo-4-chloro-2-(2-isopropylphenyl)quinazoline **57** (356 mg, 0.984 mmol, 89%) of an oil that solidified on standing. Not purified further. MS (ESI+) *m*/*z* calcd for $C_{17}H_{14}BrClN_2$ $[M + H]^+$ 361.0; found 361.1. UPLC (method A) $t_R$ = 3.97 min, 95%. $^1$H NMR (300 MHz, Chloroform-*d*) δ 8.49 (dd, *J* = 2.1, 0.6 Hz, 1H), 8.06 (dd, *J* = 8.9, 2.1 Hz, 1H), 8.00 (d, *J* = 8.9 Hz, 1H), 7.82 – 7.72 (m, 1H), 7.56 – 7.43 (m, 2H), 7.34 (ddd, *J* = 7.7, 6.1, 2.6 Hz, 1H), 3.59 (hept, *J* = 6.8 Hz, 1H), 1.30 (d, *J* = 6.8 Hz, 6H).

*6-Bromo-2-(2-isopropylphenyl)-N-(pyridin-2-ylmethyl)quinazolin-4-amine (**32**).*

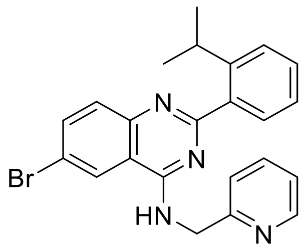

6-Bromo-4-chloro-2-(2-isopropylphenyl)quinazoline **57** (350 mg, 0.970 mmol) and pyridin-2-ylmethanamine (0.12 mL, 1.16 mmol) were reacted according to general procedure 1 for 18 hours. A further 0.5 eq of pyridin-2-ylmethanamine (0.05 mL, 0.48 mmol) was added and the reaction stirred at room temperature for a further 3 hours. The reaction was diluted with $CH_2Cl_2$, washed with brine (2 ×), then dried ($MgSO_4$) and concentrated *in vacuo*. 50 mg of crude material was purified *via* preparatory HPLC (gradient elution 40 to 80% MeCN in $H_2O$ with 0.1% $NH_3$) yielding 6-bromo-2-(2-isopropylphenyl)-*N*-(pyridin-2-ylmethyl)quinazolin-4-amine **32** (29.5 mg, 0.068 mmol, 7%) to be used for biological testing. MS (ESI+) *m*/*z* calcd for $C_{23}H_{22}BrN_4$ $[M + H]^+$ 433.1; found

433.2. UPLC (method C) $t_R$ = 6.16 min, >98%. $^1$H NMR (300 MHz, Chloroform-d) δ 8.68 (ddd, *J* = 5.0, 1.8, 1.0 Hz, 1H), 8.13 (dd, *J* = 1.9, 0.7 Hz, 1H), 7.85 (dd, *J* = 8.9, 1.9 Hz, 1H), 7.81 (dd, *J* = 8.9, 0.7 Hz, 1H), 7.73 (td, *J* = 7.7, 1.8 Hz, 1H), 7.68 (ddd, *J* = 7.6, 1.5, 0.6 Hz, 1H), 7.52 (s, 1H), 7.49 – 7.38 (m, 2H), 7.37 – 7.24 (m, 3H), 4.97 (d, *J* = 4.3 Hz, 2H), 3.60 (hept, *J* = 6.9 Hz, 1H), 1.28 (d, *J* = 6.9 Hz, 6H).

The remaining crude material was purified *via* silica gel chromatography (gradient elution 0 to 100% EtOAc in petroleum ether) yielding 6-bromo-2-(2-isopropylphenyl)-*N*-(pyridin-2-ylmethyl)quinazolin-4-amine **32** (0.26 g, 0.60 mmol, 62%) as a white solid for use as an intermediate. MS (ESI+) *m/z* 433 and 435 $[M + H]^+$. Combined yield (0.29g, 0.67 mmol, 69%).

*2-(2-Isopropylphenyl)-N⁴-(pyridin-2-ylmethyl)quinazoline-4,6-diamine (**33**).*

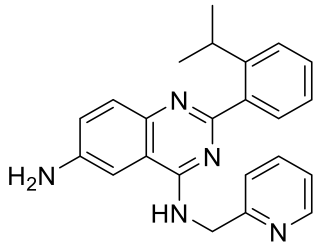


6-Bromo-2-(2-isopropylphenyl)-*N*-(pyridin-2-ylmethyl)quinazolin-4-amine **32** (30 mg, 0.070 mmol, 1.0 eq) was dissolved in EtOH:$H_2O$ 7:3 (1 mL) in a MW vial and sodium azide (9.0 mg, 0.14 mmol, 2.0 eq.) and copper(I) iodide (1.3 mg, 0.010 mmol, 0.1 eq.) were added, followed by sodium ascorbate (0.8 mg, 0.004 mmol, 0.06 eq.). The mixture was thoroughly degassed and *trans-N,N'*-dimethylcyclohexane-1,2-diamine (0.002 mL, 0.01 mmol, 0.2 eq.) was added followed by capping. Further degassing (including cooling and evacuating the vessel, and rewarming to rt, repeat) and the mixture was heated to 100 °C overnight. LCMS suggested ~40% conversion to azide and ~25% conversion to amine. The reaction mixture was loaded onto an SCX-2 column and washed through with MeOH. Product eluted with 0.5 M $NH_3$ in MeOH and solvent removed to give crude material which was purified *via* preparatory HPLC (gradient elution 30 to 70% MeCN in $H_2O$ with 0.1% $NH_3$) to give 2-(2-isopropylphenyl)-$N^4$-(pyridin-2-ylmethyl)quinazoline-4,6-diamine **33** (2.5 mg, 0.0068 mmol, 9.8%) as a pale brown solid. MS (ESI+) *m/z* calcd for $C_{23}H_{24}N_5$ $[M + H]^+$ 370.2; found 370.3. UPLC (method D) $t_R$ = 3.63 min, >98%. $^1$H NMR (300 MHz, Chloroform-d) δ 8.65 (dt, *J* = 5.0, 1.3 Hz, 1H), 7.79 (d, *J* = 8.8 Hz, 1H), 7.71 (td, *J* = 7.7, 1.8 Hz, 1H), 7.65 (dd, *J* = 7.5, 1.5 Hz, 1H), 7.44 (dd, *J* = 7.8, 1.7 Hz, 1H), 7.39 (td, *J* = 8.0, 7.5, 1.6 Hz, 1H),

7.34 (d, *J* = 7.8 Hz, 1H), 7.31 – 7.24 (m, 2H), 7.21 (dd, *J* = 8.8, 2.4 Hz, 1H), 7.11 (s, 1H), 7.08 (d, *J* = 2.5 Hz, 1H), 4.97 (d, *J* = 4.4 Hz, 2H), 4.01 (s, 2H), 3.61 (hept, *J* = 6.9 Hz, 1H), 1.26 (d, *J* = 6.9 Hz, 6H).

*2-Chloro-5-methyl-N-(pyridin-2-ylmethyl)pyrimidin-4-amine (**59**).*

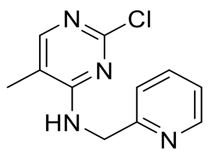


2,4-Dichloro-5-methylpyrimidine **58** (0.10 g, 0.62 mmol) and pyridin-2-ylmethanamine (77 µL, 80 mg, 0.74 mmol) were reacted according to general procedure 1 for 18 hours, followed by purification *via* silica gel chromatography (gradient elution 20 to 100% EtOAc in petroleum ether) to give 2-chloro-5-methyl-*N*-(pyridin-2-ylmethyl)pyrimidin-4-amine **59** (97 mg, 0.41 mmol, 67%) as a white solid. MS (ESI+) *m/z* calcd for $C_{11}H_{11}ClN_4$ $[M + H]^+$ 235.1; found 235.1. UPLC (method A) $t_R$ = 2.30 min, 97%.

*2-(2-Isopropylphenyl)-5-methyl-N-(pyridin-2-ylmethyl)pyrimidin-4-amine (**34**).*

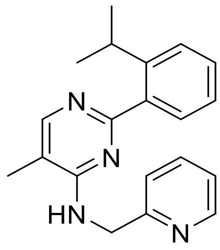


2-Chloro-5-methyl-*N*-(pyridin-2-ylmethyl)pyrimidin-4-amine **59** (45.2 mg, 0.193 mmol) and (2-propan-2-ylphenyl)boronic acid (47.4 mg, 0.289 mmol) were reacted according to general procedure 2 for 45 min at 120 °C, resulting in partial conversion to desired product. A further 0.5 equivalents of (2-propan-2-ylphenyl)boronic acid (12.0 mg, 0.072 mmol) and 0.1 equivalents of tetrakis(triphenylphosphine)palladium (22.2 mg, 0.019 mmol) were added to the reaction mixture with degassing and further heating for 30 min at 120 °C. Upon cooling to rt the reaction mixture was loaded onto an SCX-II column, washed with MeOH, then eluted with 0.5 M $NH_3$ in MeOH and concentrated *in vacuo*, yielding 2-(2-isopropylphenyl)-5-methyl-*N*-(pyridin-2-ylmethyl)pyrimidin-4-amine **34** (55.3 mg, 0.174 mmol, 90%) as a white solid. MS (ESI+) *m/z* calcd for $C_{20}H_{23}N_4^+$ $[M + H]^+$ 319.1917; found 319.2. HRMS (ESI+) *m/z* calcd for $C_{20}H_{23}N_4^+$ $[M + H]^+$ 319.1917; found 319.1915. UPLC (method C) $t_R$ = 4.89 min, 97%. $^1$H NMR (300 MHz, Chloroform-*d*) δ 8.61 (ddd, *J* = 4.9, 1.8, 1.0 Hz, 1H), 8.17 (d, *J* = 1.0 Hz, 1H), 7.74 – 7.63 (m, 1H), 7.63 – 7.57 (m, 1H), 7.57 – 7.43 (m, 1H), 7.43 – 7.34 (m, 2H), 7.33 – 7.20 (m, 2H), 6.27 (s, 1H), 4.87 (d, *J* = 4.6 Hz, 2H), 3.57 (h, *J* = 6.8 Hz, 1H), 2.23 (d, *J* = 0.9 Hz, 3H), 1.24 (d, *J* = 6.9 Hz, 6H).

*5-Methyl-N-(pyridin-2-ylmethyl)-2-(2-(trifluoromethyl)phenyl)pyrimidin-4-amine (**35**).*

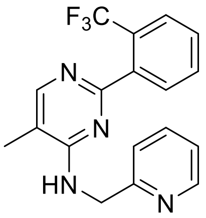

2-Chloro-5-methyl-*N*-(pyridin-2-ylmethyl)pyrimidin-4-amine **59** (45.2 mg, 0.193 mmol) and (2-trifluoromethyl)phenylboronic acid (54.9 mg, 0.289 mmol) were reacted according to general procedure 2 for 45 min at 120 °C. Upon cooling to room temperature the reaction mixture was loaded onto an SCX-II column, washed with MeOH, then eluted with 0.5 M $NH_3$ in MeOH and concentrated *in vacuo* yielding 5-methyl-*N*-(pyridin-2-ylmethyl)-2-[2-(trifluoromethyl)phenyl]pyrimidin-4-amine **35** (60.0 mg, 0.174 mmol, 90.5%) as a white solid. MS (ESI+) *m*/*z* calcd for $C_{18}H_{16}F_3N_4$ $[M + H]^+$ 345.1; found 345.1. HRMS (ESI+) *m*/*z* calcd for $C_{18}H_{16}F_3N_4^+$ $[M + H]^+$ 345.1322; found 345.1323. UPLC (method C) $t_R$ = 4.56 min, 98%. $^1$H NMR (300 MHz, DMSO-$d_6$) δ 8.49 (ddd, *J* = 4.8, 1.9, 1.0 Hz, 1H), 8.10 (d, *J* = 1.0 Hz, 1H), 7.80 – 7.51 (m, 6H), 7.29 – 7.18 (m, 2H), 4.73 (d, *J* = 5.8 Hz, 2H), 2.15 (d, *J* = 0.9 Hz, 3H). $^{13}$C NMR (75 MHz, DMSO-$d_6$) δ 163.0, 160.9, 159.6, 153.8, 149.2, 139.9, 136.9, 132.3, 131.6, 129.2, 127.3 (q, *J* = 30.6 Hz), 126.8 (q, *J* = 5.4 Hz), 122.3, 121.1, 112.8, 45.7, 14.0. $^{19}$F NMR (282 MHz, DMSO-$d_6$) δ -56.08.

*2-Chloro-N-(pyridin-2-ylmethyl)pyrido[3,2-d]pyrimidin-4-amine (**62**).*

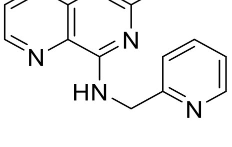

2,4-Dichloropyrido[3,2-d]pyrimidine **60** (0.15 g, 0.75 mmol) and pyridin-2-ylmethanamine (81 µL, 85 mg, 0.79 mmol) were reacted according to general procedure 1 for 18 hours, followed by purification *via* silica gel chromatography (gradient elution 20 to 100% EtOAc in petroleum ether) to give 2-chloro-*N*-(pyridin-2-ylmethyl)pyrido[3,2-d]pyrimidin-4-amine **62** (169 mg, 0.62 mmol, 83%) as a pale yellow solid. MS (ESI+) *m/z* calcd for $C_{13}H_{10}ClN_5$ $[M + H]^+$ 272.1; found 272.0. UPLC (method A) $t_R$ = 2.52 min, >98%. $^1$H NMR (300 MHz,

Chloroform-*d*) δ 8.77 (dd, *J* = 4.3, 1.6 Hz, 1H), 8.69 (ddd, *J* = 4.9, 1.8, 1.0 Hz, 1H), 8.65 (brs, 1H), 8.06 (dd, *J* = 8.5, 1.5 Hz, 1H), 7.74 (td, *J* = 7.8, 4.3 Hz, 1H), 7.69 (dd, *J* = 8.5, 4.3 Hz, 1H), 7.39 (dt, *J* = 7.9, 1.0 Hz, 1H), 7.34 – 7.23 (m, 1H, obsc. by solvent peak), 4.98 (d, *J* = 5.0 Hz, 2H).

*2-(2-Isopropylphenyl)-N-(pyridin-2-ylmethyl)pyrido[3,2-d]pyrimidin-4-amine (**36**).*

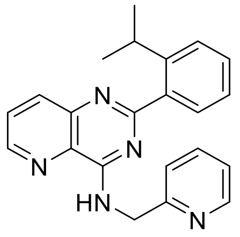


2-Chloro-*N*-(pyridin-2-ylmethyl)pyrido[3,2-d]pyrimidin-4-amine **62** (50.0 mg, 0.181 mmol) and (2-propan-2-ylphenyl)boronic acid (59.3 mg, 0.361 mmol) were reacted according to general procedure 2 for 45 min at 120 °C, resulting in partial conversion to desired product. A further 2 equivalents of (2-propan-2-ylphenyl)boronic acid (59.3 mg, 0.36 mmol) and 0.1 equivalents of tetrakis(triphenylphosphine)palladium (20.9 mg, 0.018 mmol) were added to the reaction mixture with degassing and further heating for 30 min at 120 °C. The reaction mixture was loaded onto an SCX-II column, washed with MeOH, then eluted with 0.5 M $NH_3$ in MeOH and concentrated *in vacuo*. Purification *via* preparatory HPLC (gradient elution 5 to 95% MeCN in $H_2O$ with 0.1% $NH_3$) yielded 2-(2-isopropylphenyl)-*N*-(pyridin-2-ylmethyl)pyrido[3,2-d]pyrimidin-4-amine **36** (44.2 mg, 0.124 mmol, 68%) as a beige solid. MS (ESI+) *m*/*z* calcd for $C_{22}H_{22}N_5$ $[M + H]^+$ 356.2; found 356.1. UPLC (method C) $t_R$ = 5.02 min, >98%. $^1$H NMR (300 MHz, Chloroform-d) δ 8.79 (ddd, *J* = 4.2, 1.6, 0.7 Hz, 1H), 8.67 (ddd, *J* = 4.9, 1.8, 0.9 Hz, 1H), 8.26 (s, 1H), 8.19 (ddd, *J* = 8.5, 1.6, 0.7 Hz, 1H), 7.76 – 7.62 (m, 3H), 7.49 – 7.33 (m, 3H), 7.33 – 7.20 (m, 2H), 5.04 (d, *J* = 5.4 Hz, 2H), 3.59 (dq, *J* = 13.7, 6.8 Hz, 1H), 1.25 (dd, *J* = 6.9, 0.7 Hz, 6H).

*2-Chloro-N-(pyridin-2-ylmethyl)pyrido[2,3-d]pyrimidin-4-amine (**63**).*

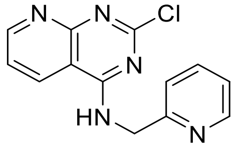

2,4-Dichloropyrido[2,3-d]pyrimidine **61** (0.25 g, 1.25 mmol) and pyridin-2-ylmethanamine (0.15 g, 1.37 mmol) were reacted according to general procedure 1 for 18 hours to give 2-chloro-*N*-(pyridin-2-ylmethyl)pyrido[2,3-d]pyrimidin-4-amine **63** (125 mg, 0.83 mmol, 66%) as a pale yellow solid which was used without further purification. MS (ESI+) *m/z* calcd for $C_{13}H_{10}ClN_5$ $[M + H]^+$ 272.1; found 272.1. UPLC (method A) $t_R$ = 2.20 min, >95%. $^1$H NMR (300 MHz, Chloroform-*d*) δ 9.08 (dd, *J* = 4.4, 1.8 Hz, 1H), 8.69 – 8.60 (m, 1H), 8.34 (dd, *J* = 8.2, 1.9 Hz, 1H), 8.16 (s, 1H), 7.78 (td, *J* = 7.7, 1.8 Hz, 1H), 7.48 (dd, *J* = 8.2, 4.4 Hz, 1H), 7.40 (dt, *J* = 7.9, 1.0 Hz, 1H), 7.36 – 7.29 (m, 1H), 4.95 (d, *J* = 4.1 Hz, 2H).

*2-(2-Isopropylphenyl)-N-(pyridin-2-ylmethyl)pyrido[2,3-d]pyrimidin-4-amine (**37**).*

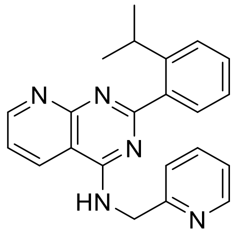


2-Chloro-*N*-(pyridin-2-ylmethyl)pyrido[2,3-d]pyrimidin-4-amine **63** (50 mg, 0.184 mmol) and (2-propan-2-ylphenyl)boronic acid (45.3 mg, 0.276 mmol) were reacted according to general procedure 2 for 45 mins at 120 °C. Upon cooling to room temperature the reaction mixture was loaded onto an SCX-II column, washed with MeOH, then eluted with 0.5 M $NH_3$ in MeOH and concentrated *in vacuo*. Purification *via* preparatory HPLC (gradient elution 5 to 95% MeCN in $H_2O$ with 0.1% $NH_3$) yielded 2-(2-isopropylphenyl)-*N*-(pyridin-2-ylmethyl)pyrido[2,3-d]pyrimidin-4-amine **37** (17.7 mg, 0.05 mmol, 27% yield) as a white solid. MS (ESI+) *m/z* calcd for $C_{22}H_{22}N_5$ $[M + H]^+$ 356.2; found 356.2. HRMS (ESI+) *m/z* calcd for $C_{22}H_{22}N_5^+$ $[M + H]^+$ 356.1870; found 356.1869. UPLC (method C) $t_R$ = 5.28 min, >98%. $^1$H NMR (300 MHz, Chloroform-d) δ 9.12 (dd, *J* = 4.4, 1.9 Hz, 1H), 8.65 (dt, *J* = 4.8, 1.4 Hz, 1H), 8.38 (dd, *J* = 8.2, 1.9 Hz, 1H), 7.90 – 7.80 (m, 1H), 7.78 – 7.68 (m, 2H), 7.49 – 7.39 (m, 3H), 7.35 (dt, *J* = 7.8, 1.0 Hz, 1H), 7.33 – 7.26 (m, 2H), 4.99 (d, *J* = 4.3 Hz, 2H), 3.86 (dq, *J* = 13.7, 6.9 Hz, 1H), 1.31 (d, *J* = 6.9 Hz, 6H).

*N-(Pyridin-2-ylmethyl)-2-[2-(trifluoromethyl)phenyl]pyrido[2,3-d]pyrimidin-4-amine (**38**).*

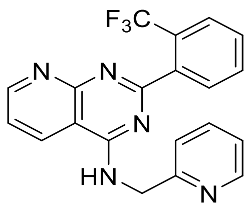

2-Chloro-*N*-(pyridin-2-ylmethyl)pyrido[2,3-d]pyrimidin-4-amine **63** (50 mg, 0.184 mmol) and (2-trifluoromethyl)phenylboronic acid (52.4 mg, 0.276 mmol) were reacted according to general procedure 2 for 45 mins at 120 °C. Upon cooling to room temperature the reaction mixture was loaded onto an SCX-II column, washed with MeOH, then eluted with 0.5 M $NH_3$ in MeOH and concentrated *in vacuo*. Purification *via* preparatory HPLC (gradient elution 5 to 95% MeCN in $H_2O$ with 0.1% $NH_3$) yielded *N*-(pyridin-2-ylmethyl)-2-[2-(trifluoromethyl)phenyl]pyrido[2,3-d]pyrimidin-4-amine **38** (26.3 mg, 0.69 mmol, 37.5% yield) as a white solid. MS (ESI+) *m/z* calcd for $C_{20}H_{15}F_3N_5$ $[M + H]^+$ 382.1; found 382.2. HRMS (ESI+) *m/z* calcd for $C_{20}H_{15}F_3N_5^+$ $[M + H]^+$ 382.1274; found 382.1275. UPLC (method D) $t_R$ = 4.17 min, >98%. $^1H$ NMR (300 MHz, Chloroform-d) δ 9.12 (dd, J = 4.4, 1.9 Hz, 1H), 8.69 – 8.60 (m, 1H), 8.40 (dd, J = 8.2, 1.9 Hz, 1H), 8.10 – 8.01 (m, 1H), 7.88 (s, 1H), 7.83 (dd, J = 7.8, 1.3 Hz, 1H), 7.74 (td, J = 7.7, 1.8 Hz, 1H), 7.67 (dd, J = 8.3, 6.9 Hz, 1H), 7.63 – 7.54 (m, 1H), 7.49 (dd, J = 8.2, 4.4 Hz, 1H), 7.39 – 7.34 (m, 1H), 7.32-7.26 (m, 1H), 4.99 (d, J = 4.3 Hz, 2H). $^{13}C$ NMR (75 MHz, DMSO-$d_6$) δ 165.3, 161.2, 159.0, 158.6, 156.6, 149.4, 139.9 (q, *J* = 2.0 Hz), 137.1, 133.1, 132.5, 131.7, 129.6, 127.4 (q, *J* = 30.7 Hz), 126.9 (q, *J* = 5.1 Hz), 124.6 (q, *J* = 273.6 Hz), 122.6, 122.2, 121.5, 108.6, 46.2. $^{19}F$ NMR (282 MHz, DMSO-$d_6$) δ -56.02.

*2,4-Dichloro-7-methyl-7H-pyrrolo[2,3-d]pyrimidine (**65**).*

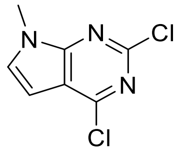

To a stirred solution of 2,4-dichloro-7*H*-pyrrolo[2,3-d]pyrimidine **64** (1.00 g, 5.32mmol) in DMF (8 mL) at room temperature was added sodium hydride (60% dispersion in mineral oil) (0.23 g, 5.85 mmol) in one portion and the reaction allowed to stir for 20 minutes before iodomethane (0.40 mL, 0.91 g, 6.38 mmol) was added *via* syringe and the reaction stirred at room temperature overnight. The reaction was diluted with water and EtOAc, extracted into EtOAc (x3), dried over sodium sulfate, filtered and concentrated *in vacuo*. Purification *via* silica gel

chromatography (gradient elution 5 to 100% EtOAc in petroleum ether) yielded 2,4-dichloro-7-methyl-7*H*-pyrrolo[2,3-d]pyrimidine **65** (1.01 g, 5.00 mmol, 94%) as a white solid. MS (ESI+) *m/z* calcd for $C_7H_6Cl_2N_3$ [M + H]$^+$ 202.0; not observed. UPLC (method A) $t_R$ = 2.72 min, 89%.

*2-Chloro-7-methyl-N-(pyridin-2-ylmethyl)-7H-pyrrolo[2,3-d]pyrimidin-4-amine (**66**).*

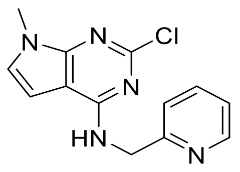


2,4-Dichloro-7-methyl-7*H*-pyrrolo[2,3-d]pyrimidine **65** (255 mg, 1.26 mmol) and pyridin-2-ylmethanamine (143 mg, 1.33 mmol) were reacted according to general procedure 1 in chloroform (8 mL) at 80 °C for 18 h. Purification *via* silica gel chromatography (gradient elution 5 to 100% EtOAc in petroleum ether) gave 2-Chloro-7-methyl-N-(pyridin-2-ylmethyl)-7H-pyrrolo[2,3-d]pyrimidin-4-amine **66** (238 mg, 0.87 mmol, 69%) as a white solid. MS (ESI+) *m/z* calcd for $C_{13}H_{13}ClN_5$ [M + H]$^+$ 274.1; found 274.1. UPLC (method A) $t_R$ = 2.52 min, 73%.

*2-(2-Isopropylphenyl)-7-methyl-N-(pyridin-2-ylmethyl)-7H-pyrrolo[2,3-d]pyrimidin-4-amine (**39**).*

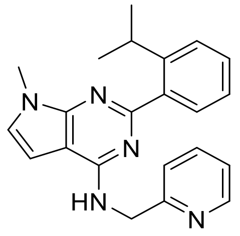


2-Chloro-7-methyl-N-(pyridin-2-ylmethyl)-7H-pyrrolo[2,3-d]pyrimidin-4-amine **66** (60 mg, 0.22 mmol) and (2-isopropylphenyl)boronic acid (54 mg, 0.33 mmol) were reacted according to general procedure 2 for 1 h at 125 °C. Upon cooling to room temperature the reaction mixture was loaded onto an SCX-II column, washed with MeOH, then eluted with 0.5 M $NH_3$ in MeOH and concentrated *in vacuo*. Purification *via* preparatory HPLC (gradient elution 5 to 95% MeCN in $H_2O$ with 0.1% $NH_3$) yielded 2-(2-isopropylphenyl)-5-methyl-*N*-(pyridin-2-ylmethyl)-5*H*-pyrrolo[3,2-d]pyrimidin-4-amine **39** (51 mg, 0.14 mmol, 65%) as a white solid. MS (ESI+) *m/z*

calcd for $C_{22}H_{24}N_5$ $[M + H]^+$ 358.2; found 358.3. UPLC (method D) $t_R$ = 4.21 min, >98%. $^1$H NMR (300 MHz, Chloroform-d) δ 8.62 (ddd, *J* = 4.9, 1.8, 1.0 Hz, 1H), 7.74 – 7.61 (m, 2H), 7.49 – 7.17 (m, 6H), 6.96 (d, *J* = 3.5 Hz, 1H), 6.48 (d, *J* = 3.5 Hz, 1H), 6.25 (t, *J* = 5.2 Hz, 1H), 5.00 (d, *J* = 5.2 Hz, 2H), 3.85 (s, 3H), 3.65 (p, *J* = 6.9 Hz, 1H), 1.26 (d, *J* = 6.9 Hz, 6H). 1H NMR (300 MHz, Chloroform-d) δ 8.62 (ddd, J = 4.9, 1.8, 1.0 Hz, 1H), 7.74 – 7.61 (m, 2H), 7.44 (dd, J = 7.9, 1.6 Hz, 1H), 7.38 (ddd, J = 8.0, 7.0, 1.5 Hz, 2H), 7.31 – 7.24 (m, 1H), 7.24 – 7.19 (m, 1H), 6.96 (d, J = 3.5 Hz, 1H), 6.48 (d, J = 3.5 Hz, 1H), 6.25 (t, J = 5.2 Hz, 1H), 5.00 (d, J = 5.2 Hz, 2H), 3.85 (s, 3H), 3.65 (p, J = 6.9 Hz, 1H), 1.26 (d, J = 6.9 Hz, 6H).

*2,4-Dichloro-5-methyl-5H-pyrrolo[3,2-d]pyrimidine (**68**).*

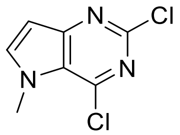

To a stirred solution of 2,4-dichloro-5*H*-pyrrolo[3,2-d]pyrimidine **67** (1.00 g, 5.32mmol) in DMF (10 mL) at room temperature was added sodium hydride (60% dispersion in mineral oil) (0.23 g, 5.85 mmol) in one portion and the reaction allowed to stir for 20 minutes before iodomethane (0.40 mL, 0.91 g, 6.38 mmol) was added *via* syringe and the reaction stirred at room temperature for 2 h. The reaction was diluted with water and EtOAc, extracted into EtOAc (x3), dried over sodium sulfate, filtered and concentrated *in vacuo*. Purification *via* silica gel chromatography (gradient elution 5 to 100% EtOAc in petroleum ether) yielded 2,4-Dichloro-5-methyl-5H-pyrrolo[3,2-d]pyrimidine **68** (0.98 g, 4.83 mmol, 91%) as a white solid. MS (ESI+) *m/z* calcd for $C_7H_6Cl_2N_3$ $[M + H]^+$ 202.0; found 202.0 (50%). UPLC (method A) $t_R$ = 2.47 min, 89%.

*2-Chloro-5-methyl-N-(pyridin-2-ylmethyl)-5H-pyrrolo[3,2-d]pyrimidin-4-amine (**71**).*

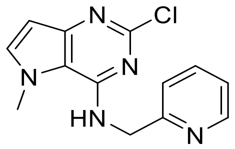

2,4-Dichloro-5-methyl-5H-pyrrolo[3,2-d]pyrimidine **68** (0.98 g, 4.83 mmol) and pyridin-2-ylmethanamine (0.55 g, 5.07 mmol) were reacted according to general procedure 1 in chloroform (12 mL) at reflux overnight to give 2-chloro-5-methyl-*N*-(pyridin-2-ylmethyl)-5*H*-pyrrolo[3,2-d]pyrimidin-4-amine **71** (0.97 g, 3.54 mmol, 73%) as a white solid which was used without further purification. MS (ESI+) *m/z* calcd for $C_{13}H_{13}ClN_5$ $[M + H]^+$ 274.1; found 274.0. UPLC (method B) $t_R$ = 1.91 min, >90%.

*2-(2-Isopropylphenyl)-5-methyl-N-(pyridin-2-ylmethyl)-5H-pyrrolo[3,2-d]pyrimidin-4-amine (**40**).*

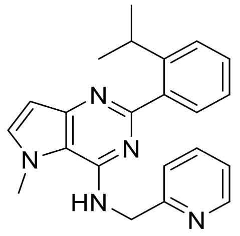


2-Chloro-5-methyl-*N*-(pyridin-2-ylmethyl)-5*H*-pyrrolo[3,2-d]pyrimidin-4-amine **71** (100 mg, 0.37 mmol) and (2-isopropylphenyl)boronic acid (90 mg, 0.55 mmol) were reacted according to general procedure 2 for 40 mins at 125 °C. Upon cooling to room temperature the reaction mixture was loaded onto an SCX-II column, washed with MeOH, then eluted with 0.5 M $NH_3$ in MeOH and concentrated *in vacuo*. Purification *via* preparatory HPLC (gradient elution 5 to 95% MeCN in $H_2O$ with 0.1% $NH_3$) yielded 2-(2-isopropylphenyl)-5-methyl-*N*-(pyridin-2-ylmethyl)-5*H*-pyrrolo[3,2-d]pyrimidin-4-amine **40** (36 mg, 0.10 mmol, 28%) as a white solid. MS (ESI+) *m/z* calcd for $C_{22}H_{24}N_5$ $[M + H]^+$ 358.2; found 358.1. HRMS (ESI+) *m/z* calcd for $C_{22}H_{24}N_5^+$ $[M + H]^+$ 358.2026; found 358.2033. UPLC (method C) $t_R$ = 4.87 min, >98%. $^1H$ NMR (300 MHz, DMSO-$d_6$) δ 8.51 (ddd, *J* = 4.9, 1.8, 0.9 Hz, 1H), 7.72 (td, *J* = 7.7, 1.8 Hz, 1H), 7.47 (d, *J* = 3.0 Hz, 1H), 7.45 – 7.19 (m, 6H), 7.13 (ddd, *J* = 7.6, 5.2, 3.4 Hz, 1H), 6.37 (d, *J* = 3.0 Hz, 1H), 4.83 (d, *J* = 5.6 Hz, 2H), 4.16 (s, 3H), 3.44 (p, *J* = 6.9 Hz, 1H), 0.92 (d, *J* = 6.9 Hz, 6H). $^{13}C$ NMR (75 MHz, DMSO-$d_6$) δ 160.0, 159.1, 149.8, 149.6, 149.2, 146.9, 140.4, 136.9, 134.1, 130.4, 128.2, 125.5, 125.2, 122.2, 121.0, 113.8, 101.1, 45.7, 37.0, 28.7, 24.2.

*2,4-Dichloro-5-(cyclopropylmethyl)-5H-pyrrolo[3,2-d]pyrimidine (**69**).*

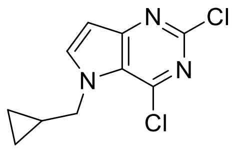

To a stirred solution of 2,4-dichloro-5*H*-pyrrolo[3,2-d]pyrimidine **67** (1.00 g, 5.32mmol) in DMF (10 mL) at room temperature was added sodium hydride (60% dispersion in mineral oil) (0.23 g, 5.85 mmol) in one portion and the reaction allowed to stir for 20 minutes before (bromomethyl)cyclopropane (0.62 mL, 0.86 g, 6.38 mmol) was added *via* syringe and the reaction stirred at room temperature overnight. The reaction was diluted with water and EtOAc, extracted into EtOAc (x3), dried over sodium sulfate, filtered and concentrated *in vacuo*. Purification *via* silica gel chromatography (gradient elution 5 to 100% EtOAc in petroleum ether) yielded 2,4-dichloro-5-(cyclopropylmethyl)-5*H*-pyrrolo[3,2-d]pyrimidine **69** (1.05 g, 4.33 mmol, 82%) as a white solid. MS (ESI+) *m/z* calcd for $C_{10}H_{10}Cl_2N_3$ $[M + H]^+$ 242.0; found 242.1. UPLC (method A) $t_R$ = 2.95 min, 88%.

*2-Chloro-5-(cyclopropylmethyl)-N-(pyridin-2-ylmethyl)-5H-pyrrolo[3,2-d]pyrimidin-4-amine (**72**).*

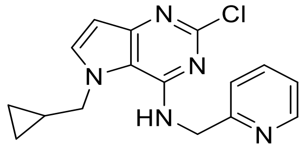

2,4-Dichloro-5-(cyclopropylmethyl)-5*H*-pyrrolo[3,2-d]pyrimidine **69** (0.55 g, 2.27 mmol) and pyridin-2-ylmethanamine (0.26 g, 2.39 mmol) were reacted according to general procedure 1 in chloroform (10 mL) at 90 °C for 48 h. Purification *via* silica gel chromatography (gradient elution 5 to 100% EtOAc in petroleum ether) gave 2-Chloro-5-(cyclopropylmethyl)-N-(pyridin-2-ylmethyl)-5H-pyrrolo[3,2-d]pyrimidin-4-amine **72** (0.58 g, 1.84 mmol, 81%) as a cream coloured solid. MS (ESI+) *m/z* calcd for $C_{16}H_{17}ClN_5$ $[M + H]^+$ 314.1; found 314.2. UPLC (method A) $t_R$ = 2.45 min, 80%.

*5-(Cyclopropylmethyl)-2-(2-isopropylphenyl)-N-(pyridin-2-ylmethyl)-5H-pyrrolo[3,2-d]pyrimidin-4-amine (**41**).*

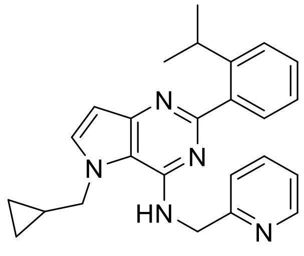

2-Chloro-5-(cyclopropylmethyl)-*N*-(pyridin-2-ylmethyl)-5*H*-pyrrolo[3,2-d]pyrimidin-4-amine **72** (80 mg, 0.26 mmol) and (2-isopropylphenyl)boronic acid (63 mg, 0.38 mmol) were reacted according to general procedure 2 for 40 mins at 125 °C. Upon cooling to room temperature the reaction mixture was loaded onto an SCX-II column, washed with MeOH, then eluted with 0.5 M $NH_3$ in MeOH and concentrated *in vacuo*. Purification *via* preparatory HPLC (gradient elution 5 to 95% MeCN in $H_2O$ with 0.1% $NH_3$) yielded 5-(cyclopropylmethyl)-2-(2-isopropylphenyl)-*N*-(pyridin-2-ylmethyl)-5*H*-pyrrolo[3,2-d]pyrimidin-4-amine **41** (80 mg, 0.20 mmol, 79%) as a white solid. MS (ESI+) *m/z* calcd for $C_{25}H_{28}N_5$ $[M + H]^+$ 398.2; found 398.4. UPLC (method C) $t_R$ = 5.58 min, >98%. $^1$H NMR (300 MHz, Chloroform-d) δ 8.60 (ddd, J = 4.9, 1.8, 1.0 Hz, 1H), 7.70 (td, J = 7.7, 1.8 Hz, 1H), 7.63 (ddd, J = 7.5, 1.5, 0.5 Hz, 1H), 7.43 (dd, J = 7.8, 1.6 Hz, 1H), 7.38 (dd, J = 7.1, 1.5 Hz, 1H), 7.33 (dt, J = 7.9, 1.1 Hz, 1H), 7.30 – 7.27 (m, 1H), 7.26-7.25 (m, 1H), 7.25 – 7.21 (m, 1H), 6.92 (t, J = 4.3 Hz, 1H), 6.59 (d, J = 3.1 Hz, 1H), 4.97 (d, J = 4.2 Hz, 2H), 4.35 (d, J = 6.6 Hz, 2H), 3.60 (hept, J = 6.9 Hz, 1H), 1.48 (ttt, J = 8.0, 6.6, 4.9 Hz, 1H), 1.26 (d, J = 6.9 Hz, 6H), 0.80 – 0.63 (m, 2H), 0.58 – 0.43 (m, 2H).

*2,4-Dichloro-5-((2-(trimethylsilyl)ethoxy)methyl)-5H-pyrrolo[3,2-d]pyrimidine (**70**).*

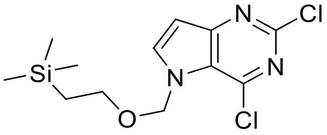


To a stirred solution of 2,4-dichloro-5*H*-pyrrolo[3,2-d]pyrimidine **67** (1.00 g, 5.32mmol) in DMF (12mL) at room temperature was added sodium hydride (60% dispersion in mineral oil) (0.26 g, 6.38mmol) in one portion and the reaction allowed to stir for 15 minutes before 2-(chloromethoxyethyl)trimethyl silane (1.03 mL, 0.98 g, 5.85mmol) was added *via* syringe and the reaction stirred at room temperature overnight. The reaction was diluted with water and EtOAc, extracted into EtOAc (x3), dried over sodium sulfate, filtered and concentrated *in vacuo*. Purification *via* silica gel chromatography (gradient elution 5 to 100% EtOAc in petroleum ether) yielded 2,4-dichloro-5-((2-(trimethylsilyl)ethoxy)methyl)-5*H*-pyrrolo[3,2-d]pyrimidine **70** (0.80 g, 2.51 mmol, 47%) as a white solid. MS (ESI+) *m/z* calcd for $C_{12}H_{18}Cl_2N_3OSi$ $[M + H]^+$ 318.1; found 318.0. UPLC (method B) $t_R$ = 3.57 min, 86%.

*2-Chloro-N-(pyridin-2-ylmethyl)-5-((2-(trimethylsilyl)ethoxy)methyl)-5H-pyrrolo[3,2-d]pyrimidin-4-amine (**73**).*

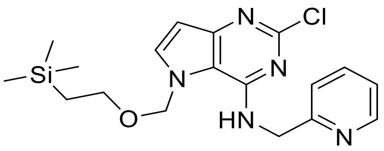

2,4-Dichloro-5-((2-(trimethylsilyl)ethoxy)methyl)-5*H*-pyrrolo[3,2-d]pyrimidine **70** (798 mg, 2.51 mmol) and pyridin-2-ylmethanamine (298 mg, 2.76 mmol) were reacted according to general procedure 1 in chloroform (10 mL) overnight at room temperature followed by 6 h at 50 °C to give 2-chloro-*N*-(pyridin-2-ylmethyl)-5-((2-(trimethylsilyl)ethoxy)methyl)-5*H*-pyrrolo[3,2-d]pyrimidin-4-amine **73** (910 mg, 2.33 mmol, 93%) as a white solid which was used without further purification. MS (ESI+) *m/z* calcd for $C_{18}H_{25}ClN_5OSi$ $[M + H]^+$ 390.2; found 390.1. UPLC (method B) $t_R$ = 3.07 min, 78%.

*2-(2-Isopropylphenyl)-N-(pyridin-2-ylmethyl)-5-((2-(trimethylsilyl)ethoxy)methyl)-5H-pyrrolo[3,2-d]pyrimidin-4-amine (**74**).*

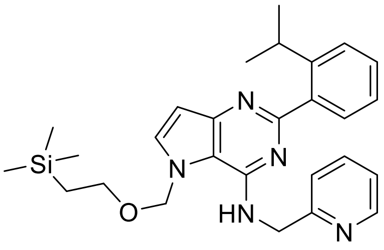

2-Chloro-*N*-(pyridin-2-ylmethyl)-5-((2-(trimethylsilyl)ethoxy)methyl)-5*H*-pyrrolo[3,2-d]pyrimidin-4-amine **73** (500 mg, 1.28 mmol) and (2-propan-2-ylphenyl)boronic acid (315 mg, 1.92 mmol) were reacted according to general procedure 2 for 60 min at 125 °C. Purification *via* silica gel chromatography (gradient elution 5 to 100% EtOAc in petroleum ether) yielded 2-(2-isopropylphenyl)-*N*-(pyridin-2-ylmethyl)-5-((2-(trimethylsilyl)ethoxy)methyl)-5*H*-pyrrolo[3,2-d]pyrimidin-4-amine **74** (563 mg, 1.19 mmol, 93%) as a white solid. MS (ESI+) *m/z* calcd for $C_{27}H_{36}N_5OSi$ $[M + H]^+$ 474.3; found 474.2. UPLC (method B) $t_R$ = 2.97 min, 84%.

*2-(2-Isopropylphenyl)-N-(pyridin-2-ylmethyl)-5H-pyrrolo[3,2-d]pyrimidin-4-amine (**42**).*

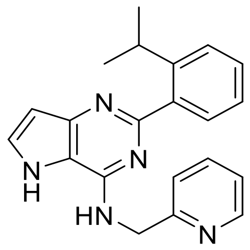

To a stirred solution of 2-(2-isopropylphenyl)-*N*-(pyridin-2-ylmethyl)-5-((2-(trimethylsilyl)ethoxy)methyl)-5*H*-pyrrolo[3,2-d]pyrimidin-4-amine **74** (150 mg, 0.320mmol) in DCM (5mL) was added trifluoroacetic acid (1 mL) and the reaction left to stir overnight at room temperature. The DCM was allowed to evaporate and the reaction allowed to stand for a further 18h. The reaction was concentrated under reduced pressure, dissolved in 7M methanolic ammonia, stirred for 3 h and concentrated under reduced pressure. Purification *via* preparatory HPLC (gradient elution 5 to 95% MeCN in $H_2O$ with 0.1% $NH_3$) yielded 2-(2-isopropylphenyl)-N-(pyridin-2-ylmethyl)-5H-pyrrolo[3,2-d]pyrimidin-4-amine **42** (26 mg, 0.076 mmol, 24%) as a white solid. MS (ESI+) *m/z* calcd for $C_{21}H_{22}N_5$ $[M + H]^+$ 344.2; found 344.1. UPLC (method C) $t_R$ = 4.53 min, >98%. $^1$H NMR (300 MHz, Chloroform-d) δ 10.28 (s, 1H), 8.55 (ddd, J = 5.0, 1.8, 0.9 Hz, 1H), 7.71 (td, J = 7.7, 1.8 Hz, 1H), 7.59 – 7.53 (m, 1H), 7.41 – 7.35 (m, 2H), 7.35 – 7.30 (m, 1H), 7.28 – 7.17 (m, 3H), 6.66 (t, J = 5.3 Hz, 1H), 6.55 (d, J = 3.1 Hz, 1H), 4.91 (d, J = 5.4 Hz, 2H), 3.58 – 3.42 (m, 1H), 1.18 (d, J = 6.9 Hz, 6H).

## NMR Spectra of compounds from Table 2:

Compound **1**:

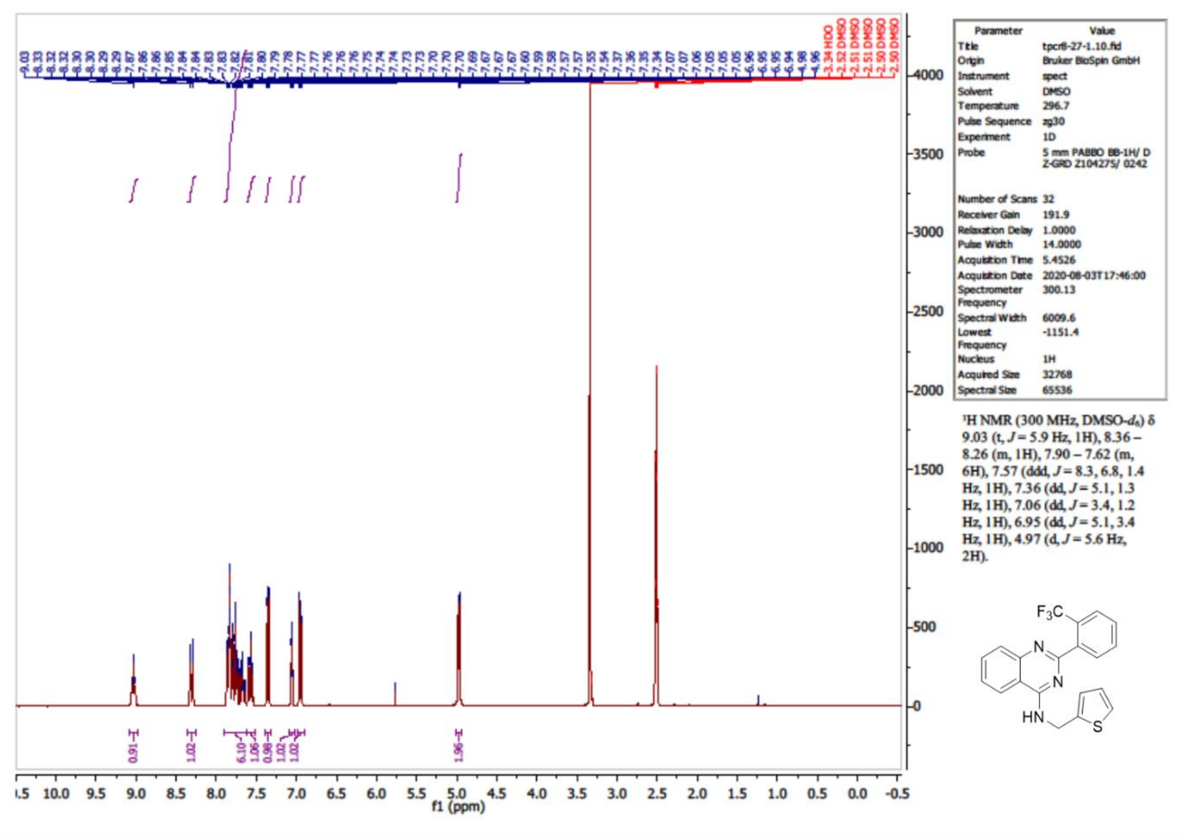


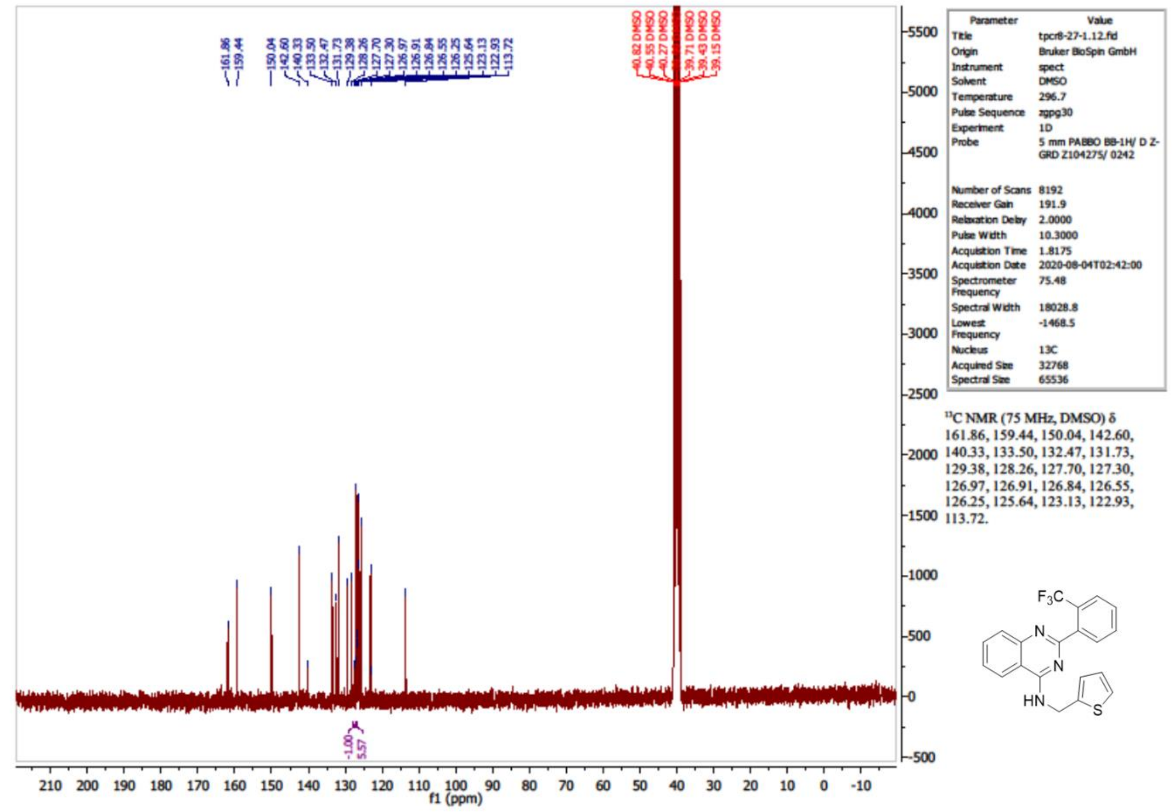

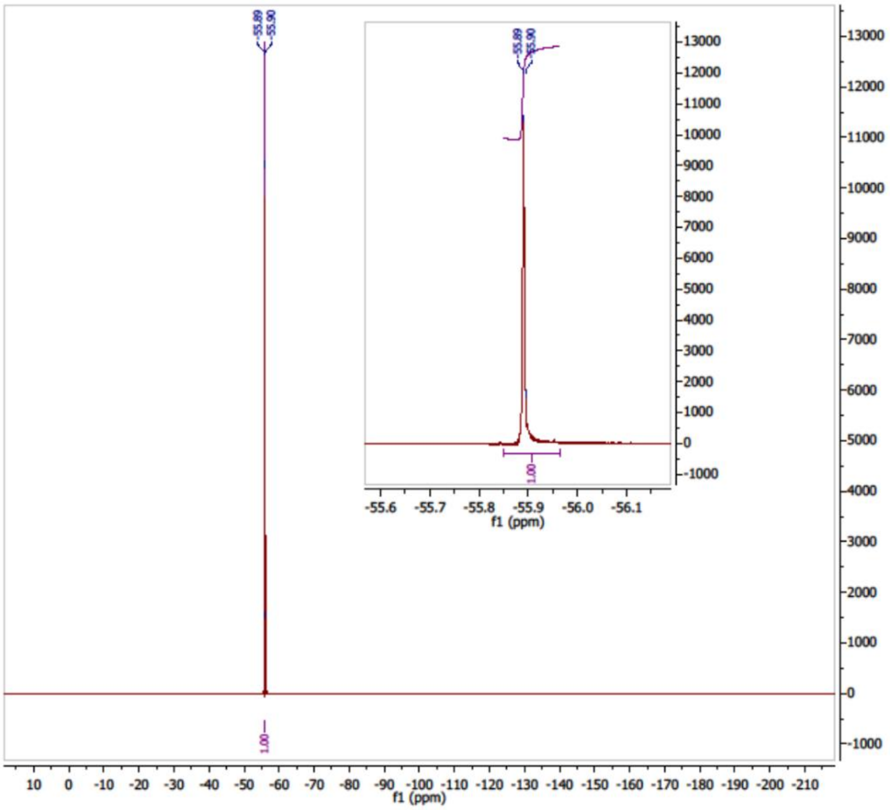


| Parameter | Value |
|---|---|
| Title | tpcr8-27-1.11.fid |
| Origin | Bruker BioSpin GmbH |
| Instrument | spect |
| Solvent | DMSO |
| Temperature | 296.9 |
| Pulse Sequence | zgfhigqn.2 |
| Experiment | 1D |
| Probe | 5 mm PABBO BB-1H/ D Z-GRD Z104275/ 0242 |
| Number of Scans | 32 |
| Receiver Gain | 191.9 |
| Relaxation Delay | 1.0000 |
| Pulse Width | 15.0000 |
| Acquisition Time | 0.9787 |
| Acquisition Date | 2020-08-03T17:50:00 |
| Spectrometer Frequency | 282.38 |
| Spectral Width | 66964.3 |
| Lowest Frequency | -61722.4 |
| Nucleus | 19F |
| Acquired Size | 65536 |
| Spectral Size | 131072 |

$^{19}F$ NMR (282 MHz, DMSO-$d_6$) δ -55.89.

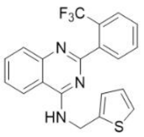


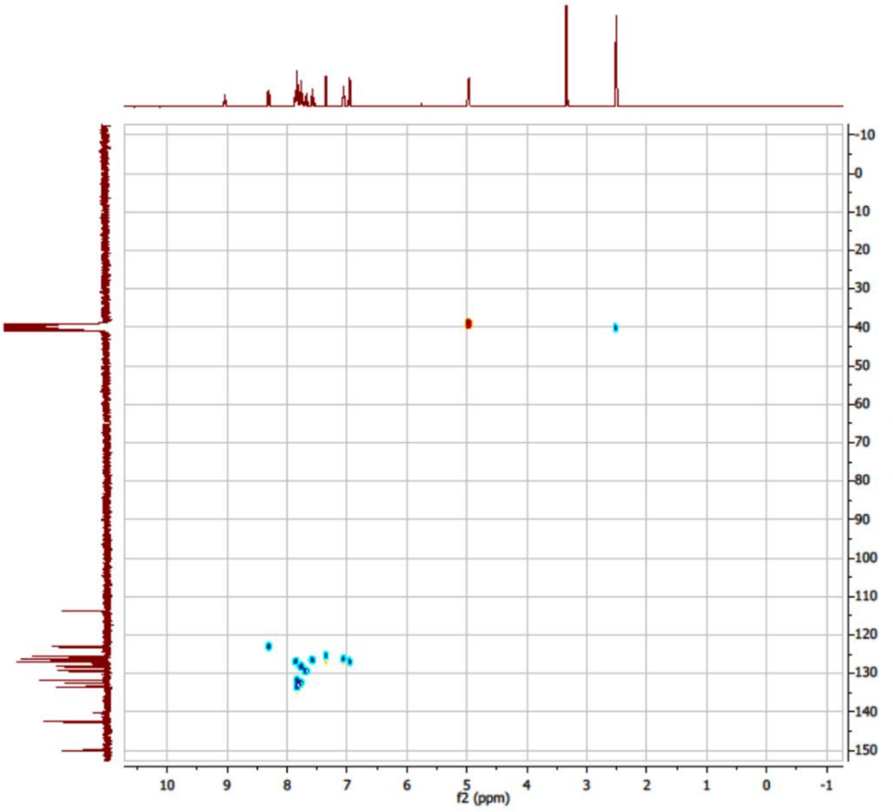


| Parameter | Value |
|---|---|
| Title | tpcr8-27-1_2D.12.ser |
| Origin | Bruker BioSpin GmbH |
| Instrument | spect |
| Solvent | DMSO |
| Temperature | 296.6 |
| Pulse Sequence | hsqcedetgpsp.3 |
| Experiment | HSQC-EDITED |
| Probe | 5 mm PABBO BB-1H/ D Z-GRD Z104275/ 0242 |
| Number of Scans | 32 |
| Receiver Gain | 191.9 |
| Relaxation Delay | 1.0000 |
| Pulse Width | 14.0000 |
| Acquisition Time | 0.1420 |
| Acquisition Date | 2021-09-13T21:17:00 |
| Spectrometer Frequency | (300.13, 75.47) |
| Spectral Width | (3605.8, 12500.0) |
| Lowest Frequency | (-388.5, -968.1) |
| Nucleus | (1H, 13C) |
| Acquired Size | (512, 256) |
| Spectral Size | (512, 512) |

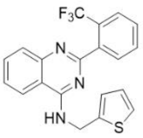

Compound **14**:

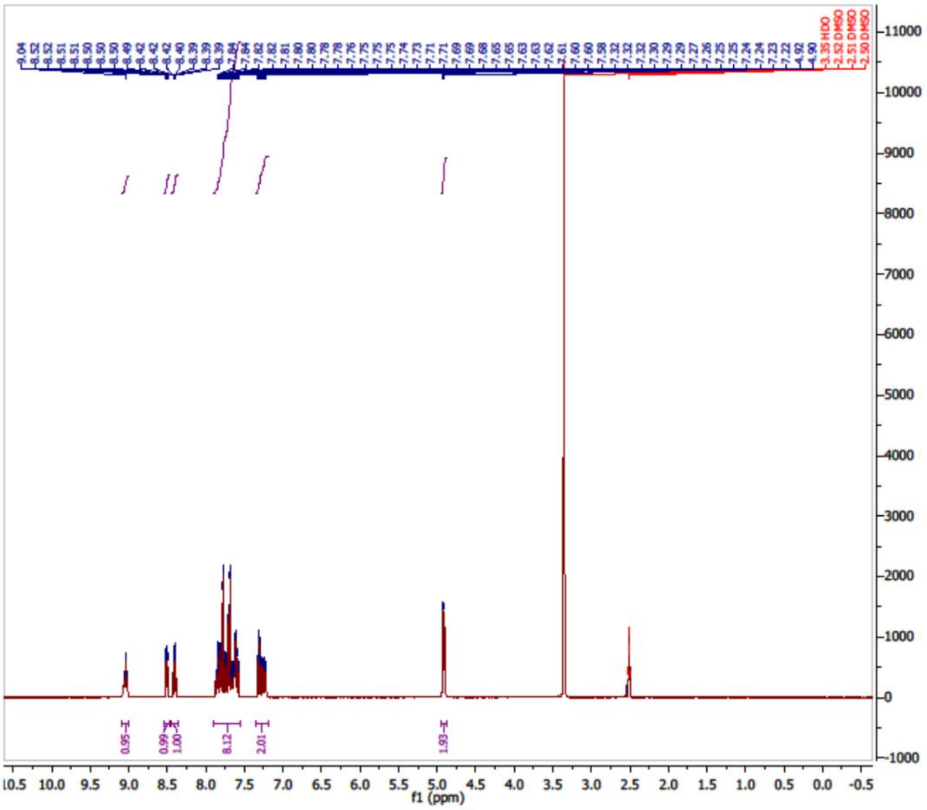


| Parameter | Value |
|---|---|
| Title | tpcr8-27-2-3.10.fid |
| Origin | Bruker BioSpin GmbH |
| Instrument | spect |
| Solvent | DMSO |
| Temperature | 299.1 |
| Pulse Sequence | zg30 |
| Experiment | 1D |
| Probe | 5 mm PABBO BB-1H/ D Z-GRD Z104275/ 0242 |
| Number of Scans | 16 |
| Receiver Gain | 191.9 |
| Relaxation Delay | 1.0000 |
| Pulse Width | 14.0000 |
| Acquisition Time | 5.4526 |
| Acquisition Date | 2020-09-03T14:27:00 |
| Spectrometer Frequency | 300.13 |
| Spectral Width | 6009.6 |
| Lowest Frequency | -1151.4 |
| Nucleus | 1H |
| Acquired Size | 32768 |
| Spectral Size | 65536 |

$^1$H NMR (300 MHz, DMSO-$d_6$) δ 9.04 (t, $J$ = 5.9 Hz, 1H), 8.51 (ddd, $J$ = 4.9, 1.8, 1.0 Hz, 1H), 8.45 – 8.36 (m, 1H), 7.90 – 7.54 (m, 8H), 7.35 – 7.19 (m, 2H), 4.91 (d, $J$ = 5.8 Hz, 2H).

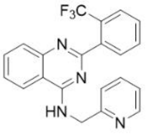

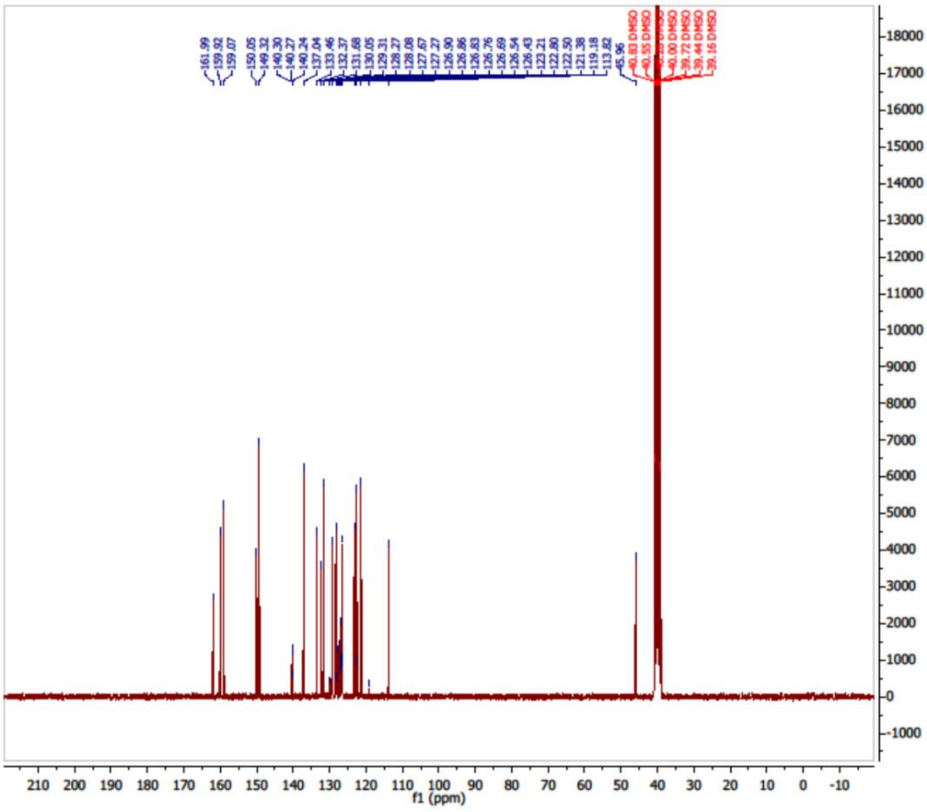


| Parameter | Value |
|---|---|
| Title | tpcr8-27-2-3.12.fid |
| Origin | Bruker BioSpin GmbH |
| Instrument | spect |
| Solvent | DMSO |
| Temperature | 297.7 |
| Pulse Sequence | zgpg30 |
| Experiment | 1D |
| Probe | 5 mm PABBO BB-1H/ D Z-GRD Z104275/ 0242 |
| Number of Scans | 8192 |
| Receiver Gain | 191.9 |
| Relaxation Delay | 2.0000 |
| Pulse Width | 10.3000 |
| Acquisition Time | 1.8175 |
| Acquisition Date | 2020-09-03T23:24:00 |
| Spectrometer Frequency | 75.48 |
| Spectral Width | 18028.8 |
| Lowest Frequency | -1468.5 |
| Nucleus | 13C |
| Acquired Size | 32768 |
| Spectral Size | 65536 |

$^{13}$C NMR (75 MHz, DMSO) δ 161.99, 159.92, 159.07, 150.05, 149.32, 140.30, 140.27, 140.24, 137.04, 133.46, 132.37, 131.68, 130.05, 129.31, 128.27, 128.08, 127.67, 127.27, 126.90, 126.86, 126.83, 126.76, 126.69, 126.54, 126.43, 123.21, 122.80, 122.50, 121.38, 119.18, 113.82, 45.96.

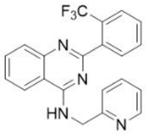

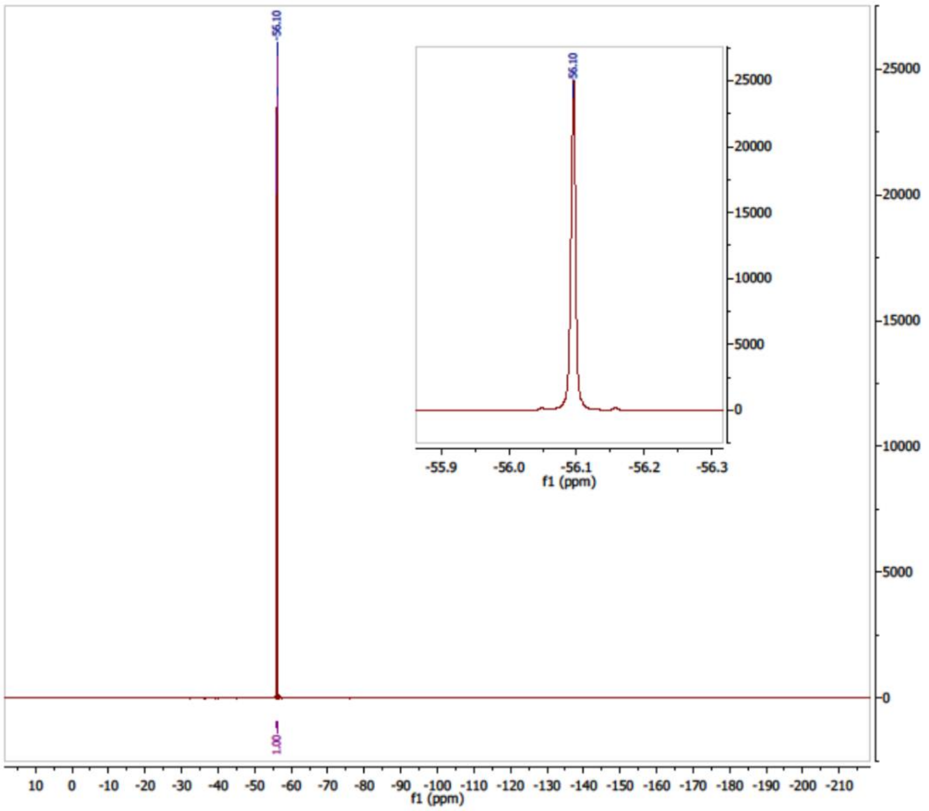


| Parameter | Value |
|---|---|
| Title | tpcr8-27-2-3.11.fid |
| Origin | Bruker BioSpin GmbH |
| Instrument | spect |
| Solvent | DMSO |
| Temperature | 299.1 |
| Pulse Sequence | zgfiqn |
| Experiment | 1D |
| Probe | 5 mm PABBO BB-1H/ D Z-GRD Z104275/ 0242 |
| Number of Scans | 32 |
| Receiver Gain | 191.9 |
| Relaxation Delay | 1.0000 |
| Pulse Width | 15.0000 |
| Acquisition Time | 0.9787 |
| Acquisition Date | 2020-09-03T14:31:00 |
| Spectrometer Frequency | 282.38 |
| Spectral Width | 66964.3 |
| Lowest Frequency | -61722.4 |
| Nucleus | 19F |
| Acquired Size | 65536 |
| Spectral Size | 131072 |

$^{19}$F NMR (282 MHz, DMSO-$d_6$) δ -56.10.

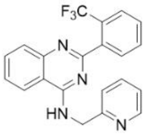

Compound **22**:

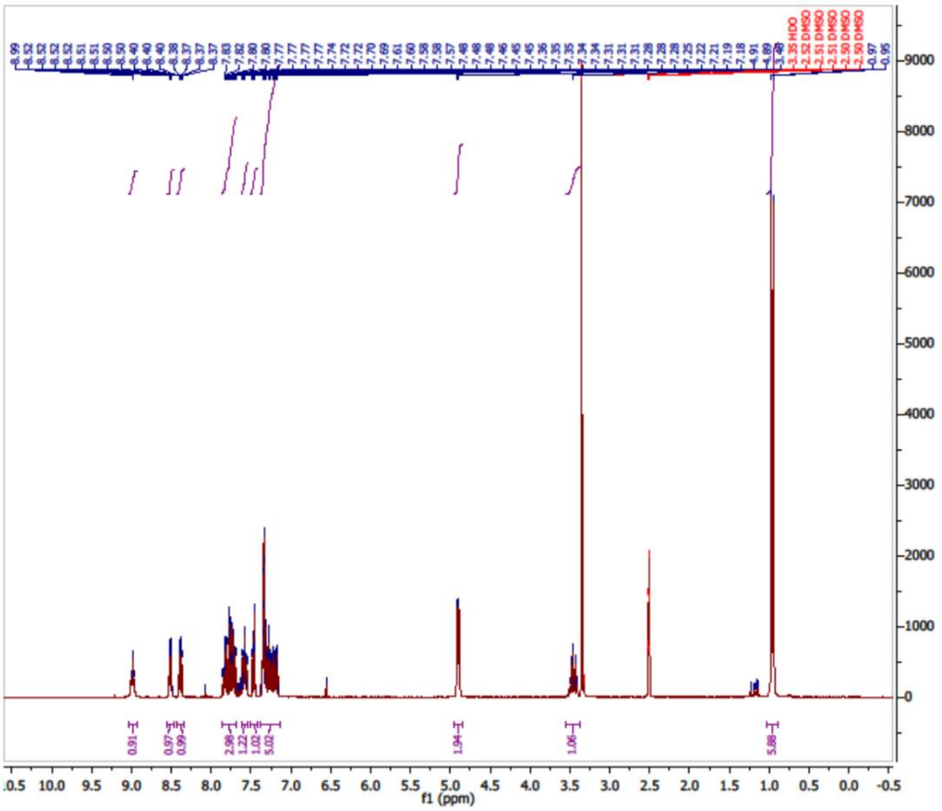


| Parameter | Value |
|---|---|
| Title | tpcr8-27-3.30.fid |
| Origin | Bruker BioSpin GmbH |
| Instrument | spect |
| Solvent | DMSO |
| Temperature | 295.8 |
| Pulse Sequence | zg30 |
| Experiment | 1D |
| Probe | 5 mm PABBO BB-1H/ D Z-GRD Z104275/ 0242 |
| Number of Scans | 32 |
| Receiver Gain | 191.9 |
| Relaxation Delay | 1.0000 |
| Pulse Width | 14.0000 |
| Acquisition Time | 5.4526 |
| Acquisition Date | 2020-08-04T07:25:00 |
| Spectrometer Frequency | 300.13 |
| Spectral Width | 6009.6 |
| Lowest Frequency | -1151.4 |
| Nucleus | 1H |
| Acquired Size | 32768 |
| Spectral Size | 65536 |

$^1$H NMR (300 MHz, DMSO-$d_6$) δ 8.99 (t, $J$ = 6.0 Hz, 1H), 8.51 (ddd, $J$ = 4.9, 1.9, 1.0 Hz, 1H), 8.43 – 8.34 (m, 1H), 7.86 – 7.68 (m, 3H), 7.61 – 7.54 (m, 1H), 7.47 (dt, $J$ = 7.6, 1.1 Hz, 1H), 7.39 – 7.14 (m, 5H), 4.90 (d, $J$ = 5.8 Hz, 2H), 3.46 (p, $J$ = 6.9 Hz, 1H), 0.96 (d, $J$ = 6.9 Hz, 6H).

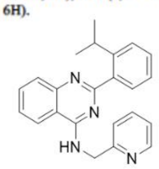

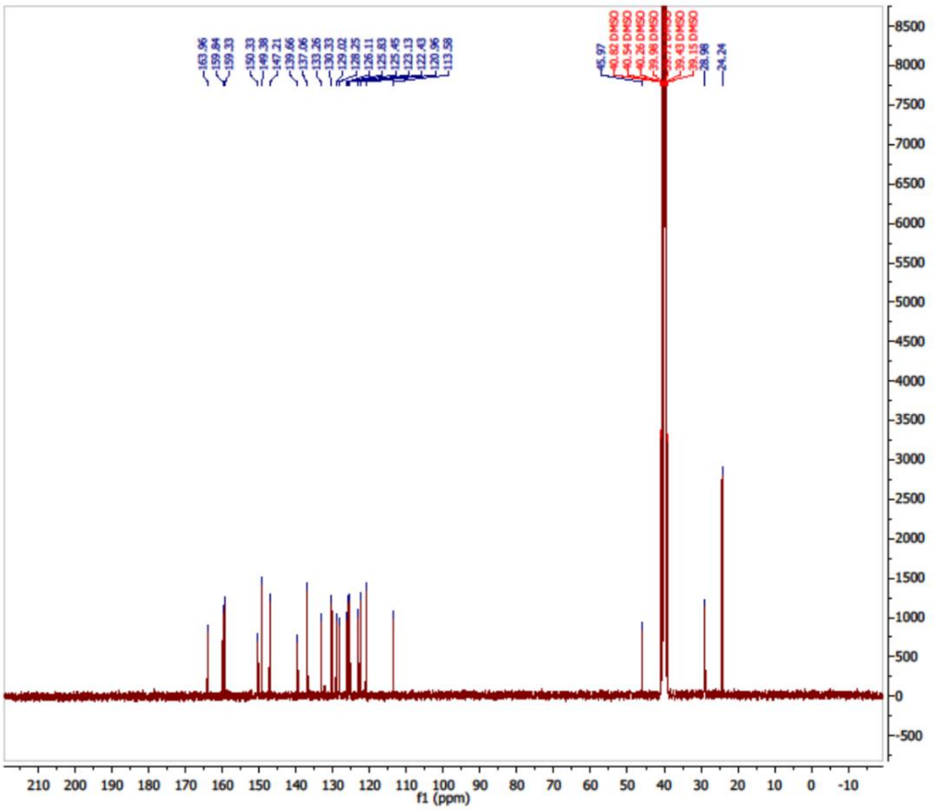


| Parameter | Value |
|---|---|
| Title | tpcr8-27-3.33.fid |
| Origin | Bruker BioSpin GmbH |
| Instrument | spect |
| Solvent | DMSO |
| Temperature | 297.1 |
| Pulse Sequence | zgpg30 |
| Experiment | 1D |
| Probe | 5 mm PABBO BB-1H/ D Z-GRD Z104275/ 0242 |
| Number of Scans | 4096 |
| Receiver Gain | 191.9 |
| Relaxation Delay | 2.0000 |
| Pulse Width | 10.3000 |
| Acquisition Time | 1.8175 |
| Acquisition Date | 2020-08-04T11:56:00 |
| Spectrometer Frequency | 75.48 |
| Spectral Width | 18028.8 |
| Lowest Frequency | -1468.5 |
| Nucleus | 13C |
| Acquired Size | 32768 |
| Spectral Size | 65536 |

$^{13}$C NMR (75 MHz, DMSO) δ 163.96, 159.84, 159.33, 150.33, 149.38, 147.21, 139.66, 137.06, 133.26, 130.33, 129.02, 128.25, 126.11, 125.83, 125.45, 123.13, 122.43, 120.96, 113.58, 45.97, 28.98, 24.24.

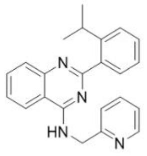

Compound **35**:

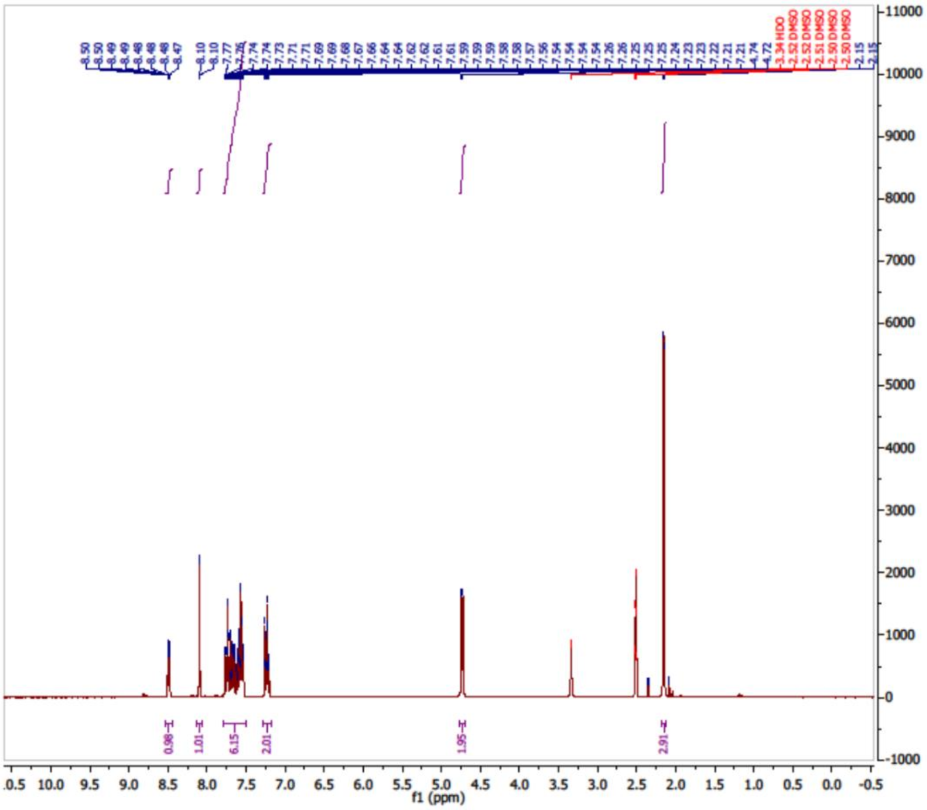

| Parameter | Value |
|---|---|
| Title | tpcr8-027-6.50.fid |
| Origin | Bruker BioSpin GmbH |
| Instrument | spect |
| Solvent | DMSO |
| Temperature | 297.7 |
| Pulse Sequence | zg30 |
| Experiment | 1D |
| Probe | 5 mm PABBO BB-1H/ D Z-GRD Z104275/ 0242 |
| Number of Scans | 32 |
| Receiver Gain | 191.9 |
| Relaxation Delay | 1.0000 |
| Pulse Width | 14.0000 |
| Acquisition Time | 5.4526 |
| Acquisition Date | 2020-08-05T22:05:00 |
| Spectrometer Frequency | 300.13 |
| Spectral Width | 6009.6 |
| Lowest Frequency | -1151.4 |
| Nucleus | 1H |
| Acquired Size | 32768 |
| Spectral Size | 65536 |

$^1$H NMR (300 MHz, DMSO-$d_6$) δ 8.49 (ddd, $J$ = 4.8, 1.9, 1.0 Hz, 1H), 8.10 (d, $J$ = 1.0 Hz, 1H), 7.80 – 7.51 (m, 6H), 7.29 – 7.18 (m, 2H), 4.73 (d, $J$ = 5.8 Hz, 2H), 2.15 (d, $J$ = 0.9 Hz, 3H).

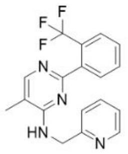

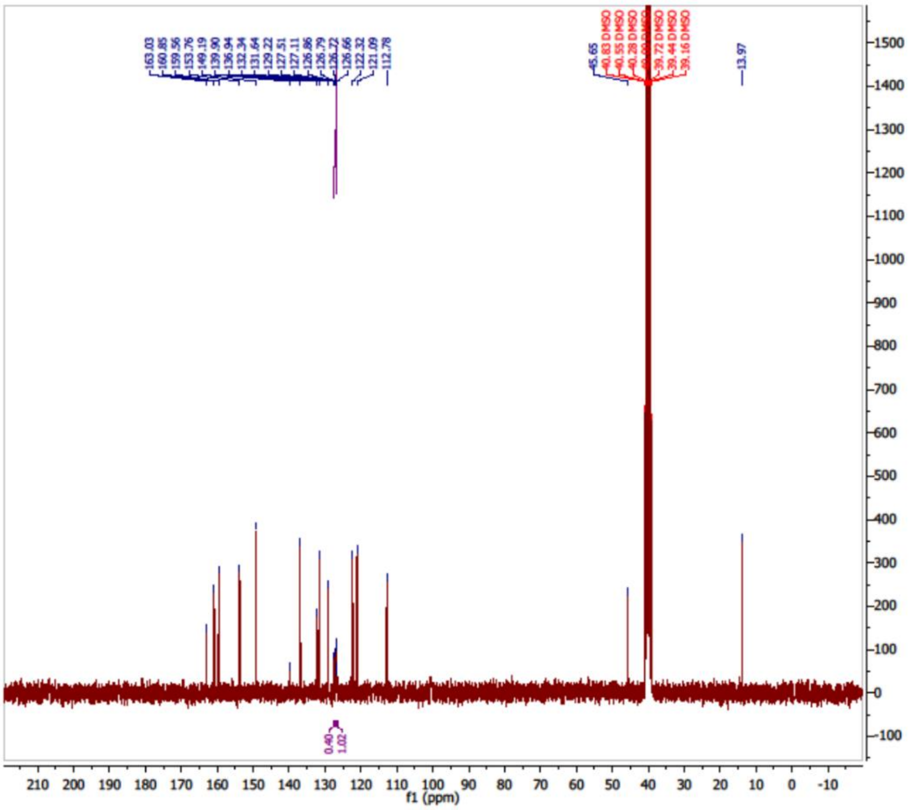

| Parameter | Value |
|---|---|
| Title | tpcr8-027-6.53.fid |
| Origin | Bruker BioSpin GmbH |
| Instrument | spect |
| Solvent | DMSO |
| Temperature | 298.3 |
| Pulse Sequence | zgpg30 |
| Experiment | 1D |
| Probe | 5 mm PABBO BB-1H/ D Z-GRD Z104275/ 0242 |
| Number of Scans | 4096 |
| Receiver Gain | 36.3 |
| Relaxation Delay | 2.0000 |
| Pulse Width | 10.3000 |
| Acquisition Time | 1.8175 |
| Acquisition Date | 2020-08-06T02:37:00 |
| Spectrometer Frequency | 75.48 |
| Spectral Width | 18028.8 |
| Lowest Frequency | -1468.5 |
| Nucleus | 13C |
| Acquired Size | 32768 |
| Spectral Size | 65536 |

$^{13}$C NMR (75 MHz, DMSO) δ 163.03, 160.85, 159.56, 153.76, 149.19, 139.90, 136.94, 132.34, 131.64, 129.22, 127.51, 127.11, 126.86, 126.79, 126.72, 126.66, 122.32, 121.09, 112.78, 45.65, 13.97.

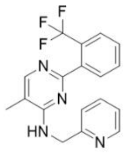

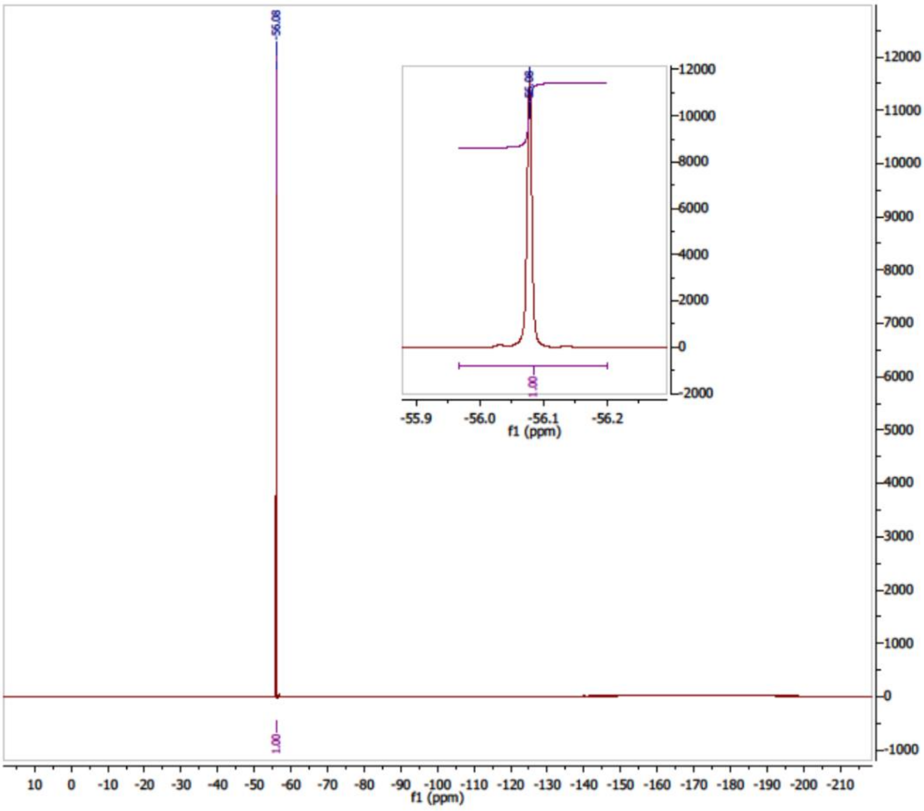

| Parameter | Value |
| --- | --- |
| Title | tpcr8-027-6.52.fid |
| Origin | Bruker BioSpin GmbH |
| Instrument | spect |
| Solvent | DMSO |
| Temperature | 297.7 |
| Pulse Sequence | zgfqn |
| Experiment | 1D |
| Probe | 5 mm PABBO BB-1H/ D Z-GRD Z104275/ 0242 |
| Number of Scans | 32 |
| Receiver Gain | 191.9 |
| Relaxation Delay | 1.0000 |
| Pulse Width | 15.0000 |
| Acquisition Time | 0.9787 |
| Acquisition Date | 2020-08-05T22:09:00 |
| Spectrometer Frequency | 282.38 |
| Spectral Width | 66964.3 |
| Lowest Frequency | -61722.4 |
| Nucleus | 19F |
| Acquired Size | 65536 |
| Spectral Size | 131072 |

$^{19}$F NMR (282 MHz, DMSO-$d_6$) δ -56.08.

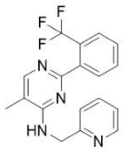

Compound **38**:

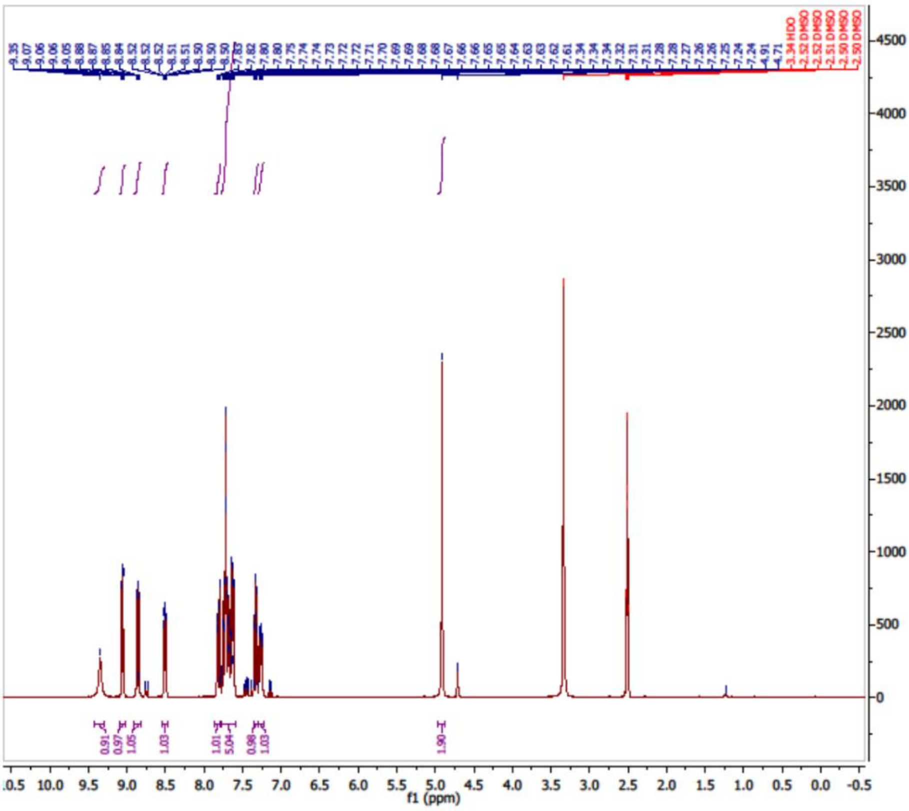

| Parameter | Value |
| --- | --- |
| Title | tpcr8-027-5.40.fid |
| Origin | Bruker BioSpin GmbH |
| Instrument | spect |
| Solvent | DMSO |
| Temperature | 299.0 |
| Pulse Sequence | zg30 |
| Experiment | 1D |
| Probe | 5 mm PABBO BB-1H/ D Z-GRD Z104275/ 0242 |
| Number of Scans | 32 |
| Receiver Gain | 191.9 |
| Relaxation Delay | 1.0000 |
| Pulse Width | 14.0000 |
| Acquisition Time | 5.4526 |
| Acquisition Date | 2020-08-05T17:28:00 |
| Spectrometer Frequency | 300.13 |
| Spectral Width | 6009.6 |
| Lowest Frequency | -1151.4 |
| Nucleus | 1H |
| Acquired Size | 32768 |
| Spectral Size | 65536 |

$^1$H NMR (300 MHz, DMSO-$d_6$) δ 9.35 (s, 1H), 9.06 (dd, $J$ = 4.4, 1.8 Hz, 1H), 8.86 (dd, $J$ = 8.2, 1.9 Hz, 1H), 8.51 (ddd, $J$ = 4.9, 1.8, 1.0 Hz, 1H), 7.81 (dd, $J$ = 7.6, 1.3 Hz, 6H), 7.78 – 7.60 (m, 5H), 7.33 (dt, $J$ = 7.9, 1.1 Hz, 2H), 7.26 (ddd, $J$ = 7.5, 4.8, 1.2 Hz, 1H), 4.91 (s, 2H).

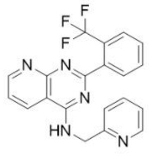

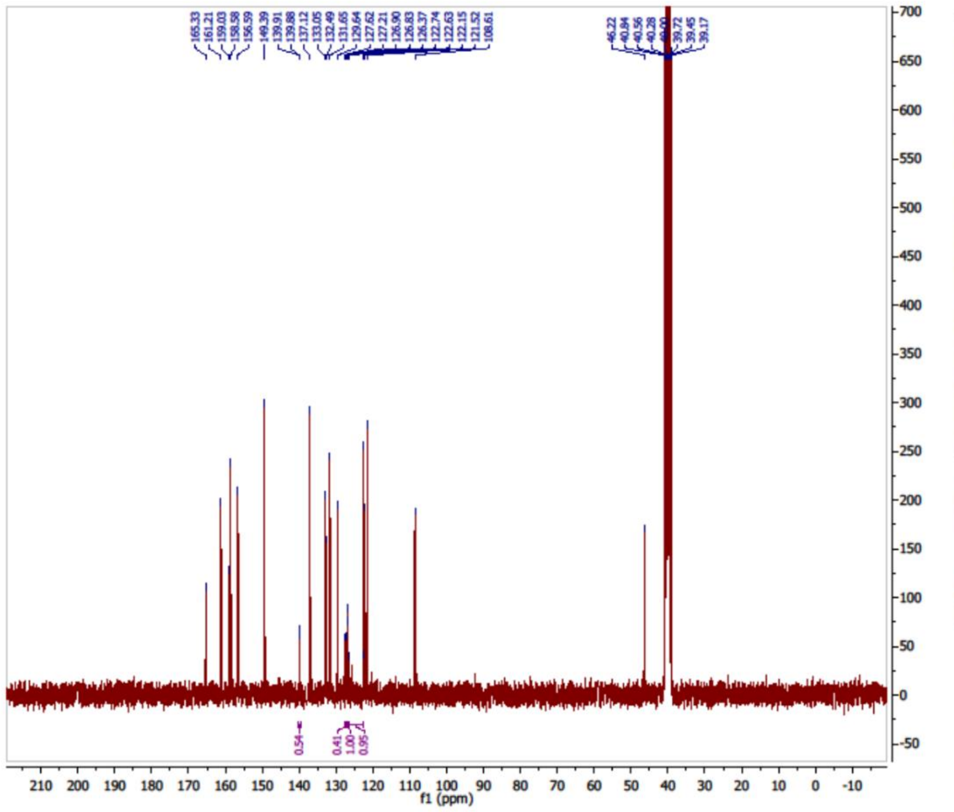


$^{13}$C NMR (75 MHz, DMSO) δ 165.33, 161.21, 159.03, 158.58, 156.59, 149.39, 139.91, 139.88, 137.12, 133.05, 132.49, 131.65, 129.64, 127.62, 127.21, 126.90, 126.83, 126.37, 122.74, 122.63, 122.15, 121.52, 108.61, 46.22, 40.84, 40.56, 40.28, 40.00, 39.72, 39.45, 39.17.

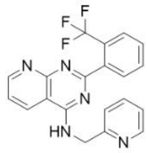

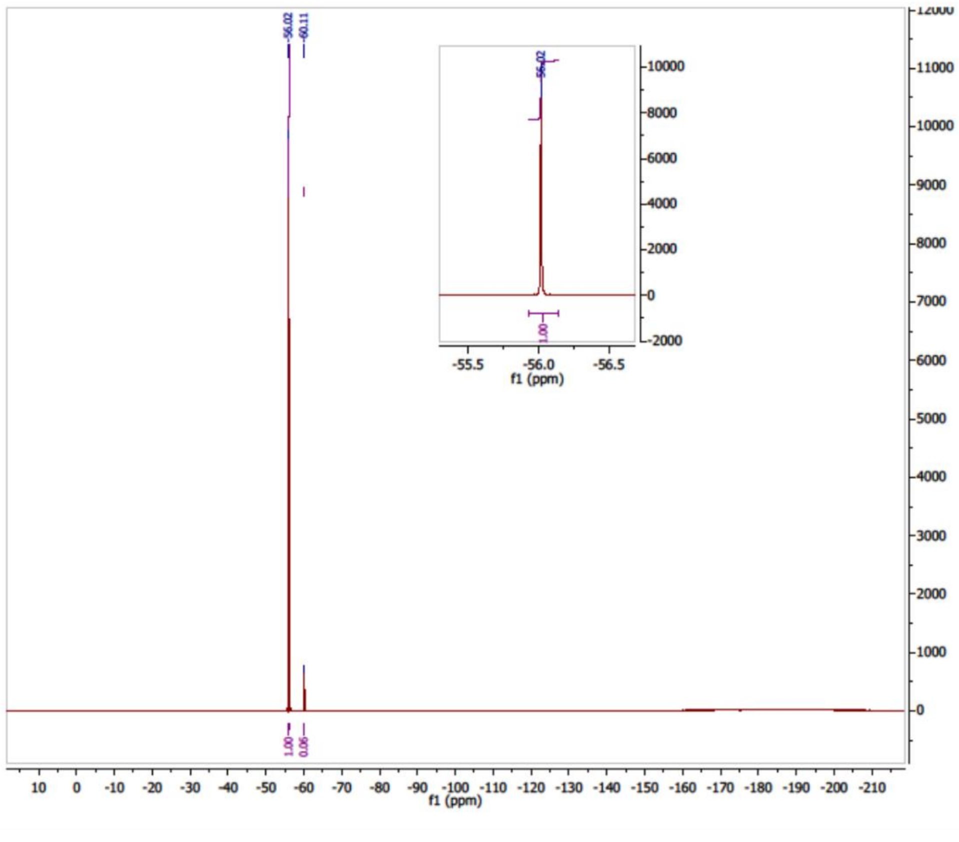


$^{19}$F NMR (282 MHz, DMSO-$d_6$) δ -56.02, -60.11.

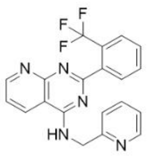

Compound **40**:

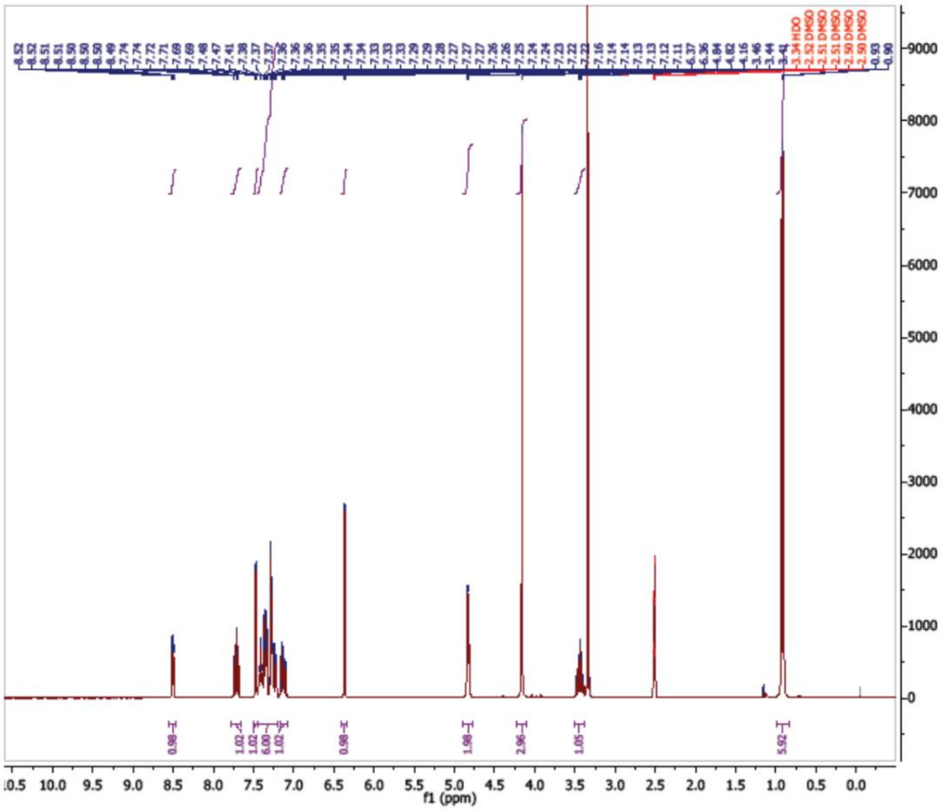


| Parameter | Value |
| --- | --- |
| Title | tpcr8-027-4.30.fid |
| Origin | Bruker BioSpin GmbH |
| Instrument | spect |
| Solvent | DMSO |
| Temperature | 297.9 |
| Pulse Sequence | zg30 |
| Experiment | 1D |
| Probe | 5 mm PABBO BB-1H/ D Z-GRD Z104275/ 0242 |
| Number of Scans | 32 |
| Receiver Gain | 191.9 |
| Relaxation Delay | 1.0000 |
| Pulse Width | 14.0000 |
| Acquisition Time | 5.4526 |
| Acquisition Date | 2020-08-05T12:54:00 |
| Spectrometer Frequency | 300.13 |
| Spectral Width | 6009.6 |
| Lowest Frequency | -1151.4 |
| Nucleus | 1H |
| Acquired Size | 32768 |
| Spectral Size | 65536 |

$^1H$ NMR (300 MHz, DMSO-$d_6$) δ 8.51 (ddd, $J$ = 4.9, 1.8, 0.9 Hz, 1H), 7.72 (td, $J$ = 7.7, 1.8 Hz, 1H), 7.47 (d, $J$ = 3.0 Hz, 1H), 7.45 – 7.19 (m, 6H), 7.13 (ddd, $J$ = 7.6, 5.2, 3.4 Hz, 1H), 6.37 (d, $J$ = 3.0 Hz, 1H), 4.83 (d, $J$ = 5.6 Hz, 2H), 4.16 (s, 3H), 3.44 (p, $J$ = 6.9 Hz, 1H), 0.92 (d, $J$ = 6.9 Hz, 6H).

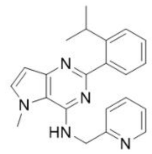


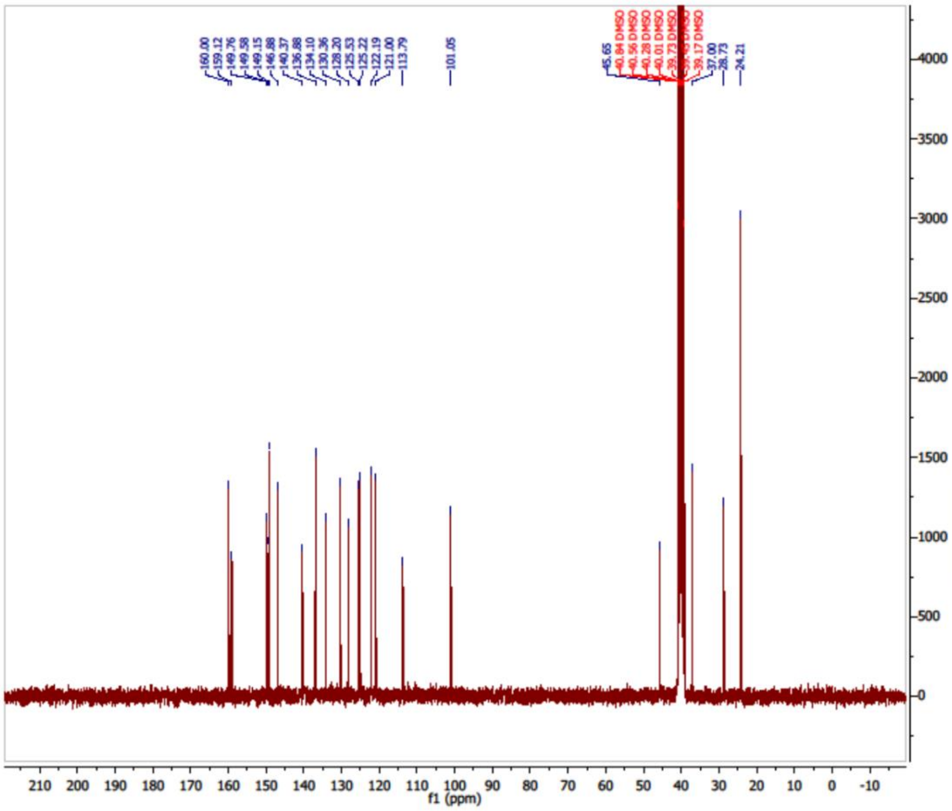


| Parameter | Value |
| --- | --- |
| Title | tpcr8-027-4.31.fid |
| Origin | Bruker BioSpin GmbH |
| Instrument | spect |
| Solvent | DMSO |
| Temperature | 299.6 |
| Pulse Sequence | zgpg30 |
| Experiment | 1D |
| Probe | 5 mm PABBO BB-1H/ D Z-GRD Z104275/ 0242 |
| Number of Scans | 4096 |
| Receiver Gain | 191.9 |
| Relaxation Delay | 2.0000 |
| Pulse Width | 10.3000 |
| Acquisition Time | 1.8175 |
| Acquisition Date | 2020-08-05T17:22:00 |
| Spectrometer Frequency | 75.48 |
| Spectral Width | 18028.8 |
| Lowest Frequency | -1468.5 |
| Nucleus | 13C |
| Acquired Size | 32768 |
| Spectral Size | 65536 |

$^{13}C$ NMR (75 MHz, DMSO) δ 160.00, 159.12, 149.76, 149.58, 149.15, 146.88, 140.37, 136.88, 134.10, 130.36, 128.20, 125.53, 125.22, 122.19, 121.00, 113.79, 101.05, 45.65, 37.00, 28.73, 24.21.

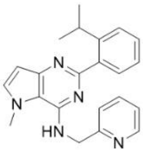

# HPLC traces of compounds from Table 2:

Compound **1**:

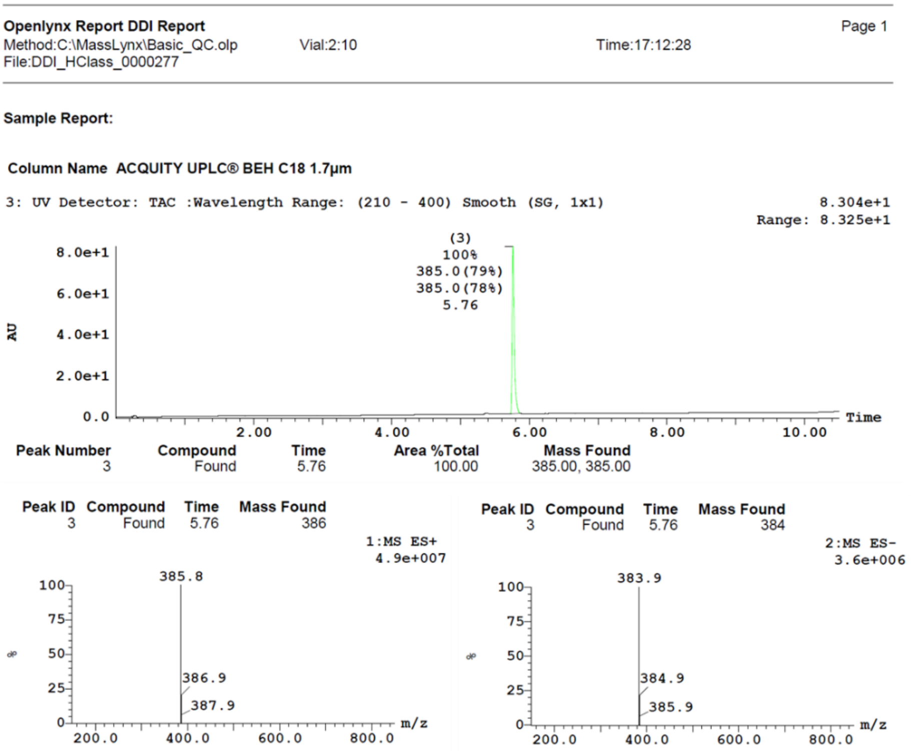


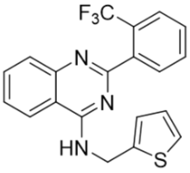

Exact Mass: 385.1

Compound **14**:

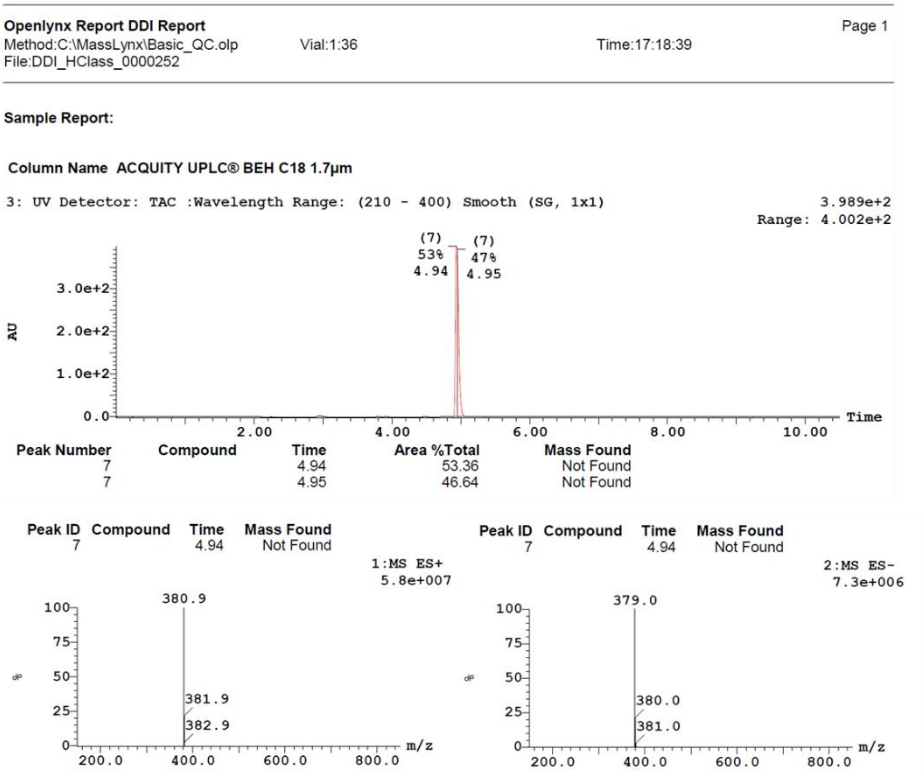


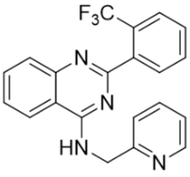

Exact Mass: 380.1

Compound **22**:

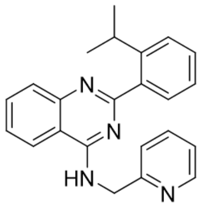

Exact Mass: 354.2

**Openlynx Report DDI Report** Page 1
Method:C:\MassLynx\b_basic_QC01.olp Vial:1:1 Time:17:47:14
File:DDI_HClass_0000789

**Sample Report:**

**Column Name ACQUITY UPLC® BEH C18 1.7µm**

3: UV Detector: TAC :Wavelength Range: (210 - 400) Smooth (SG, 1x1) 3.321e+2
Range: 3.333e+2

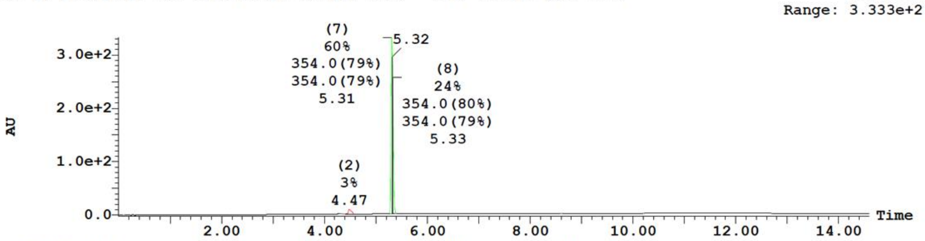


| Peak Number | Compound | Time | Area %Total | Mass Found |
|---|---|---|---|---|
| 2 | | 4.47 | 3.47 | Not Found |
| 7 | Found | 5.31 | 59.63 | 354.00, 354.00 |
| 7 | Found | 5.32 | 13.30 | 354.00, 354.00 |
| 8 | Found | 5.33 | 23.61 | 354.00, 354.00 |

| Peak ID | Compound | Time | Mass Found |
|---|---|---|---|
| 7 | Found | 5.32 | 355 |

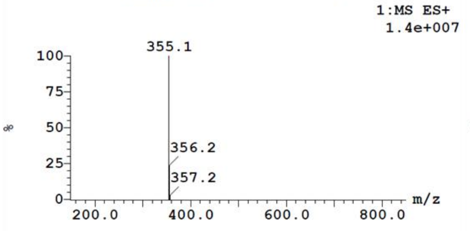


| Peak ID | Compound | Time | Mass Found |
|---|---|---|---|
| 8 | Found | 5.33 | 353 |

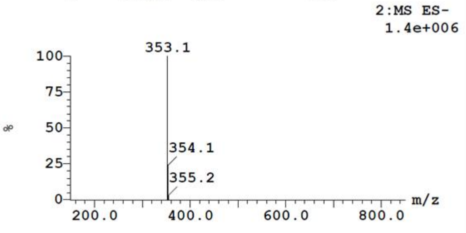


Compound **35**:

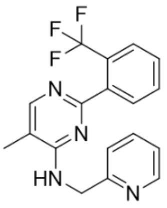

Exact Mass: 344.1

**Openlynx Report DDI Report** Page 1
Method:C:\MassLynx\b_basic_QC01.olp Vial:2:30 Time:09:31:26
File:DDI_HClass_0001661

**Sample Report:**

**Column Name ACQUITY UPLC® BEH C18 1.7µm**

3: UV Detector: TAC :Wavelength Range: (210 - 400) Smooth (SG, 1x1) 2.08e+2
Range: 2.115e+2

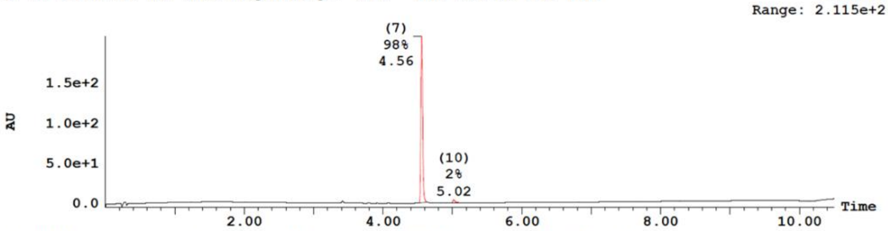


| Peak Number | Compound | Time | Area %Total | Mass Found |
|---|---|---|---|---|
| 7 | | 4.56 | 97.51 | Not Found |
| 10 | | 5.02 | 2.49 | Not Found |

| Peak ID | Compound | Time | Mass Found |
|---|---|---|---|
| 7 | | 4.57 | Not Found |

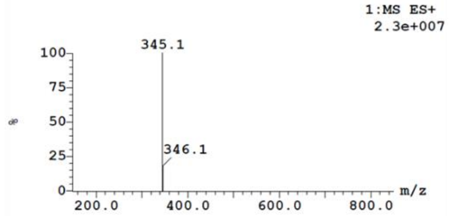


| Peak ID | Compound | Time | Mass Found |
|---|---|---|---|
| 7 | | 4.57 | Not Found |

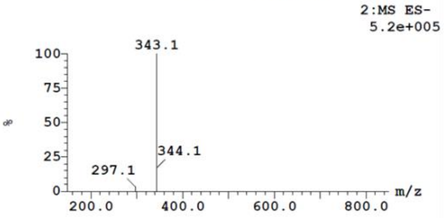

Compound **38**:

**Openlynx Report DDI Report** Page 1
Method:C:\MassLynx\b_acidic_QC03.olp Vial:1:9 Time:15:48:20
File:DDI_HClass_0024080

**Sample Report:**

**Column Name ACQUITY UPLC® HSS C18 1.8µm**

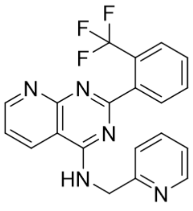

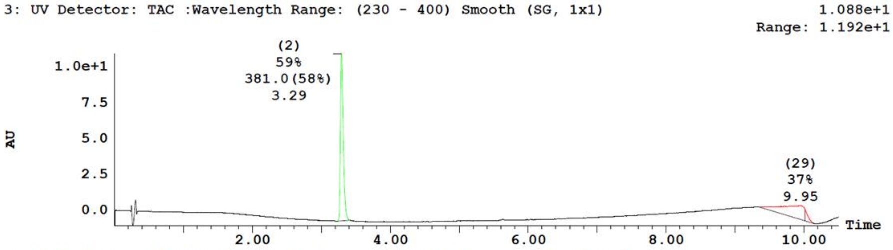


| Peak Number | Compound | Time | Area %Total | Mass Found |
|---|---|---|---|---|
| 2 | Found | 3.29 | 59.34 | 381.00 |
| 29 | | 9.95 | 36.58 | Not Found |
| 30 | | 10.02 | 4.08 | Not Found |

| Peak ID | Compound | Time | Mass Found |
|---|---|---|---|
| 3 | Found | 3.30 | 382 |

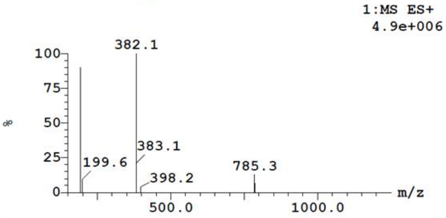


| Peak ID | Compound | Time | Mass Found |
|---|---|---|---|
| 2 | Found | 3.29 | 380 |

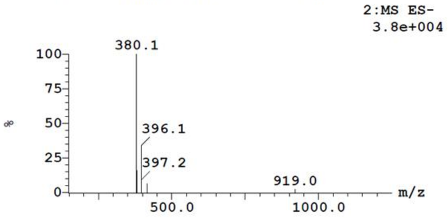


Compound **40**:

**Openlynx Report DDI Report** Page 1
Method:C:\MassLynx\b_basic_QC01.olp Vial:1:33 Time:09:08:46
File:DDI_HClass_0002138

**Sample Report:**

**Column Name ACQUITY UPLC® BEH C18 1.7µm**

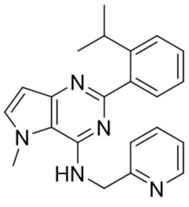

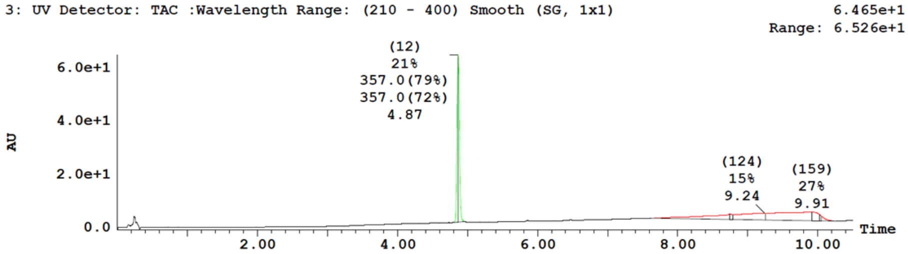


| Peak Number | Compound | Time | Area %Total | Mass Found |
|---|---|---|---|---|
| 12 | Found | 4.86 | 16.89 | 357.00, 357.00 |
| 12 | Found | 4.87 | 20.96 | 357.00, 357.00 |
| 99 | | 8.74 | 12.69 | Not Found |
| 100 | | 8.75 | 1.30 | Not Found |
| 124 | | 9.24 | 14.52 | Not Found |
| 159 | | 9.91 | 26.87 | Not Found |

| Peak ID | Compound | Time | Mass Found |
|---|---|---|---|
| 12 | Found | 4.86 | 358 |

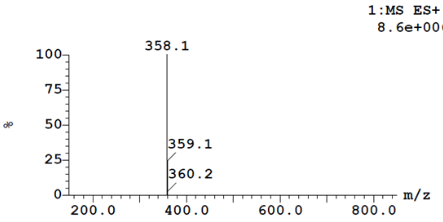


| Peak ID | Compound | Time | Mass Found |
|---|---|---|---|
| 12 | Found | 4.86 | 356 |

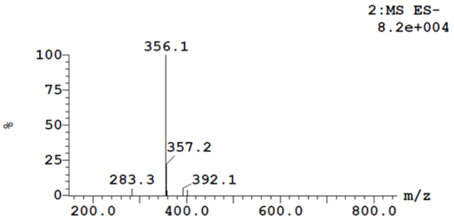

**References**


1) Clarke, J. H.; Giudici, M.-L.; Burke, J. E.; Williams, R. L.; Maloney, D. J.; Marugan, J.; Irvine, R. F. The Function of Phosphatidylinositol 5-Phosphate 4-Kinase γ (PI5P4Kγ) Explored Using a Specific Inhibitor That Targets the PI5P-Binding Site. Biochem. J. 2015, 466 (2), 359–367. https://doi.org/10.1042/BJ20141333.

2) Al-Ramahi, I.; Giridharan, S. S. P.; Chen, Y. C.; Patnaik, S.; Safren, N.; Hasegawa, J.; de Haro, M.; Gee, A. K. W.; Titus, S. A.; Jeong, H.; Clarke, J.; Krainc, D.; Zheng, W.; Irvine, R. F.; Barmada, S.; Ferrer, M.; Southall, N.; Weisman, L. S.; Botas, J.; Marugan, J. J. Inhibition of PIP4Kγ Ameliorates the Pathological Effects of Mutant Huntingtin Protein. Elife 2017, 6. https://doi.org/10.7554/eLife.29123.

3) Manz, T. D.; Sivakumaren, S. C.; Ferguson, F. M.; Zhang, T.; Yasgar, A.; Seo, H. S.; Ficarro, S. B.; Card, J. D.; Shim, H.; Miduturu, C. V.; Simeonov, A.; Shen, M.; Marto, J. A.; Dhe-Paganon, S.; Hall, M. D.; Cantley, L. C.; Gray, N. S. Discovery and Structure-Activity Relationship Study of (z)-5-Methylenethiazolidin-4-One Derivatives as Potent and Selective Pan-Phosphatidylinositol 5-Phosphate 4-Kinase Inhibitors. J. Med. Chem. 2020, 63 (9), 4880–4895. https://doi.org/10.1021/acs.jmedchem.0c00227.

4) Manz, T. D.; Sivakumaren, S. C.; Yasgar, A.; Hall, M. D.; Davis, M. I.; Seo, H. S.; Card, J. D.; Ficarro, S. B.; Shim, H.; Marto, J. A.; Dhe-Paganon, S.; Sasaki, A. T.; Boxer, M. B.; Simeonov, A.; Cantley, L. C.; Shen, M.; Zhang, T.; Ferguson, F. M.; Gray, N. S. Structure-Activity Relationship Study of Covalent Pan-Phosphatidylinositol 5-Phosphate 4-Kinase Inhibitors. ACS Med. Chem. Lett. 2020, 11 (3), 346–352. https://doi.org/10.1021/acsmedchemlett.9b00402.

5) Dexheimer, T. S.; Rosenthal, A. S.; Luci, D. K.; Liang, Q.; Villamil, M. A.; Chen, J.; Sun, H.; Kerns, E. H.; Simeonov, A.; Jadhav, A.; Zhuang, Z.; Maloney, D. J. Synthesis and Structure-Activity Relationship Studies of N -Benzyl-2-Phenylpyrimidin-4-Amine Derivatives as Potent Usp1/Uaf1 Deubiquitinase Inhibitors with Anticancer Activity against Nonsmall Cell Lung Cancer. J. Med. Chem. 2014, 57 (19), 8099–8110. https://doi.org/10.1021/jm5010495.

6) Young, R. J.; Green, D. V. S.; Luscombe, C. N.; Hill, A. P. Getting Physical in Drug Discovery II: The Impact of Chromatographic Hydrophobicity Measurements and Aromaticity. Drug Discov. Today 2011, 16 (17–18), 822–830. https://doi.org/10.1016/j.drudis.2011.06.001.

7) Valkó, K. Chromatographic Hydrophobicity Index by Fast-Gradient RP-HPLC: A High-Throughput Alternative to Log P/Log D. Anal. Chem. 1997, 69 (12), 2022–2029. https://doi.org/10.1021/ac961242d.
